# Evolution, substrate specificity and subfamily classification of glycoside hydrolase family 5 (GH5)

**DOI:** 10.1186/1471-2148-12-186

**Published:** 2012-09-20

**Authors:** Henrik Aspeborg, Pedro M Coutinho, Yang Wang, Harry Brumer, Bernard Henrissat

**Affiliations:** 1 Division of Glycoscience, School of Biotechnology, KTH - Royal Institute of Technology, AlbaNova University Center, Stockholm SE-106 91, Sweden; 2 Michael Smith Laboratories and Department of Chemistry, University of British Columbia, 2185 East Mall, Vancouver V6T 1Z4, Canada; 3 Architecture et Fonction des Macromolécules Biologiques, Aix-Marseille Université, CNRS, UMR 7257, 163 Avenue de Luminy, Marseille 13288, France

**Keywords:** Protein evolution, Enzyme evolution, Functional prediction, Glycogenomics, Glycoside hydrolase family 5, Phylogenetic analysis, Subfamily classification

## Abstract

**Background:**

The large Glycoside Hydrolase family 5 (GH5) groups together a wide range of enzymes acting on β-linked oligo- and polysaccharides, and glycoconjugates from a large spectrum of organisms. The long and complex evolution of this family of enzymes and its broad sequence diversity limits functional prediction. With the objective of improving the differentiation of enzyme specificities in a knowledge-based context, and to obtain new evolutionary insights, we present here a new, robust subfamily classification of family GH5.

**Results:**

About 80% of the current sequences were assigned into 51 subfamilies in a global analysis of all publicly available GH5 sequences and associated biochemical data. Examination of subfamilies with catalytically-active members revealed that one third are monospecific (containing a single enzyme activity), although new functions may be discovered with biochemical characterization in the future. Furthermore, twenty subfamilies presently have no characterization whatsoever and many others have only limited structural and biochemical data. Mapping of functional knowledge onto the GH5 phylogenetic tree revealed that the sequence space of this historical and industrially important family is far from well dispersed, highlighting targets in need of further study. The analysis also uncovered a number of GH5 proteins which have lost their catalytic machinery, indicating evolution towards novel functions.

**Conclusion:**

Overall, the subfamily division of GH5 provides an actively curated resource for large-scale protein sequence annotation for glycogenomics; the subfamily assignments are openly accessible via the Carbohydrate-Active Enzyme database at http://www.cazy.org/GH5.html.

## Background

Carbohydrates, in the form of mono-, di-, oligo-, and polysaccharides, as well as glycoconjugates, play fundamental roles in all forms of life [1]. Beyond their role in energy storage, carbohydrates are central to diverse biological processes such as host-pathogen interactions, signal transduction, inflammation, intracellular trafficking, diseases, and differentiation/development. Not least, as structural components of terrestrial biomass, carbohydrates comprise approximately 75% of the carbon fixed annually by primary production [2]. Sugar-rich plant cell walls, seeds, and tubers thus represent a renewable material with significant potential to address energy and material needs.

A striking feature of carbohydrates is their remarkable structural complexity, due to a rich diversity of monosaccharide building blocks, and the possibility of numerous stereo- and regiospecific linkages [3], which give rise to both simple linear and complex, highly branched molecules [1]. A decade of investments in genomics and proteomics has greatly improved our interpretation of the molecular language of the cell, but deciphering the complex carbohydrate-based information in the biomolecular landscape is still in its infancy. Indeed, glycomics has been identified both as “the last frontier of molecular and cellular biology” [4] as well as an “emerging technology that will change the world” [5].

Functional analysis of glycans and glycoconjugates is complicated by the fact that they are not direct genetic products, but are instead synthesized, recognized, modified, and degraded by a plethora of carbohydrate-active enzymes (CAZymes) and binding proteins. In the synthetic direction, phosphosugar-dependent glycosyltransferases (GTs) catalyze the formation of glycosidic linkages, whereas their breakdown is mediated by glycoside hydrolases (GHs) and polysaccharide lyases (PLs), with the assistance of carbohydrate esterases (CEs). The structural diversity of carbohydrates is reflected in an abundance of CAZyme-encoding genes, which comprise 1-3% of the genome of most organisms [6]. Expanding and harnessing knowledge of the complexity of the “CAZome” is thus essential to understanding the complexity of the glycome.

The protein sequence-based classification of CAZymes was initiated in 1991 as a complement to the long-standing Enzyme Commission (EC) number system [7], which is based solely on enzyme activities [8]. Given the prevalence of convergent evolution of enzymes that cleave glycosidic bonds, as well as the demonstrable catalytic promiscuity of individual enzymes, sequence-based classification has proven to be a robust way to unify information on enzyme structure, specificity, and mechanism, which provides enormous predictive power [9]. Initially motivated by a need to delineate cellulases (EC 3.2.1.4) into distinct structural families [10], the first incarnation of the GH family classification, as such, comprised 35 GH families [8]. The number of families increased steadily with the growing interest in Glycobiology so that, as of August 2012, 130 sequence-based families of GHs have been defined in the continuously updated CAZy database [11].

Presently, one of the largest GH families is GH5, historically known as “cellulase family A” as it was the first cellulase family described [10]. GH5 exemplifies a family with a large variety of specificities: it currently contains close to 20 experimentally determined enzyme activities denoted with an EC number. The abundance of GH5 enzymes in different ecological niches has been highlighted by their frequent identification in metagenomes of diverse microbial communities [12-14], as well as the genomes of individual organisms [11]. As with other CAZyme families [15], GH5 members are commonly found to be encoded as parts of multi-modular polypeptide chains containing other catalytic, substrate-binding, and functionally unidentified or yet to be described modules.

Within the large GH5 family, a discernible diversity of sequences was observed soon after its creation. The first five subfamilies of GH5 (A1-A5) were identified as early as 1990 [16]. Subfamily A6 was introduced in 1997 [17] and the following year eukaryotic and prokaryotic β-mannanases were assigned to A7 and A8, respectively [18]. Subsequently, subfamily A9 was introduced in a study, which notably also suggested the merger of A5 and A6 [19]. Finally, A10 was the most recently defined GH5 subfamily [20], while new subfamilies that presently lack a unique identifier have also been suggested [21,22]. Family GH5 belongs to clan GH-A, which presently groups 19 GH families to form the largest set of evolutionarily related GH families described in CAZy thus far (a *clan* is a group of *families* that arise from a common but very distant ancestor; despite weak sequence similarity, clan members share conserved protein fold and catalytic machinery).

Families such as GH5 were originally defined with a very small number of sequences. With the accumulation of an increasing body of sequence data, the relationship between the original families has sometimes changed enough to merit reexamination of family membership. Very recently, detailed three-dimensional structural analysis led to the reclassification of several GH5 sequences into family GH30 based on the organization of secondary structural elements around the conserved (β/α)_8_ fold of the catalytic module [23].

Given the continuing expansion in sequence numbers and the partial GH5/GH30 reclassification, it is clear that a global re-analysis of the subfamily division of GH5 is now needed. The rapid accumulation of genomic data in the past decade revealed a complex and varied sequence space, with the consequence that a substantial portion of GH5 family members are currently not assigned to any subfamily. This situation will only become worse as the rate of (meta)genomic sequencing continues to increase with phenomenal rapidity. Further, this flood of data will cause an increasing reliance on computer-based annotation, which necessarily requires a robust framework to produce meaningful functional predictions. The division of CAZyme families into subfamilies based on phylogenetic analysis has been applied as a successful approach to meet this challenge: Subfamily classification of GH13, GH30 and all of the PL families has demonstrated that the majority of the defined subfamilies were monospecific, thus indicating a significantly better correlation of substrate specificity between sequences at the subfamily level than the family level [23-25]. Significantly, the division into subfamilies allows the identification of currently uncharacterized subfamilies that can subsequently be analyzed biochemically and structurally to potentially unveil new activities.

Hence, we present here an improved, robust subfamily classification for GH5 by employing a large-scale analysis of all publicly available sequences. Our intention is that the introduction of this additional hierarchical level across this important GH family will serve to guide enzyme discovery, structure-function analysis, and biocatalyst improvement in post-genomic efforts. Not least, many enzyme activities relevant to biomass analysis and conversion are found in GH5 (e.g., cellulases, mannanases, xylanases, galactanases, and xyloglucanases), as are enzymes with biomedical applications [26]. Significantly, the present analysis unveiled a large number of sparsely or incompletely characterized subfamilies that may still hide a number of unsuspected activities and singular structural features.

## Results and discussion

**Figure 1:**
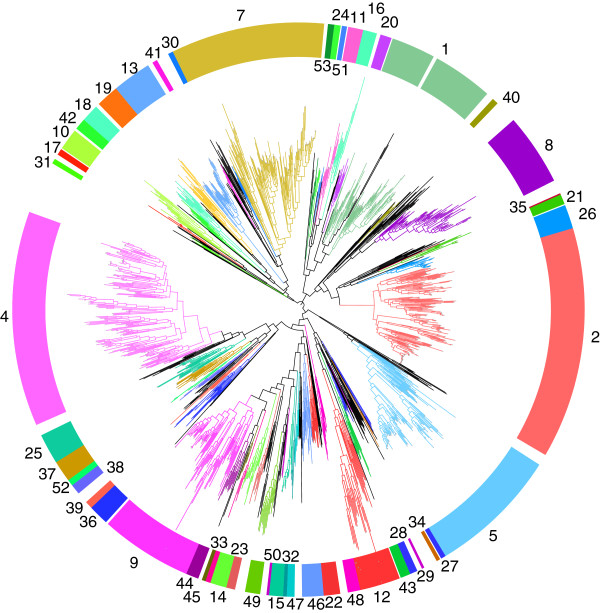
**Phylogenetic tree of family GH5.** In this circular phylogram, the branches corresponding to subfamilies 1–53 are shown in color and the subfamily numbers are indicated next to the exterior color circle. The branches corresponding to sequences not included into subfamilies are in black. A detailed version of this tree is found in Additional file 1: Figure S1.

Our bioinformatics approach allowed the division of close to 2300 GH5 catalytic modules into 51 distinct subfamilies, as shown in the global phylogenetic tree (Figure 1 and Additional file 1: Figure S1); subfamily information is summarized in Table 1. Subfamily naming follows the procedure devised for GH13, where the family number is followed by an Arabic numeral that reflects the order of creation [24]: GH5_1 to GH5_53. This series is essentially continuous, with a few exceptions due to historical reasons: All of the previously described subfamilies (A1-A10) have been re-identified in the current investigation except for A3 and A4, which are merged into a single subfamily GH5_4 and A5 and A6 which are unified in subfamily GH5_5 (Figure 1). To maintain consistency with earlier literature, the re-identified historical subfamilies have retained the original Arabic numeral. For example, the subfamily formerly known as A2 is hereby designated GH5_2. The absence of subfamilies GH5_3 and GH5_6 reflects the two fusion events involving the historical subfamilies described above [19].

**Table 1:** Newly defined subfamilies within glycoside hydrolase family GH5

**New GH5 subfamily**	**Historical subfamily**	**Number of sequences**	**Present taxonomical distribution**	**EC number**	**Representative PDB structure**
GH5_1	A1^b^	133	*Archaea Bacteria Eukaryota*	3.2.1.4	2ZUN
				3.2.1.73	
				3.2.1.91	
GH5_2	A2^b^	245	*Bacteria Eukaryota*	3.2.1.4	2A3H
				3.2.1.132	
GH5_4	A3 + A4^b^	160	*Bacteria Eukaryota*	3.2.1.4	2JEQ
				3.2.1.151	
				3.2.1.73	
				3.2.1.8	
GH5_5	A5 + A6^b,^^c,^^e^	123	*Bacteria Eukaryota*	3.2.1.4	IGZJ
GH5_7	A7^d^	133	*Archaea Bacteria Eukaryota*	3.2.1.25	IRH9
				3.2.1. 78	
				2.4.1.-	
GH5_8	A8^d^	71	*Bacteria Eukaryota*	3.2.1.78	2WHL
GH5_9	A9^e^	107	*Eukaryota*	3.2.1.58	3N9K
				3.2.1.75	
				3.2.1.21	
GH5_10	A10^e,f^	19	*Bacteria Eukaryota* (Metazoa)	3.2.1.78	2C0H
GH5_11		19	*Eukaryota* (Plants;Fungi)	ND	
GH5_12		42	*Bacteria Eukaryota*	3.2.1.21	
				3.2.1.45	
GH5_13		59	*Bacteria*	ND	
GH5_14		15	*Eukaryota* (Plants)	3.2.1.58	
GH5_15		10	*Eukaryota* (Fungi)	3.2.1.75	
GH5_16		10	*Eukaryota* (Fungi)	3.2.1.164	
GH5_17		5	*Bacteria*	3.2.1.78	
GH5_18		24	*Bacteria*	ND	
GH5_19		23	*Archaea*	ND	
GH5_20		17	*Eukaryota* (Stramenopiles)	ND	
GH5_21		10	*Bacteria*	3.2.1.8	
GH5_22		12	*Bacteria Eukaryota*	3.2.1.4	
GH5_23		5	*Eukaryota* (Fungi)	3.2.1.149	
				3.2.1.168	
GH5_24		5	*Eukaryota* (Fungi)	ND	
GH5_25		16	*Bacteria*	3.2.1.4	3MMW
				3.2.1.78	
GH5_26		17	*Bacteria*	3.2.1.4	
				3.2.1.73	
GH5_27		5	*Eukaryota* (Metazoa;Fungi)	3.2.1.123	
GH5_28		8	*Bacteria*	3.2.1.123	2OSX
GH5_29		5	*Bacteria*	3.2.1.123	
GH5_30		5	*Eukaryota* (Fungi)	ND	
GH5_31		5	*Eukaryota* (Fungi)	3.2.1.*-*^g^	
GH5_32		5	Eukaryota (Plants; Stramenopiles)	ND	
GH5_33		9	Eukaryota (Stramenopiles)	ND	
GH5_34		5	*Bacteria*	3.2.1.*-*^g^	2Y8K
GH5_35		5	*Bacteria*	ND	
GH5_36		23	*Bacteria*	3.2.1.73	IVJZ
				3.2.1.78	
GH5_37		19	*Bacteria*	3.2.1.4	ICEN
				3.2.1.73	
				3.2.1.74	
GH5_38		10	*Bacteria*	3.2.1.*-*^h^	
GH5_39		7	*Bacteria*	3.2.1.4	
GH5_40		8	*Bacteria*	ND	
GH5_41		14	*Bacteria*	ND	
GH5_42		9	*Bacteria*	ND	
GH5_43		11	*Bacteria*	ND	
GH5_44		24	*Bacteria*	ND	
GH5_45		5	*Bacteria*	ND	
GH5_46		15	*Bacteria*	3.2.1.*-*^h^	
GH5_47		6	*Bacteria*	ND	
GH5_48		18	*Bacteria*	3.2.1.*-*^h^	
GH5_49		20	*Eukaryota* (Fungi)	ND	
GH5_50		7	*Eukaryota* (Fungi)	ND	
GH5_51		5	*Bacteria Eukaryota* (Fungi)	ND	
GH5_52		6	*Bacteria*	3.2.1.74	
GH5_53		6	*Bacteria*	3.2.1.74	

ND Activity not determined yet.

^a^Experimentally determined.

^b^see [16].

^c^see [17].

^d^see [18].

^e^see [19].

^f^see [20].

^g^EC numbers not yet defined.

^h^Active enzyme(s) present but with unclear EC number(s).

* See http://www.cazy.org for most recent information.

Historical names for some subfamilies are provided, along with the taxonomical range, the characterization level and structural information from representative structures. Known enzyme activities in family GH5 are provided using the following Enzyme Classification (EC) numbers and corresponding activities: 3.2.1.4 – endo-β-1,4-glucanase or cellulase; 3.2.1.8 – endo-β-1,4-xylanase; 3.2.1.21 – β-glucosidase; 3.2.1.25 – β-mannosidase; 3.2.1.45 – β-glucocerebrosidase; 3.2.1.58 – glucan β-1,3-glucosidase; 3.2.1.73 – licheninase; 3.2.1.74 – cellodextrinase; 3.2.1.75 – glucan endo-β-1,6-glucosidase; 3.2.1.78 – mannan endo-β-1,4-mannosidase or endo-β-1,4-mannanase; 3.2.1.91 – cellulose β-1,4-cellobiosidase or cellobiohydrolase; 3.2.1.123 – endoglycoceramidase; 3.2.1.132 – chitosanase; 3.2.1.149 – β-primeverosidase; 3.2.1.151 – xyloglucan-specific endo-β-1,4-glucanase; 3.2.1.164 – endo-β-1,6-galactanase; 3.2.1.168 – hesperidin 6-O-α-L-rhamnosyl-β-glucosidase; 3.2.1.- – undefined EC numbers for β-1,3-mannanase/β-1,3-glucomannanase or for arabinoxylan-specific β-xylanase or still unclear EC numbers depending on the subfamily (see notes and text); 2.4.1.- – β-mannan transglycosidase.

In addition to the new and historical designations, the taxonomical range of the included sequences, experimentally determined enzyme activities and representative 3-D structures are presented in Table 1 for each subfamily. Notably, all of the 33 enzymes with a solved 3-D structure have been assigned to a subfamily, resulting in thirteen individual subfamilies out of 51 with at least one structural representative. Genes that encode GH5 enzymes are present in most organisms ranging from *Archaea* and *Eubacteria* to *Eukaryotes*, e.g. fungi and plants. From an anthropocentric perspective, a GH5 member is notably lacking in the human genome. Examples of metazoan GH5 genes are also notably scarce and are limited to nematodes, mollusks, and arthropods, likely resulting from horizontal transfer. For example, several independent horizontal events of transfer of cellulase and xylanase genes from Bacteria to nematodes have been described [27] and the transfer of a bacterial β-mannanase to an insect was recently documented [28]. The taxonomical range at the subfamily level is, naturally, more restricted. A few smaller subfamilies are currently specific to certain types of organisms (Table 1). For example, eight subfamilies (GH5_15, GH5_16, GH5_23, GH5_24, GH5_30, GH5_31, GH5_49 and GH5_50) contain only fungal sequences. Subfamily GH5_14 contains only plant members, whereas members of GH5_20 come exclusively from *Stramenopiles*. These limited taxonomic distributions may represent biological (catalytic) specialization, if not biased by a still incomplete genome sequencing of organisms.

The definition of subfamilies was restricted to phylogenetic clades with five or more members from different organisms available in the public protein databases (GenBank and UniProt) in order to capture sufficient diversity for a robust subfamily definition. Using these criteria, the overall success rate of subfamily grouping was of approximately 80%, i.e., about 20% of the analyzed GH5 sequences could not be classified into subfamilies having at least five public members. The sequences that have not yet been assigned to subfamilies will likely define new subfamilies as the pool of available sequences continues to increase. In the future, these subfamilies will be gradually released with new identifiers when they have been sufficiently populated. Compared to the GH13 subfamily classification [24], the GH5 subdivision has resulted in both a higher number of subfamilies and a higher number of uncharacterized subfamilies, suggesting that family GH5 is comparatively less well explored.

 To further refine the global subfamily analysis, maximum likelihood phylogenetic analysis has been performed on each subfamily (Additional file 1: Figures S1: GH5_1 – GH5_53). In addition to database accession numbers, information about substrate specificity (as provided by EC numbers) has been included, and 3-D structures highlighted in each subfamily tree. The 51 subfamilies (numbered GH5_1 to GH5_53 as explained elsewhere) have been categorized based on available enzyme activity data, to aid their individual descriptions, below. Thus, the first group, comprised of “Enzymatically-Active Subfamilies”, contains subfamilies with at least one member whose enzyme activity has been shown. The extent of the documented characterization varies substantially within the group, from simple information obtained from enzymatic assays insufficient to assign a particular EC number (these enzymes are denoted with a star in Additional file 1: Figure S1), to detailed enzyme specificity and kinetics studies. Among the better characterized subfamilies, monospecific subfamilies are distinguished by the presence of only one EC number for one or more members, whereas in polyspecific subfamilies, two or more enzymatic activities have been observed in different members. “Uncharacterized Subfamilies” comprise the second major group; these subfamilies currently lack documented enzymatic activity altogether.

In this context, it is worth noting that the majority of the large, well characterized subfamilies were polyspecific. These highly populated subfamilies were also the first ones to be identified and described, and often, despite observed polyspecificity, one particular activity predominates. For example, 22 of 24 characterized enzymes in GH5_1 are reported to be *endo*-glucanases, whereas one protein is a documented as a cellobiohydrolase, and another was described as displaying licheninase activity. It is, however, difficult to draw far-reaching conclusions based on these observations. On one hand, it may be that the acquisition of a new specificity within a subfamily is a rare event; alternatively, the observation of one or few activities in specific subfamilies may simply be a consequence of differences in the range of substrates tested experimentally.

**Figure 2:**
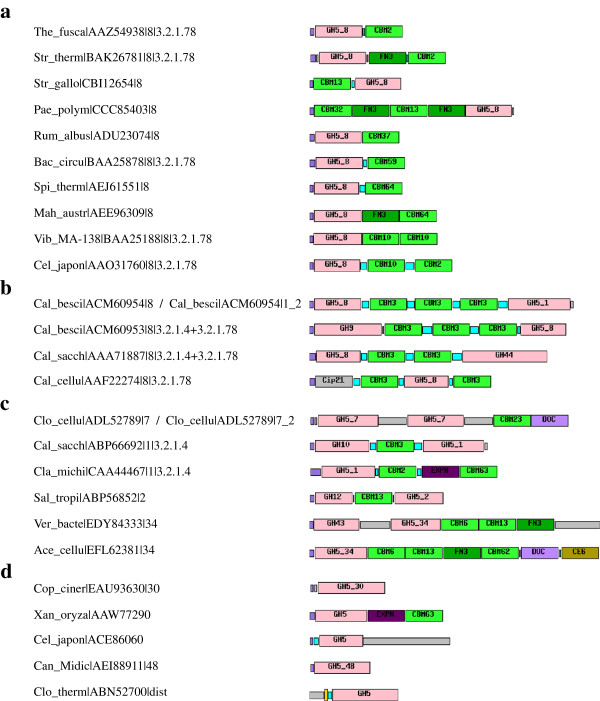
**Examples of modular GH5 proteins.** (**a**) Diverse modular arrangements of putative monofunctional modular enzymes from subfamily GH5_8. (**b**) Same for putative bifunctional GH5 enzymes containing a subfamily GH5_8 module. (**c**) Other putative bifunctional enzymes containing at least a single GH5 module. (**d**) Selected examples of proteins containing GH5 modules having lost one or more catalytic residues. For a given protein, each GH5 module is identified by a number of fields separated by “|” indicating: (i) the organism, with 3 letters for the genre and either 5 letters for the species or full strain code; (ii) the GenBank protein accession; (iii) if attributed, the subfamily number or other information; (iv) EC numbers if available. These individual tags are analogous to what is found in Additional file 1: Figure S1. The module types and other protein segments present are: GHx_y – glycoside hydrolase family *x* subfamily *y* (pink); CEx – carbohydrate esterase module of family *x* (light brown); Cip21 – chitin-binding protein type 21 module with putative carbohydrate oxidative cleaving activity, formerly CBM33 (dark gray); CBMx – carbohydrate binding modules of family *x* (light green); FN3 – fibronectin type III modules (dark green); DOC – cellulosomal dockerin modules (light violet); EXPN – expansin modules (dark purple); signal peptides (purple); transmembrane segments (yellow); linkers (light blue); other regions (light grey).

Another significant aspect of many GH5 family proteins is that their protein sequence may include additional modules with different functions, and in particular CBMs [29]. A great variety of modular structures may be found throughout the family and in a number of individual subfamilies. An analysis of all the complexity of modular structures found in the family goes beyond the objectives of this study, and some aspects of this diversity are illustrated in Figure 2. For instance, many members of subfamily GH5_8 are modular and reveal two major trends: (i) the addition of one or of multiple CBMs (see Figure 2a) is more common and may be associated not only to the nature of the main substrate of the corresponding catalytic domain, particularly in complex substrates; and (ii) the combinations with other catalytic modules to form bifunctional enzymes (see Figure 2b but also 2c), are more rare but particular useful to reveal interacting or synergistic enzyme activities of some catalytic modules. Numerous modular arrangements can also be found in other large subfamilies like GH5_1, GH5_2 and GH5_7. CBMs can be located on the N- or C-terminal side of the GH5 module (as illustrated in GH5_8 in Figure 2a). The combination of catalytic domains may target different tissue components. Some may, for instance, target cellulose and cellulose associated substrates (as in Figure 2b) but bifunctional enzymes likely targeting hemicellulose may also be found (several examples in Figure 2c).

### Subfamilies with identified active enzymes

#### Monospecific subfamilies

A number of subfamilies exhibit a single activity among their characterized enzyme members. Multiple individual examples within a subfamily improve the degree of confidence regarding subfamily monospecificity, while subfamilies with only a single characterized representative may be subject to reinterpretation in the future as the breadth of biochemical data increases.

##### GH5_5 *endo*-β-1,4-glucanases (EC 3.2.1.4)

The largest subfamily that contains only a single EC number is GH5_5, which is primarily composed of secreted bacterial and fungal enzymes (Additional file 1: Figure S1: GH5_5). All investigated enzymes in GH5_5 display *endo*-β-1,4-glucanase activity (EC 3.2.1.4). One crystal structure has been determined for a *Thermoascus aurantiacus endo*-glucanase [19]. In fungi, about half of the GH5 proteins harbor a CBM1 module at the N- or C-terminus, which is compatible with an active role on cellulose. No modular proteins are found among the bacterial members of the subfamily.

##### GH5_8, GH5_10 and GH5_17: *endo*-β-1,4-mannanase (EC 3.2.1.78)

Members of the large GH5_8 subfamily are all extracellular mannan *endo*-β-1,4-mannosidases (EC 3.2.1.78) according to available biochemical characterization, and this subfamily was historically described as the bacterial mannanase subfamily A8 [18]. Structural analysis has highlighted distinctive features of alkaline β-mannanases [30]. The subfamily now contains a single eukaryotic enzyme from the beetle *Hypothenemus hampai*, resulting from a horizontal gene transfer from bacteria [28].

In the closely related but distinct subfamilies GH5_10 and GH5_17, only extracellular enzymes with *endo*-β-1,4-mannanase activity (EC 3.2.1.78) have equally been reported. Metazoan sequences, including sequences originating from mollusks and arthropods, as well as bacterial sequences compose the GH5_10 subfamily, whereas subfamily GH5_17 only harbors bacterial enzymes. Currently, none of the bacterial GH5_10 members have a documented enzyme activity, although a *Fibrobacter succinogenes* enzyme is active on AZCL-galactomannan [31]. The bacterial enzymes in GH5_17 are all cellulosomal components from the genus *Clostridium. *

##### GH5_14 : (plant) *exo*-β-1,3-glucosidase (EC 3.2.1.58)

Subfamily GH5_14 comprises exclusively plant enzymes and relies on a single functional characterization at present. Recombinant expression of the rice OsGH5BG showed that the enzyme had glucan β-1,3-glucosidase activity (EC 3.2.1.58) [22]. A unique feature of GH5_14 is the fascin-like module inserted after β-strand 1. Fascin is a human actin-binding protein, but the function of the plant fascin-like domain is unknown. Members of GH5_14 are well represented throughout the plant kingdom, but most interestingly, a representative is absent in the leading plant model organism *Arabidopsis thaliana*.

##### GH5_15 : (fungal) (*endo*-)β-1,6-glucanases (EC 3.2.1.75)

Subfamily GH5_15 is a small but well characterized subfamily composed of secreted fungal enzymes. The identified β-1,6-glucanase activity (EC 3.2.1.75) is important for the mycoparasitic activity and probably cell wall recycling by some fungi. The GH5_15 phylogenetic tree displays two major clades (Additional file 1: Figure S1: GH5_15). The largest clade is formed by enzymes issued from fungi from the class of *Sordariomycetes*. *Eurotiomycetes* are found the second subgroup.

##### GH5_16 : (fungal) *endo*-β-1,6-galactanase (EC 3.2.1.164)

GH5_16 is another example of a monospecific subfamily of secreted enzymes where only a single fully sequenced biochemically characterized β-1,6-galactanase (EC 3.2.1.164) is currently known [32], although partial N-terminal sequence of an *Aspergillus* enzyme, closely related to sequences of other members of the subfamily, yields the same activity [33]. Several other enzymes with β-1,6-galactanase activity have been moved from GH5 to GH30_5 [23], but subfamily GH5_16 clearly remains within family GH5. The known β-1,6-galactanase (EC 3.2.1.164) is involved in larch wood arabinogalactan degradation [32].

##### GH5_21 : (bacteroidetes) *endo*-β-1,4-xylanase (EC 3.2.1.8)

Xylanase (EC 3.2.1.8) activity has been recently established [34] for a number of xylanolytic bacteroidetes enzymes belonging to this subfamily. These GH5_21 *endo*-xylanases integrate xylan utilization gene clusters found in *Prevotella* and *Bacteroides* species and are all apparently secreted. Significant differences in their mode of action have been observed, despite the inclusion in the same subfamily. Different GH5_21 enzymes were shown to release different products from wheat arabinoxylan [34].

##### GH5_22 GH5_31, GH5_34, GH5_39, and GH5_53 : single β-glycanase characterizations

In addition to subfamilies GH5_14 and GH5_16 described above, five other subfamilies are distinguished by harboring only a single experimentally characterized enzyme. *Endo*-β-1,4-glucanase activity (EC 3.2.1.4) has been determined in subfamilies GH5_22 and GH5_39. GH5_31 is a small subfamily currently restricted to secreted fungal proteins. A β-1,3-(gluco)mannanase activity (EC 3.2.1.-)^a^ has been reported for an enzyme from *Paecilomyces lilacinus*[35]. In the small subfamily GH5_34, composed of extracellular modular proteins from bacterial origin, there is a single enzymatically and structurally characterized enzyme from *Clostridium thermocellum*. Notably, although this is the first reported enzyme with arabinoxylanase activity (EC 3.2.1.-)^b^ it was designated *Ct*Xyl5A in spite of its inability to attack unsubstituted xylans [36]. Finally, the small subfamily GH5_53 is a modular extracellular subfamily that contains a single characterized cellodextrinase (EC 3.2.1.74) [37].

##### GH5_27 GH5_28 and GH5_29 : *endo*-glycosylceramidases (EC 3.2.1.123)

GH5_27, GH5_28 and GH5_29 are subfamilies exclusively containing extracellular *endo*-glycosylceramidases. Subfamily GH5_27 is formed of sequences of eukaryotic origin while the small subfamilies GH5_28 and GH5_29 are bacterial. Interestingly, the first subfamily is found among the four subfamilies that contain metazoan GH5 enzymes. All but one of the enzymes found in GH5_28 are from *Actinobacteria*. The crystal structure of a *Rhodococcus endo-*glycoceramidase revealed an active site channel atypical for GH5 enzymes, which explains the unusual substrate for this type of enzymes [38]. All the GH5_29 sequences reported here are from the genus *Rhodococcus.* The characterized bacterial enzymes of GH5_28 hydrolyze ganglio- and lacto-series glycosphingolipids. In contrast, the only GH5_29 enzyme investigated is not capable of hydrolyzing these substrates. Instead, this enzyme shows activity against 6-gala series glycosphingolipids and the designation oligogalactosyl-N-acylsphingosine 1,1’-β-galacto-hydrolase has been proposed [39].

##### GH5_52 : cellodextrinases (EC 3.2.1.74)

Two enzymes in the small intracellular bacterial GH5_52 subfamily exhibited cellodextrinase activity (EC 3.2.1.74). In addition two enzymes isolated from the cow rumen have been shown to have hydrolytic activity on carboxymethyl cellulose (CMC) agar [14].

#### Polyspecific subfamilies

Some GH5 subfamilies group together a panel of activities and are described as polyspecific subfamilies. The apparent plasticity of these subfamilies suggests that only few subtle changes could be sufficient to switch from one activity to the other. More likely, many of the enzymes present on the subfamily level are polyspecific to some extent and therefore should have more than a single EC attributed.

##### GH5_1 and GH5_2 : β-1,4-glucan cleaving enzymes

Extracellular enzymes from archaea, bacteria and uncultured symbiotic protists are represented in subfamily GH5_1 (Additional file 1: Figure S1: GH5_1). The activity observed for most characterized GH5_1 enzymes is *endo*-β-1,4-glucanase activity (EC 3.2.1.4). The apparent exception in subfamily GH5_1 is an *exo*-acting cellobiohydrolase activity (EC 3.2.1.91) from *Clostridium thermocellum*[40]. However, one should note that the biochemical distinction of *exo*- versus *endo*-acting cellulases is particularly difficult to establish experimentally. Interestingly, an enzyme from *Ruminococcus albus* able to cleave CMC and glucomannan but particularly active on lichenin was recently described [41]. Many proteins in this subfamily are modular (data not shown), a feature shared with many members of subfamily GH5_8 as described previously.

Subfamily GH5_2 is currently the largest in family GH5. This subfamily of extracellular enzymes, many of which are multimodular, contains a large number of characterized members that display *endo*-β-1,4-glucanase activity (EC 3.2.1.4). These *endo*-glucanases are distributed across the subfamily tree and are found in every major clade Additional file 1: Figure S1: GH5_2). One *endo*-glucanase from *Fibrobacter succinogenes* S85 in this subfamily was reported to be active both on CMC and oat spelt xylan [42], but xylanase activity has not been observed in other members thus far. Interestingly, one representative of this subfamily has been reported as a chitosanase (EC 3.2.1.132) with transglycosylation activity [43]. A bifunctional cellulase/chitosanase has also been identified in *Bacillus sp.* NBL420 [44] in a different clade. Significantly, a closely related N-terminal sequence of a bifunctional cellulase/chitosanase from *Myxobacter sp. AL-1*[45], suggests that a specific subgroup bearing both activities may exist. As for subfamily GH5_1, many members of this subfamily are multimodular, having both CBMs and cellulosomal-like dockerins.

##### GH5_4 : (xyloglucan-specific) *endo*-β-1,4-glucanases (EC 3.2.1.4 and EC 3.2.1.151), licheninases (EC 3.2.1.73), and xylanases (EC 3.2.1.8)

Subfamily GH5_4 members are typically extracellular bacterial enzymes, although some members come from ciliates and fungi, predominantly rumen organisms. In total, four enzyme activities have been reported for subfamily GH5_4. Thus far, GH5_4 is the only subfamily containing enzymes with reported xyloglucanase activity (EC 3.2.1.151) [46]. Interestingly, the xyloglucanases are found in two different clades of the GH5_4 subfamily tree suggesting that the switch of enzyme activity inside this subfamily occurred at different times. (Additional file 1: Figure S1: GH5_4). Licheninases (EC 3.2.1.73) have also been described in this subfamily. Significantly, *endo*-β-1,4-xylanase activities (EC 3.2.1.8) have been reported for a few enzymes, but always in conjunction with other activities. For example, the xylan degrading specific activity of *Clostridium cellulovorans* EngB and EngD are both of approximately 14% of their respective specific activities on lichenan [47]. Such features suggest an important degree of enzyme promiscuity given the structural similarity of the β-linked substrates. However, the most commonly reported EC number for representatives of GH5_4 is EC 3.2.1.4. Except for a few fungal pathogen members of the subfamily that bear a CBM1 module, no other known CBM is present, in sharp contrast to what has been found for extracellular subfamilies GH5_1 and GH5_2.

##### GH5_7 : β-1,4-mannan-cleaving enzymes (EC 3.2.1.78 and EC 3.2.1.25)

In subfamily GH5_7, previously named A7 (originally comprised of only eukaryotic mannanases but now also containing archaeal and bacterial members) [18], the three reported enzyme activities are associated to the degradation or to the modification of β-mannan-containing polysaccharides. Virtually all examined GH5_7 enzymes possess *endo*-β-1,4-mannanase activity (EC 3.2.1.78). One exception is the tomato protein LeMan4a, which in addition to hydrolytic activity, can act *in vitro* as a mannan transglycosylase (EC 2.4.1-) [48]. Recently, mannan transglycosylase activity was also reported for two fungal GH5_7 enzymes [49]. It is not impossible that further GH5_7 enzymes may reveal a transglycosylase activity in the future, since the distinction between hydrolytic and transglycosylase activity is dictated by the tendency of the glycosyl-enzyme intermediate to be intercepted by a water molecule or a saccharide molecule, respectively. Another interesting case among GH5_7 members is the β-mannosidase *Cm*Man5A, which is able to release mannose from the non-reducing end of manno-oligosaccharides and -polysaccharides (EC 3.2.1.25) and is thus *exo*- and not *endo*-acting. This difference was explained by the length of three loops which modify the active center accessibility [50]. Furthermore, this subfamily has both extracellular and intracellular enzymes. Many GH5_7 extracellular enzymes from fungi contain a CBM. In many bacteria, CBMs from different families are also found appended to GH5_7 catalytic modules (data not shown).

##### GH5_9 : fungal cell wall modifying enzymes

This subfamily contains only sequences of fungal origin putatively found in different cell locations: some are secreted, several present a GPI-anchor, others have single transmembrane segments and yet others appear to be intracellular. The activity *exo*-β-1,3-glucanase (EC 3.2.1.58) has been described for most of the characterized examples, all found among the apparently secreted enzymes. Structural investigation of the *Candida albicans* Exg protein provides a clue to the evolution of these *exo*-hydrolases; the structure reveals an active site pocket shaped for cleavage of β-1,3- but not β-1,4 glycosidic linkages [51]. Surprisingly, the periplasmic Exg1 protein from *Schizosaccharomyces pombe* was demonstrated to be an *endo*-β-1,6-glucanase (EC 3.2.1.75), while the membrane-anchored protein Exg2 protein was shown to produce cell wall material when over-expressed [52]. Two of the characterized GH5_9 *exo*-β-1,3-glucanases have been also described as β-glucosidases (EC 3.2.1.21) [53].

##### GH5_12 : β-glucosylceramidases (EC 3.2.1.45) and (flavonoid) β-glucosidase (EC 3.2.1.21)

Previously known to contain a flavonoid β-glucosidase (EC 3.2.1.21) from yeast [53], the panorama of specificities in this subfamily recently expanded to include several fungal β-glucosylceramidases (EC 3.2.1.45**)**[54]**.** The subfamily is grouped into a fungal clade, including three *Cryptococcus* sequences (one now characterized), and a clade dominated by bacterial enzymes (Additional file 1: Figure S1: GH5_12).

##### GH5_23 : fungal β-diglycosidases (EC 3.2.1.149 and EC 3.2.1.168)

 Subfamily GH5_23 is composed of secreted fungal proteins, two of which have been characterized as β-diglycosidases that break down plant diglycoconjugated flavonoids. Deglycosylation of these compounds most often involves the sequential action of two β-glycosidases in contrast to the one-step hydrolytic release of the disaccharide moiety from the aglycone by β-diglycosidases [55]. The characterized enzymes are a hesperidin 6-O-α-L-rhamnosyl-β-glucosidase (EC 3.2.1.168) from *Stilbella fimetaria* from which a partial sequence has been obtained [56] and a reported β-primeverosidase (EC 3.2.1.149) from *Penicillium multicolor* TS-5 [57]. This subfamily is present in the genera *Aspergillus* and *Penicillium* known for interaction with plants.

##### GH5_25 : *endo*-β-1,4-glycanases (EC 3.2.1.4 and EC 3.2.1.78)

Most enzymes found in subfamily GH5_25 are derived from thermophiles. Interestingly, characterized enzymes in this subfamily represent examples of GH5 enzymes with multiple activities. For instance, Cel5A from *Thermotoga maritima* exhibits activity on both β-mannan-based and β-glucan-based polymers. Analyses of the *Tm*Cel5A structure have highlighted features important both for the nature of the duality and the thermostability [58,59].

##### GH5_26 : *endo*-β-1,4-glycanases (EC 3.2.1.4 and EC 3.2.1.73)

GH5_26 is a small subfamily with a majority of sequences from uncultured microorganisms. The dominating activity found is *endo*-β-1,4-glucanase (EC 3.2.1.4), but one enzyme has also high activity against lichenan (EC 3.2.1.73) [60].

##### GH5_36 *endo*-b-1,4-glycanases (EC 3.2.1.73 and EC 3.2.1.78)

Several bacterial phyla are represented in subfamily GH5_36. One enzyme has a demonstrated *endo*-β-1,4-mannanase activity (EC 3.2.1.78), whereas a second enzyme exhibits licheninase activity (EC 3.2.1.73) in addition to β-mannanase activity [61,62]; a 3-D structure is available for the latter enzyme.

##### GH5_37 : *endo*-β-1,3/4-glycanases (EC 3.2.1.4 and EC 3.2.1.73) + cellodextrinase (EC 3.2.1.74)

Three different activities are found in subfamily GH5_37, which consists of sequences of bacterial origin encoding intracellular proteins. The majority of the characterized enzymes are cellulases (EC 3.2.1.4), but there are also examples of licheninase activity (EC 3.2.1.73) [41], and cellodextrinase activity (EC 3.2.1.74) [63].

##### GH5_38 and GH5_46 : bacterial enzymes active on model plant cell wall (PCW) compounds

In subfamily GH5_38, three enzymes isolated from rumen metagenomic projects have been partially characterized [12,14]. To these, we may add a *Prevotella ruminicola 23* enzyme described as cellulase (PRU_1856) and shown to be active against CMC, Avicel, and lichenan [64]. Another enzyme discovered in the microbial community of the cow rumen is active on CMC and belongs to subfamily GH5_46 [14]. This is the single evidence of activity in this subfamily.

##### GH5_48 : bacterial enzymes active on chitin and chitosan derivatives

Several bacterial phyla are currently represented in subfamily GH5_48, mostly composed of extracellular and membrane-anchored proteins of unknown function. A single member, a partial sequence (not shown in trees because incomplete) coding for a protein from *Pseudomonas putida P3(4))*, over 98% identical to locus PPS_1333 (GenBank accession AEJ11908) from *Pseudomonas putida S16,* has been described as a bifunctional enzyme as it was active on preparative forms of chitin and colloidal chitosan and on the model compounds pNP-β-N-acetylglucosaminide and 4-methylumbelliferyl-N-acetyl β-D-glucosaminide [65]**.** Given that chitosanases are already present in family GH5, related activities are not unexpected. Interestingly, one sequence in GH5_48 seems to lack the catalytic machinery (Additional file 1: Figure S1).

#### Subfamilies lacking experimental characterization

A large number of subfamilies still require evidence of enzyme activity (Table 1). A large characterization effort is still needed to identify the hidden activities of these subfamilies. For a number of subfamilies, particularly for those containing bacterial enzymes, direct hints of the activities to be tested can be obtained from the additional modules present, such as CBMs, but also from operon-like organizations. But the contribution of other more indirect approaches like transcriptomics and functional metagenomics using innovative sets of conditions and enlarged classes of substrates are equally potent to provide clues to enzyme function. The uncharacterized subfamilies are distributed across the GH5 phylogenetic tree. However, in one of the three major clades, a large block of uncharacterized subfamilies (GH5_13, GH5_18, GH5_19, GH5_30, GH5_41 and GH5_42) is located between characterized subfamilies containing β-mannan-acting enzymes (GH5_7, GH5_10, GH17, and GH5_31) (Figure 1). It is tempting to speculate that at least some of these uncharacterized subfamilies target substrates related to β-mannan.

#### Unclassified GH5 proteins

Roughly 20% of the analyzed GH5 sequences were not assigned to subfamilies, although some of these proteins have been characterized to different levels. The main reason for the inability to assign them to subfamilies was the lack of sufficiently related sequences to define a subfamily with at least five members. This is likely to change in the future due to a predicable increase in the number of available sequences and these unclassified sequences represent a pool out of which many more subfamilies will emerge. A total of 17 characterized enzymes were identified in this diverse set of non-classified sequences (see Table 2). Interestingly, two main groups arise: (i) post-genomic enzyme characterizations, and (ii) enzymes originating from functional metagenomics-based discovery and characterization. Here, the cytoplasmic *endo*-β-1,6-glucanase Exg3 from the model organism *Schizosaccharomyces pombe*[52] is a representative of the former efforts focused on the identification of fundamental activities. On the other hand, the archael multidomain hyperthermophilic cellulase EBI-244 screened for its ability to degrade CMC at high temperature represents the latter category of efforts [66].

**Table 2:** Characterized carbohydrate-active enzymes of family GH5 not yet classified into subfamilies

**Description**	**EC**	**Assay**	***Organism***	**Accessions**	**Modular structure**	**Taxonomic class**
β-mannanase A (ManA;CelA)	3.2.1.78		*Caldanaerobius polysaccharolyticus KM-THCJ*	AAD09354	GH5 CBM16 CBM16	B-Firmicutes_Clostridia
endo-β-1,4-glucanase D (CelD; CelCCD; EGCCD; Ccel_0840)	3.2.1.4		*Clostridium cellulolyticum H10 [B]*	BAA14354 ACL75216	GH5 CBM11 DOC	B-Firmicutes_Clostridia
endo-β-1,4-glucanase/b-1,3:1,4-glucanase H (CelH)	3.2.1.4 3.2.1.73		*Clostridium thermocellum NCIB 10682*	AAA23225	GH26 GH5 CBM11 DOC	B-Firmicutes_Clostridia
cellulase (EBI-244)	3.2.1.4		*Desulfurococcaceae archaeon EBI-244*	AEB53062	GH5	A-Crenarchaeota
endo-β-1,4-glucanase 3 (Cel3;Cel-3; Eg3; Fisuc_2230; FSU_2772)	3.2.1.4		*Fibrobacter succinogenes subsp. succinogenes S85*	AAA24893 ACX75816 ADL25000	GH5	B-Fibrobacteres_Acidobacteria group
Fisuc_2933/FSU_0196		AGM	*Fibrobacter succinogenes subsp. succinogenes S85*	ACX76513 ADL26912	GH5 CBM4	B-Fibrobacteres_Acidobacteria group
Fisuc_1523/FSU_2005		MUC	*Fibrobacter succinogenes subsp. succinogenes S85*	ACX75120 ADL26743	GH5	B-Fibrobacteres_Acidobacteria group
endoglucanase (CelA; lpg1918)	3.2.1.4	AHEC	*Legionella pneumophila subsp. pneumophila str. Philadelphia 1*	AAU27988	GH5	B-Gammaproteobacteria
endo-β-1,4-glucanase 5B (Sde_2490)	3.2.1.4		*Saccharophagus degradans 2-40*	ABD81750	CBM6 GH5	B-Gammaproteobacteria
endo-β-1,4-glucanase 5E (Sde_2929)	3.2.1.4		*Saccharophagus degradans 2-40*	ABD82186	CBM6 CBM6 GH5	B-Gammaproteobacteria
endo-β-1,6-glucanase (Exg3; SPBC2D10.05)	3.2.1.75		*Schizosaccharomyces pombe 972 h-*	CAA21163 NP_596224	GH5	E-Fungi
endo-β-1,4-glucanase (Cel5G)	3.2.1.4		*uncultured bacterium*	ADD71777	GH5	B-environmental samples
SARM_0034/694713_55880/TW39		LIC CMC	*uncultured organism*	ADX05705	GH5	U-unclassified sequences
SARM_0047/1057205_158590/TW-15		CMC	*uncultured organism*	ADX05718	GH5	U-unclassified sequences
SARM_0086/0_06533/TW-18		PCW	*uncultured organism*	ADX05761	GH5	U-unclassified sequences
β-glucanase (RR.06; RR.06-1; BglC)		BBG Cel5 LIC	*unidentified microorganism*	CAJ19140	GH5	U-unclassified sequences
β-glucanase (RR.10; RR.10-1)		BBG	unidentified microorganism	CAJ19146		U-unclassified sequences

The active enzymes are tagged by their Enzyme Classification (EC) number (see Table 1) or by the significant positively assayed substrates that are: AEHC - AZCL-HE cellulose; AGM - AZCL-galactomannan; BBG – barley β-glucan; Cel5 – cellopentose; CMC - carboxymethyl cellulose; LIC –lichenan; MUC - 4-methylumbelliferyl-β-D-cellobioside; PCW – plant cell wall.

#### Non-catalytic GH5 subfamilies and GH5 modules

Interestingly, the analysis subjacent to the subfamily classification revealed proteins that are likely to be catalytically inactive (or perhaps possess alternate mechanisms and activities to the canonical GHs), due to incomplete catalytic machinery (see Figure 2d). GH5_30 is the only subfamily where all sequences are lacking the essential amino acids for GH activity. In addition, some members of other groups also appear to have lost their catalytic machinery, such as a *Xanthomonas-*specific subgroup that appears to rapidly emerge from subfamily GH5_1 (Additional file 1: Figure S1). All three members of this subgroup present an architecture where the inactive GH5 module is appended to an expansin module and an adjacent CBM63 module at the C-terminus. Although no catalytic chemical activity has been identified for expansin modules, it is significant that the knockout of CelXoB renders *Xanthomonas oryzae pv. oryzae KACC 10331* avirulent [67]. Another emerging non-catalytic GH5 subfamily is closely related to subfamily GH5_8. All three members of this new subgroup are lipoproteins that combine the apparently catalytically inactive GH5 module with a large C-terminal extension. These two subgroups share long branches in the common GH5 tree suggesting rapid evolution. Interestingly, a single member of subfamily GH5_48 has lost the catalytic machinery. Besides this loss, it is however still similar to other members of the subfamily. Whether this is a snapshot of the early steps of a new arising function or a sequencing error it is premature to say. Finally, the only distant GH5 member having lost its catalytic acid–base that has been functionally characterized is the putative carbohydrate biosensor Rsi24C-GH5 from *Clostridium thermocellum ATCC 27405*[68]. Although its catalytic activity was lost, the extracellular GH5 module was shown to interact with crystalline cellulose so that a recognition signal could be conveyed by to its intracellular N-terminal anti-σ factor. The losses of the catalytic machinery here described convey that family GH5 sequences are also subject to recurring evolution that leads to novel functions. This type of evolutionary event has been described previously in other GH families. For instance, amino acid transporters derived from ancestral α-amylases are found in family GH13 [24], inactivated chitinases evolved into xylanase inhibitors in family GH18 [69], and mammalian lactalbumins, which are related to GH22 lysozymes, are all well-known examples of the recent evolution of glycosidases to acquire novel functionalities [70].

## Conclusions

When the first five historical subfamilies of GH5 were established in 1990, the total number of GH5 protein sequences was 21 [16]. More than 20 years later (August 2012), this number has increased approximately 150 times to exceed 3,200. The practical difficulties of handling such large datasets notwithstanding, this abundance of sequences is both a boon and a bane for phylogenetic analysis and functional prediction.

Assigning proteins to a large GH-family, like GH5, which harbors multiple specificities and activities, does not unlock the full potential of sequence-based classification. Thus, one aim of the present investigation was to obtain an improved correlation between protein sequences and catalytic specificity by refining a finer hierarchical level, the subfamily, for GH5 members. Up to 80 percent of the existing GH5 sequences were segregated into 51 subfamilies. Of these subfamilies, a total of 31 contained at least one member characterized to some degree, whereas 20 lacked enzymatically-characterized members altogether. Out of the 31 subfamilies characterized to some extent, 17 were monospecific and eleven were polyspecific (containing two or more enzyme activities). Nonetheless, one activity typically predominates within polyspecific subfamilies. Interestingly, both *endo*- and *exo*-acting enzymes have been observed in the same subfamily, e.g. GH5_1, GH5_7 and GH5_9, illustrating that (if real) the two types of activities reflect details of the three-dimensional structures. As a consequence of the canonical double displacement mechanism employed by GH5 enzymes, which involves the formation of a covalent glycosyl-enzyme intermediate, GH5 members can potentially catalyze transglycosylation in addition to, or instead of, hydrolysis [71]. Although the amount of biochemical data is presently limited, we observed that subfamily classification in GH5 does not appear to correlate with transglycosylation activity, thus indicating that this property is also a consequence of subtle protein structural details.

This effort provides a first comprehensive view of the coverage and distribution of the curated set of 400 experimentally characterized enzymes in the GH5 family and couples this information with an extensively updated sequence-based GH5 subfamily division. In particular, it provides insights into the evolution of GH5 proteins, and the classification results can be used to assist in candidate protein selection for enzyme discovery and bioprospecting projects. For instance, both the members from the twenty defined subfamilies lacking functional characterization, as well as the numerous phylogenetic outliers, provide a vast number of interesting targets for future studies. In particular, although a significant amount of tertiary structural data is already available for GH5, the present work highlights that a large number subfamilies would benefit from a 3-D structure for at least one subfamily member. Moreover, the data presented here, and available at the CAZy database [11] as a community resource, will serve as a guide for protein engineering approaches exploiting the diverse activities found within the GH5 family.

Finally, in the present climate in which sequence data is literally flooding public databases, incorrect protein function annotations are too easily propagated by automated computer-based prediction methods, thereby jeopardizing the usefulness of these annotations. Increasingly rapid sequence accumulation is worsening the scenario. This problem is particularly illustrated by the GH5_11 subfamily of plant and fungal proteins that are annotated as cellulases in public databases, including the widely-used Arabidopsis Information Resource [72], despite a complete lack of experimental support for any one of its members. Such excesses of over-annotation equally affect the presumed non-catalytically active proteins and subfamilies. For example, subfamily GH5_30 further exemplifies the pitfalls of automated (mis-)annotation: several members are publicly annotated as mannanases, although they lack the conserved catalytic machinery of the family. To avoid such error propagation, we strongly advocate designating all predicted enzymes as “GH5_*n*” (where *n* is the subfamily number) until an activity has been rigorously demonstrated by biochemical experimentation.

The GH5 subfamily classification presented here provides a framework to sort family members into meaningful, predictive categories. By taking a conservative approach to protein annotation, this method offers a rigorous strategy to avoid misleading functional prediction in large-scale genomic sequencing projects. Whilst the subfamilies described herein generally act on a single substrate (seventeen monospecific subfamilies were identified), it is important to stress that precise details of glycoside hydrolase function, such as the extent of *endo*- vs. *exo*- modes of cleavage or the transglycosylation-to-hydrolysis ratio is unlikely to be predictable from sequence alone. We therefore recommend that such over-reaching predictions be altogether abandoned in genomic sequence annotation. To aid and advance global efforts in *de novo* sequence annotation, the GH5 subfamily classification scheme is now publicly available at the CAZy database [11].

## Methods

The GH5 subfamilies were defined using the methods described for the subfamily classification of GH13 and all PL families [24,25], which are briefly summarized here. After an initial removal of obviously incomplete and/or erroneous sequences, a total of 2347 full length GH5 catalytic module sequences were retrieved from the CAZy database (October 2011) and subdivided into two sets. One set of 414 sequences contained all GH5 modules from biochemically characterized and sequences positively tested in activity tests against a variety of substrates. The second set was composed of 1957 non-characterized sequences The latter subset was clustered at 75% identity using UCLUST4.0, a part of the USEARCH 4.0 package [73], and was reduced to 971 sequences. When combined with the 414 GH5 module sequences from a biochemically characterized and positively assayed subset we obtained a total of 1385 sequences. These sequences were aligned using MUSCLE 3.7 [74] in two steps. An initial alignment was performed and its quality visually inspected so that the remaining incomplete and problem sequences were identified and edited or removed. This procedure ensured that the GH5 module boundaries were clear and that the sequences were trimmed if required, and that a majority of the residues constituting the catalytic site was present or that the alignment was not ambiguous. A final set of 1367 remaining sequences were then realigned using the same procedure. The eliminated, often fragmentary, sequences were used to complement biochemical activity information when relevant at later stages.

The resulting multisequence alignment of family GH5 catalytic domain sequences was used to infer an approximate-maximum-likelihood phylogenetic tree with FASTTREE 2.1 [75], a program adapted to the analysis of large sequence sets, using the Whelan Goldman model of amino acid evolution, the gamma option to rescale the branch lengths and compute a Gamma20-based likelihood, a total of four rounds of minimum-evolution moves, and options to make the maximum-likelihood nearest-neighbor interchanges more exhaustive. The identification and tagging of subfamilies followed a multi-step procedure. First, the tree was analyzed to tag the different sequences and nodes corresponding to the first 10 “historical” subfamilies (A1 to A10, described in the Introduction). These steps were performed to ensure continuity in subfamily definitions. For the remaining sequences in the tree, distinct nodes corresponding potential subfamilies were visually identified and their consistency checked using a procedure similar to that described [24]. Within each of these groups, different starting sequences were selected and manual BLAST2 queries [76] performed against all the sequences found in CAZy in order to identify self-contained ensembles and establish subfamily limits. This analysis ensured that the each subfamily that was retained was singular and that the removal of sequences by the initial UCLUST filtering procedure and of fragmentary sequences did not introduce any bias. Finally, only subfamilies containing at least five sequences from different organisms were considered.

Subsequently, for each defined subfamily, maximum likelihood (ML) phylogenetic trees were built by using PhyML [77], and the reliability of the inferred relationships the trees was tested by bootstrap analysis using 100 resamplings of the data set.

## Endnotes

^a^No EC number is presently available for β-1,3-(gluco)mannanase activity.

^b^No EC number is presently available for arabinoxylanase activity.

## Competing interests

The authors declare they have no competing interests.

## Authors’ contributions

Pedro M. Coutinho performed the global phylogenetic analysis; Henrik Aspeborg and Yang Wang performed the phylogenetic analyses of each subfamily; Bernard Henrissat and Pedro M. Coutinho collected and curated all GH5 sequences used in the present analysis; Harry Brumer and Bernard Henrissat designed research and analyzed the data. The paper was collectively written. All authors read and approved the final manuscript.

## Supplementary Material

Additional file 1 **Figure S1.** Rectangular phylogram view of the phylogenetic tree of family GH5. Branches corresponding to subfamilies 1–53 are shown in color and the individual subfamilies have their corresponding subfamily numbers as indicated in Figure 1. The branches corresponding to sequences not included into subfamilies are in black. Each individual protein module node is identified by a varying number of fields separated by “|” indicating: (i) the organism, with 3 letters for the genre and either 5 letters for the species or full strain code; (ii) the protein accession in public databases, typically GenBank; (iii) if attributed, the subfamily number or other information; (iv) if available, EC numbers (node in bold) or a “*” (node in bold and italic) to indicate precise enzyme characterizations or a simple activity tests, respectively. A suffix like “_2” may indicate the module position if more than one GH5 module is present on peptide. Lower confidence nodes with a SH-like local support below 0.7 (varying from low 0 to strong 1) are indicated with a black dot. Identified sequences without complete catalytic machinery are in red. Individual subfamily trees are also included in this file. Click here for file
