# Supplementary material for: Evolution, substrate specificity and subfamily classification of glycoside hydrolase family 5 (GH5)
Source: BMC Evol Biol. 2012 Sep 20;12:186. doi: 10.1186/1471-2148-12-186 (PMC3526467; doi:10.1186/1471-2148-12-186)
Supplement: Additional file 1 — Figure S1. Rectangular phylogram view of the phylogenetic tree of family GH5. Branches corresponding to subfamilies 1–53 are shown in color and the individual subfamilies have their corresponding subfamily numbers as indicated in Figure 1. The branches corresponding to sequences not included into subfamilies are in black. Each individual protein module node is identified by a varying number of fields separated by “|” indicating: (i) the organism, with 3 letters for the genre and either 5 letters for the species or full strain code; (ii) the protein accession in public databases, typically GenBank; (iii) if attributed, the subfamily number or other information; (iv) if available, EC numbers (node in bold) or a “*” (node in bold and italic) to indicate precise enzyme characterizations or a simple activity tests, respectively. A suffix like “_2” may indicate the module position if more than one GH5 module is present on peptide. Lower confidence nodes with a SH-like local support below 0.7 (varying from low 0 to strong 1) are indicated with a black dot. Identified sequences without complete catalytic machinery are in red. Individual subfamily trees are also included in this file. [file 1471-2148-12-186-S1.pdf]

Legend to Supplementary Figure 1. – Rectangular phylogram view of the phylogenetic tree of family GH5. Branches corresponding to subfamilies 1–53 are shown in color and the individual subfamilies have their corresponding subfamily numbers as indicated in Fig. 1. The branches corresponding to sequences not included into subfamilies are in black. Each individual protein module node is identified by a varying number of fields separated by "|" indicating: (i) the organism, with 3 letters for the genre and either 5 letters for the species or full strain code; (ii) the protein accession in public databases, typically GenBank; (iii) if attributed, the subfamily number or other information; (iv) if available, EC numbers (node in bold) or a "*" (node in bold and italic) to indicate precise enzyme characterizations or a simple activity tests, respectively. A suffix like "_2" may indicate the module position if more than one GH5 module is present on peptide. Lower confidence nodes with a SH-like local support below 0.7 (varying from low 0 to strong 1) are indicated with a black dot. Identified sequences without complete catalytic machinery are in red.

Supplementary Fig S1

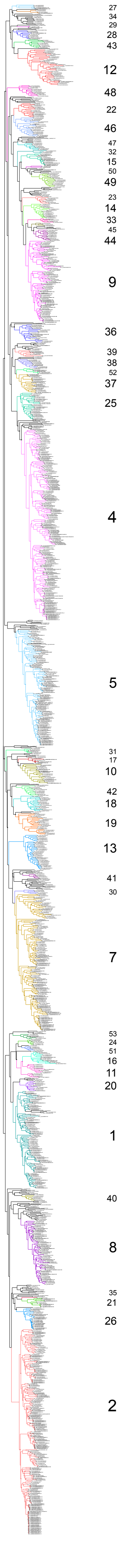

Figure GH5_1

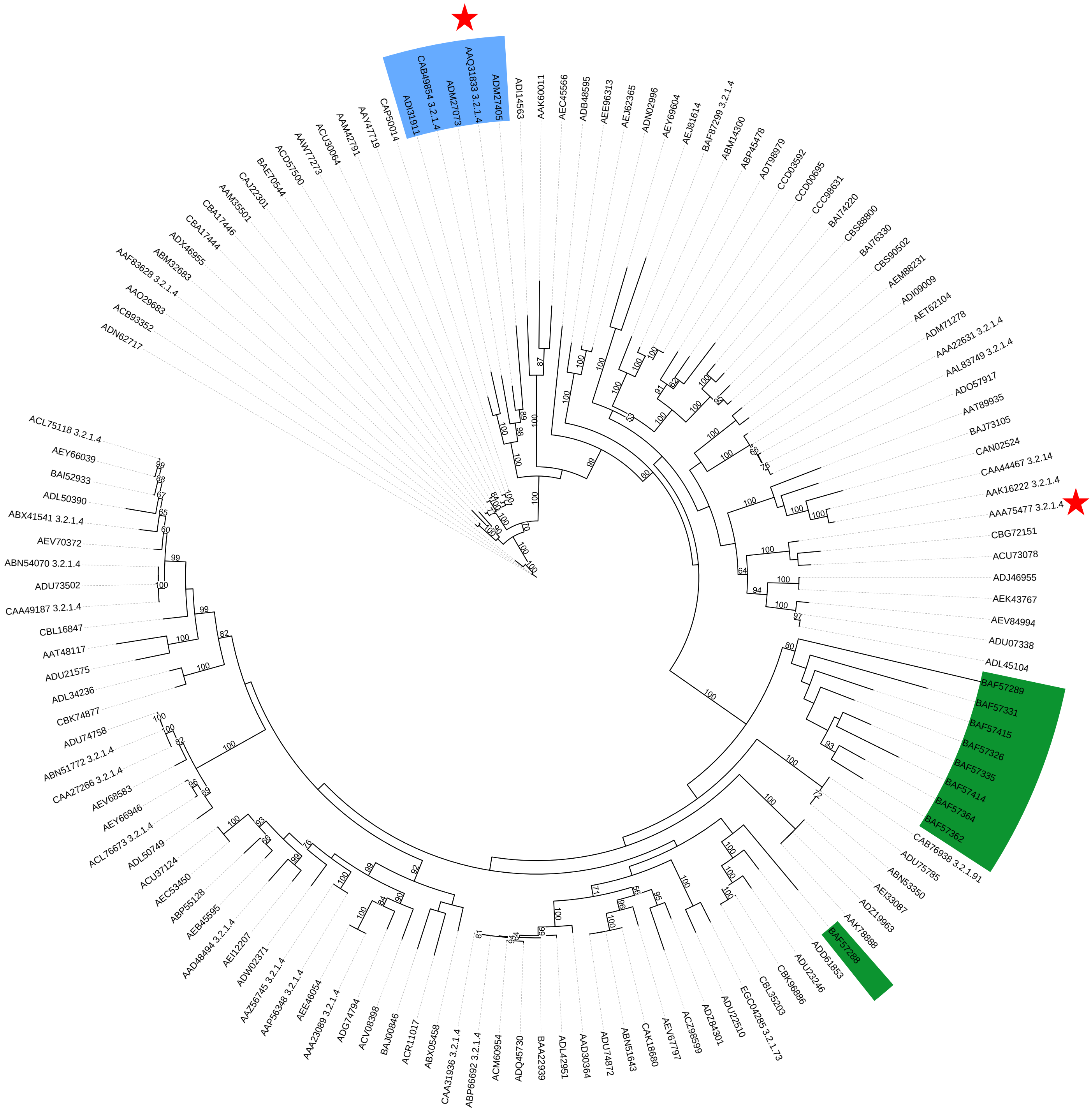

Figure GH5_1:

Phylogenetic tree on subfamily GH5_1 based on maximum likelihood with 100 bootstrap replications. Only the GH5 catalytic domain is used for the phylogenetic analysis. Bootstrap values > 50 are displayed. The green range marks protist sequences, whereas the blue range marks sequences from Archaea. Bacterial sequences are displayed without color. Enzymes with a solved 3-D structure are marked with a red star.

Figure GH5_2

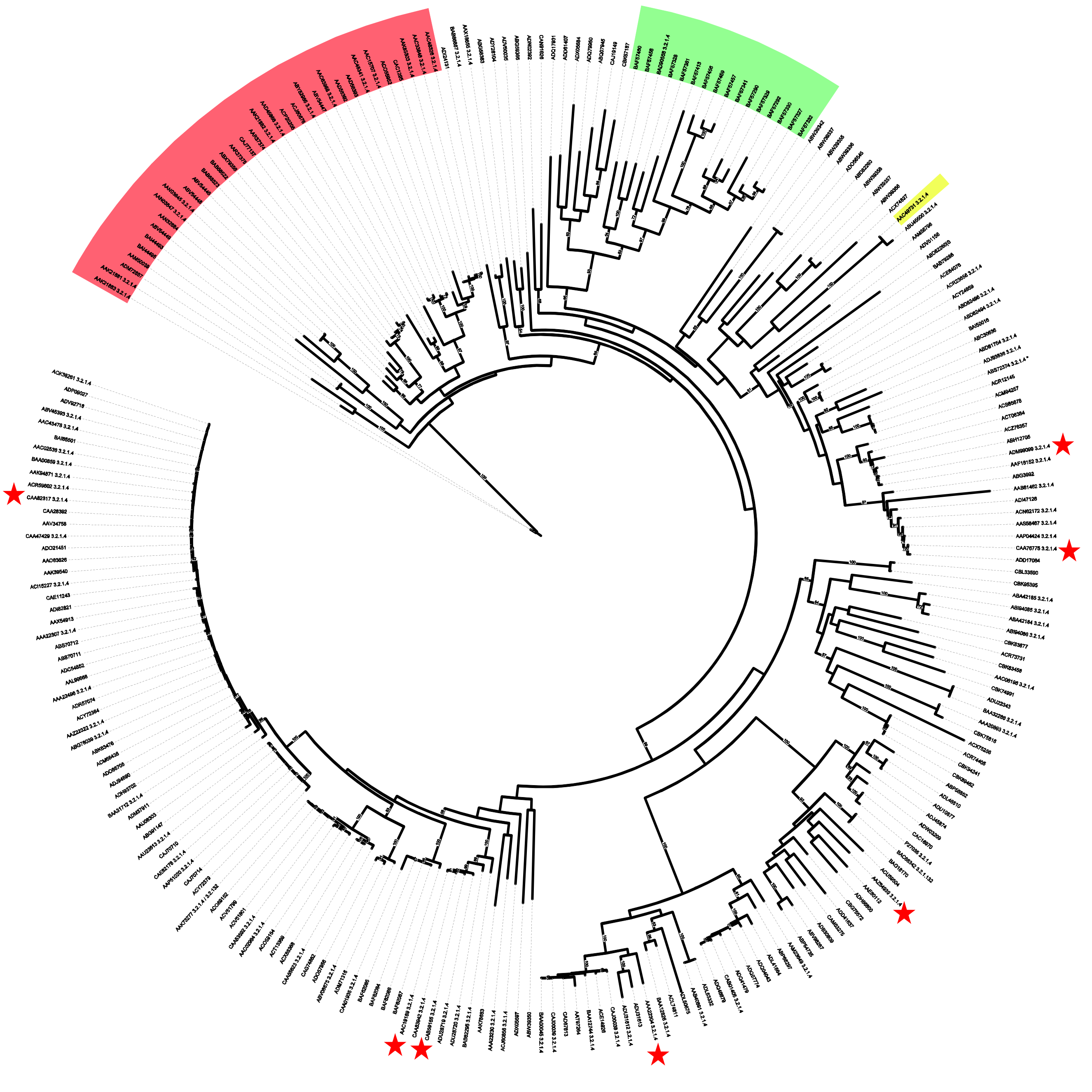

Figure GH5_2:

Phylogenetic tree on subfamily GH5_2 based on maximum likelihood with 100 bootstrap replications. Only the GH5 catalytic domain is used for the phylogenetic analysis. Bootstrap values > 50 are displayed. The green range marks protist sequences, whereas the red range marks sequences from Metazoa and yellow marks a fungal enzyme. Bacterial sequences are displayed without color. Enzymes with a solved 3-D structure are marked with a red star.

Figure GH5_4

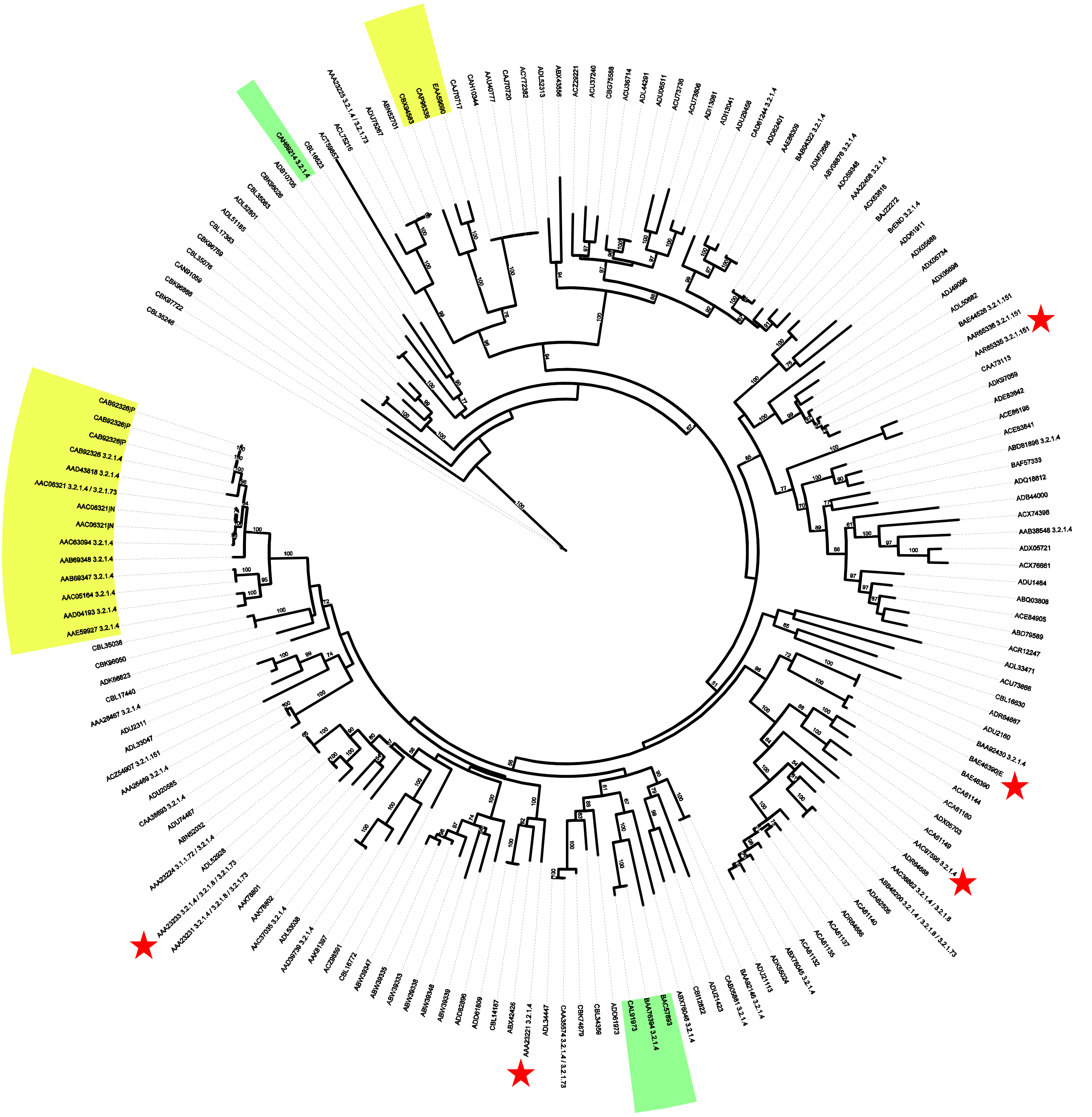

Figure GH5_4:

Phylogenetic tree on subfamily GH5_4 based on maximum likelihood with 100 bootstrap replications. Only the GH5 catalytic domain is used for the phylogenetic analysis. Bootstrap values > 50 are displayed. The green range marks Alveolata sequences, whereas the yellow range marks fungal sequences. Bacterial sequences are displayed without color. Enzymes with a solved 3-D structure are marked with a red star.

Figure GH5_5

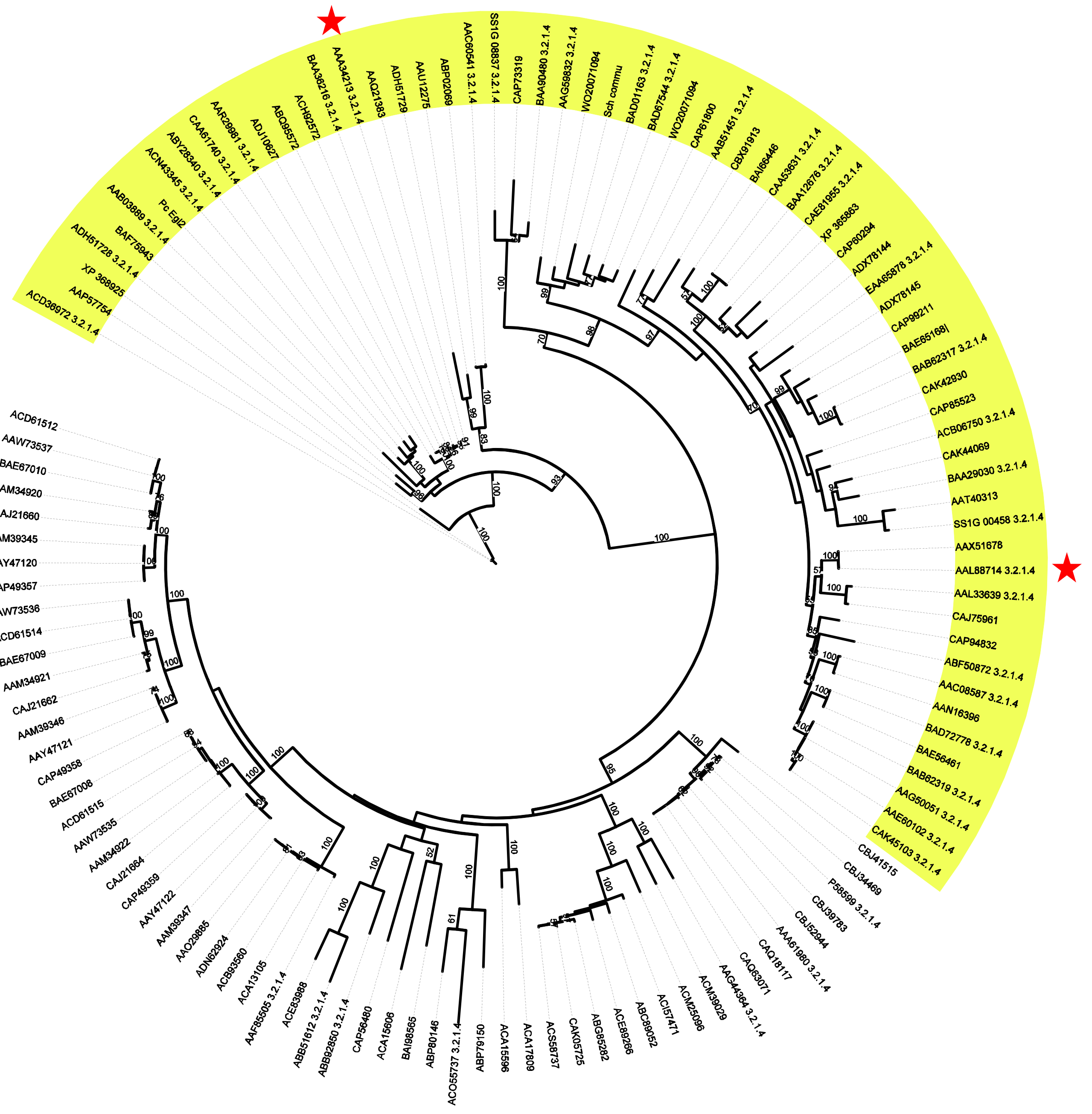

Figure GH5_5:

Phylogenetic tree on subfamily GH5_5 based on maximum likelihood with 100 bootstrap replications. Only the GH5 catalytic domain is used for the phylogenetic analysis. Bootstrap values > 50 are displayed. The yellow range marks fungal sequences. Bacterial sequences are displayed without color. Enzymes with a solved 3-D structure are marked with a red star.

Figure GH5_7

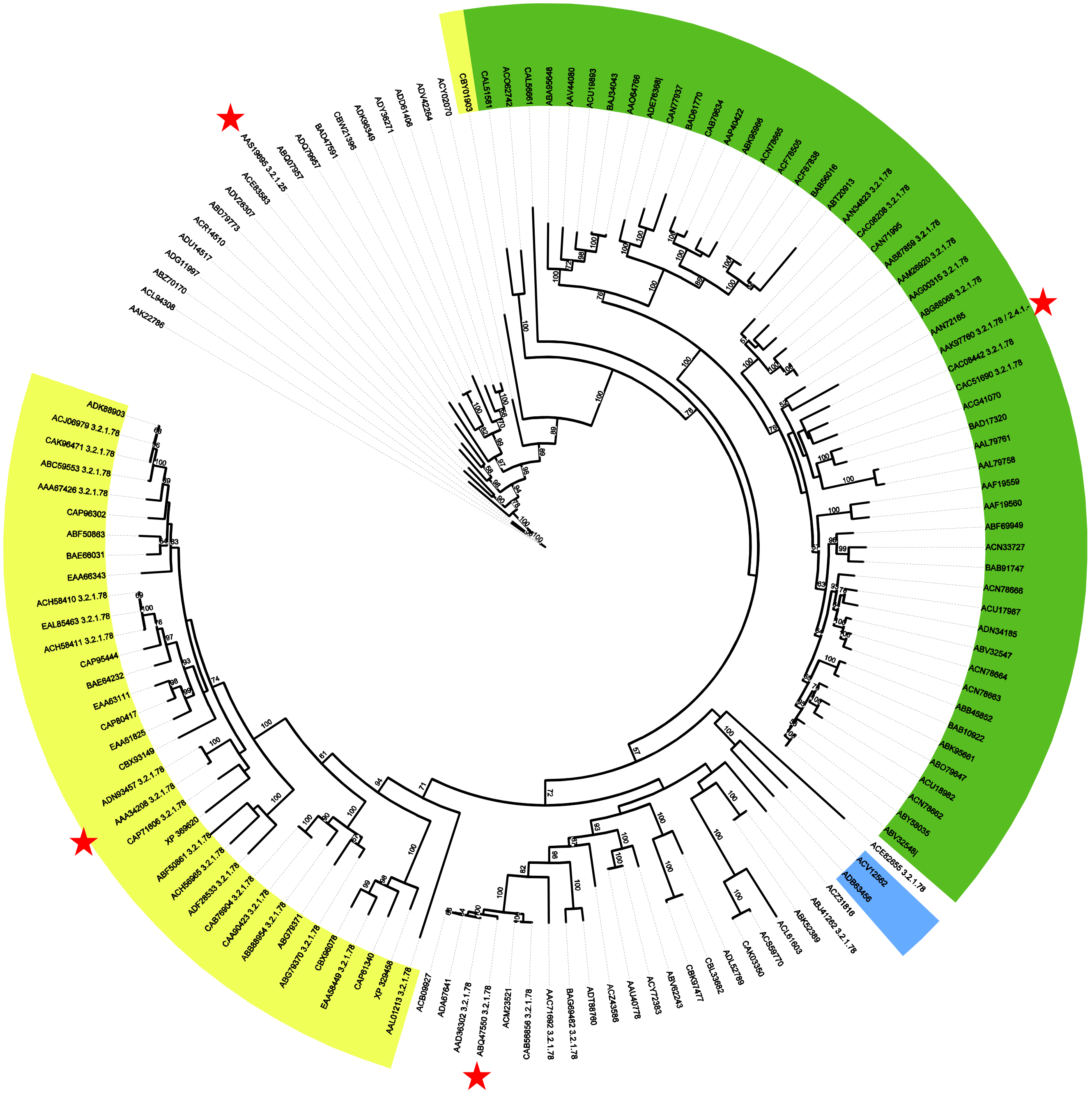

Figure GH5_7:

Phylogenetic tree on subfamily GH5_7 based on maximum likelihood with 100 bootstrap replications. Only the GH5 catalytic domain is used for the phylogenetic analysis. Bootstrap values > 50 are displayed. The green range marks plant sequences, whereas the blue range marks sequences from Archea and yellow marks fungal enzymes. Bacterial sequences are displayed without color. Enzymes with a solved 3-D structure are marked with a red star.

Figure GH5_8

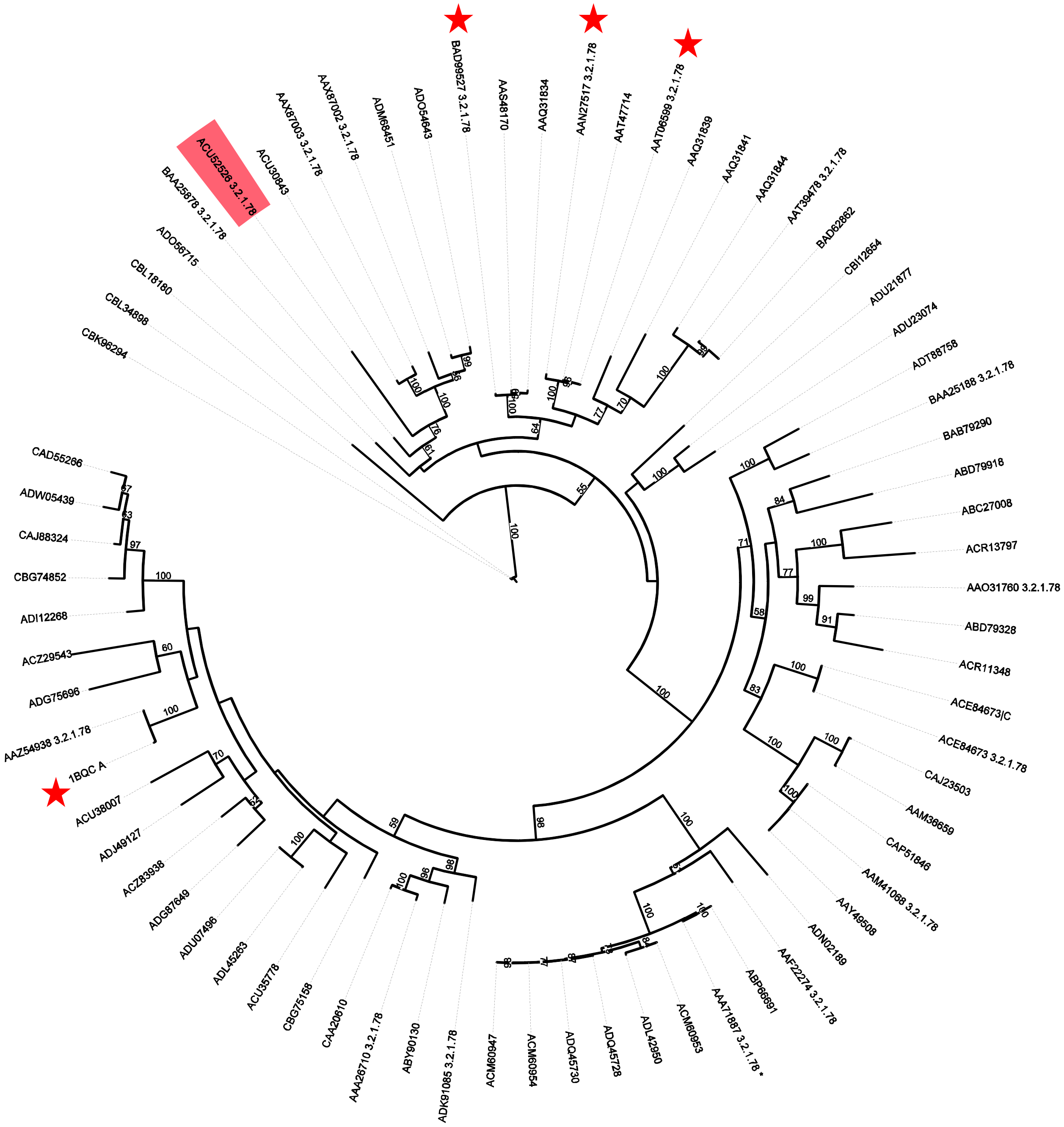

Figure GH5_8:

Phylogenetic tree on subfamily GH5_8 based on maximum likelihood with 100 bootstrap replications. Only the GH5 catalytic domain is used for the phylogenetic analysis. Bootstrap values > 50 are displayed. The red range marks a sequence belonging to the Metazoa kingdom. Bacterial sequences are displayed without color. Enzymes with a solved 3-D structure are marked with a red star.

Figure GH5_9

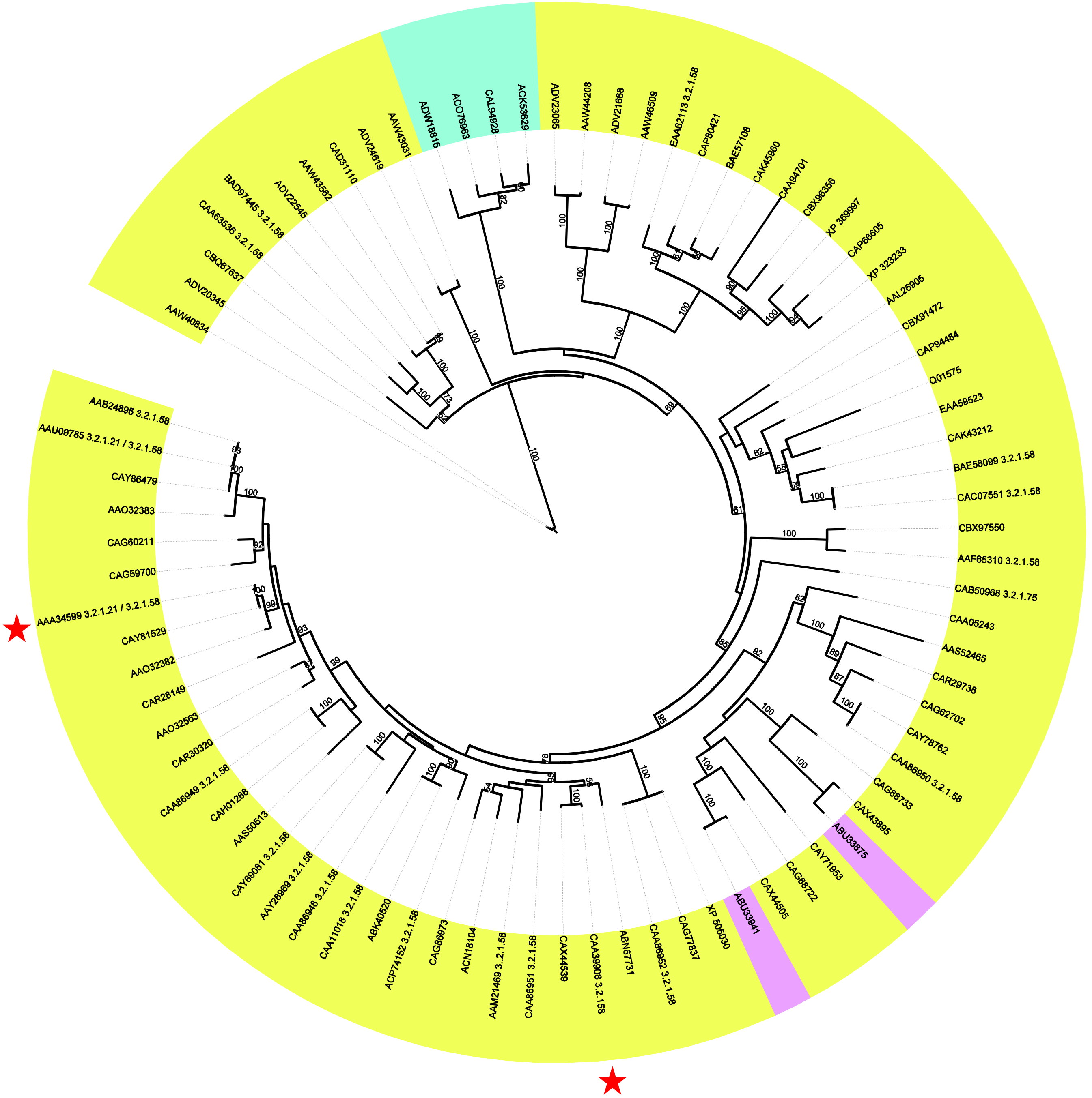

Figure GH5_9:

Phylogenetic tree on subfamily GH5_9 based on maximum likelihood with 100 bootstrap replications. Only the GH5 catalytic domain is used for the phylogenetic analysis. Bootstrap values > 50 are displayed. The blue-green range marks bacterial sequences, whereas the purple range marks sequences from Uncultivated organisms and yellow marks fungal enzymes. Enzymes with a solved 3-D structure are marked with a red star.

Figure GH5_10

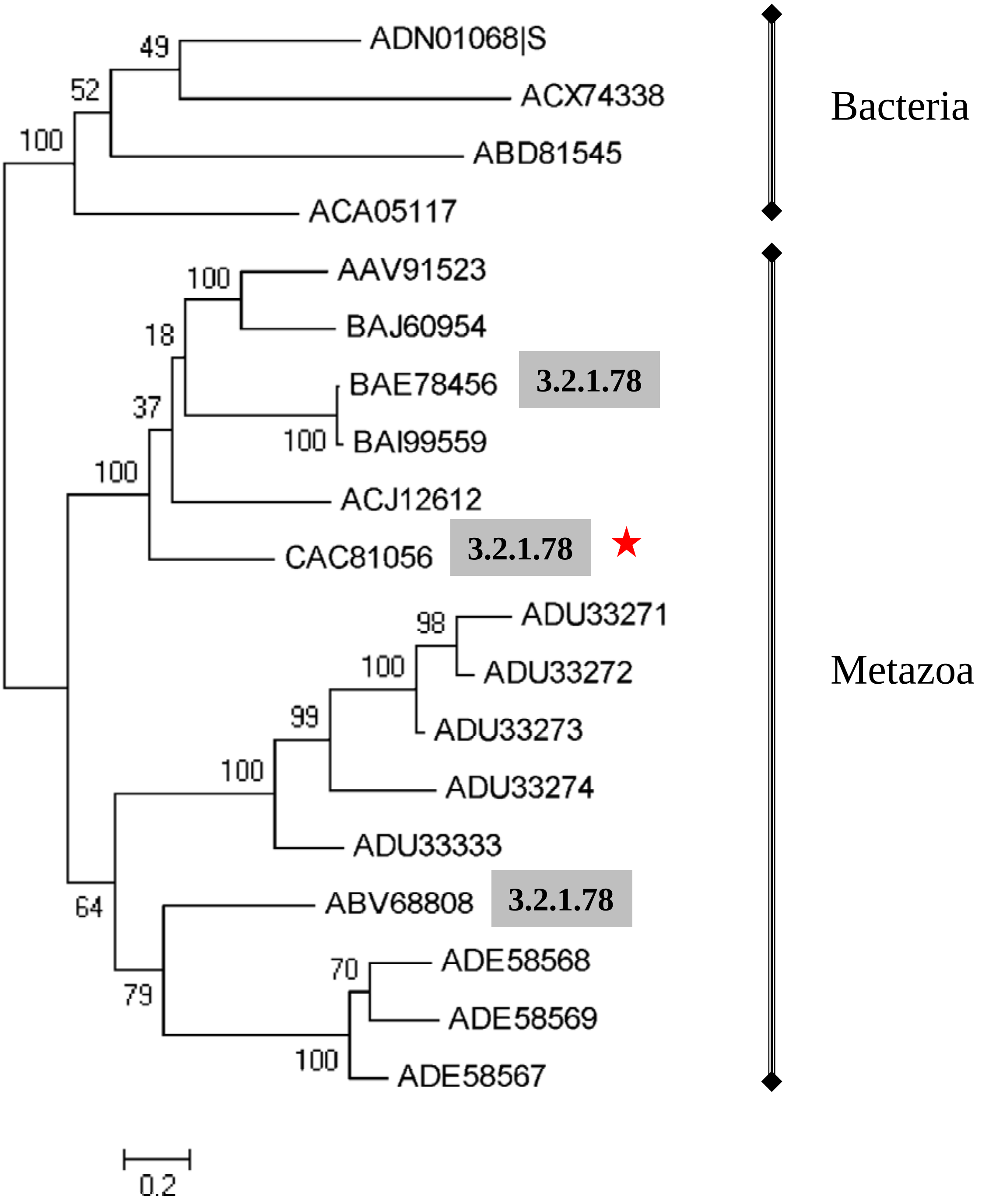

Figure GH5_10:

Phylogenetic tree on subfamily GH5_10 based on maximum likelihood with 100 bootstrap replications. Only the GH5 catalytic domain is used for the phylogenetic analysis. All bootstrap values are displayed. The only enzyme with a solved 3-D structure is marked with a red star.

Figure GH5_11

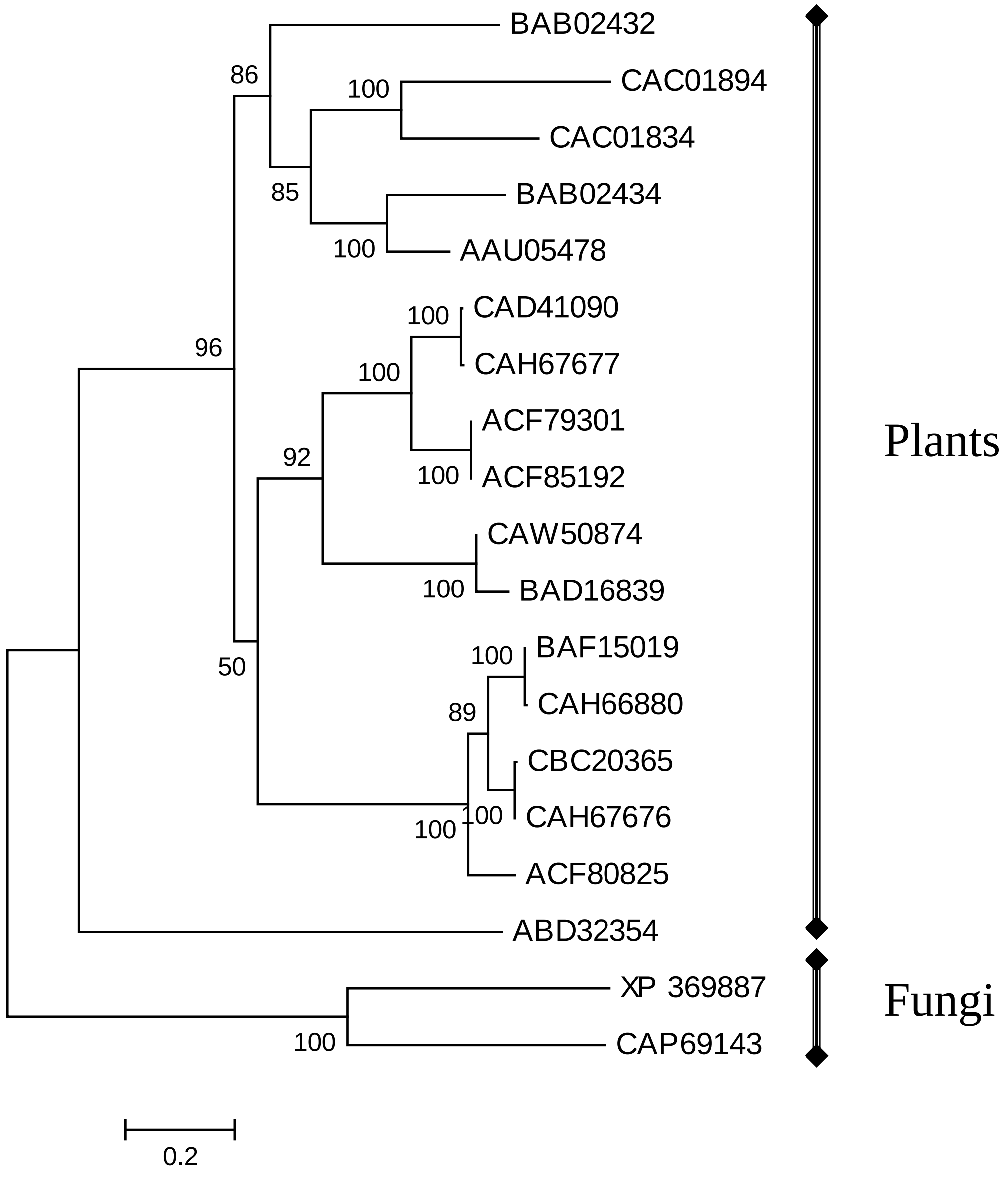

Figure GH5_11:

Phylogenetic tree on subfamily GH5_11 based on maximum likelihood with 100 bootstrap replications. Only the GH5 catalytic domain is used for the phylogenetic analysis. All bootstrap values are displayed.

Figure GH5_12

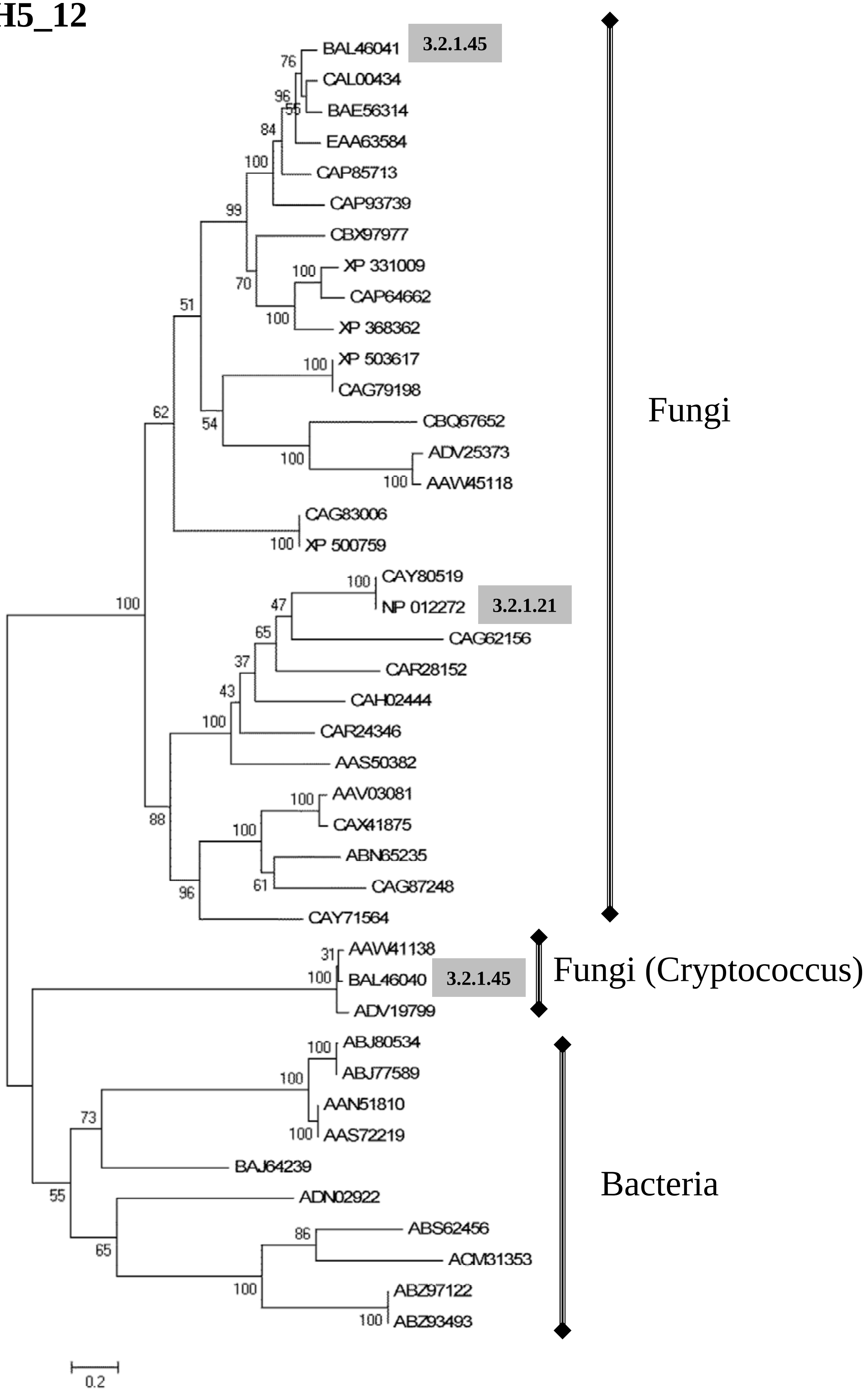

Figure GH5_12:

Phylogenetic tree on subfamily GH5_12 based on maximum likelihood with 100 bootstrap replications. Only the GH5 catalytic domain is used for the phylogenetic analysis. All bootstrap values are displayed.

Figure GH5_13

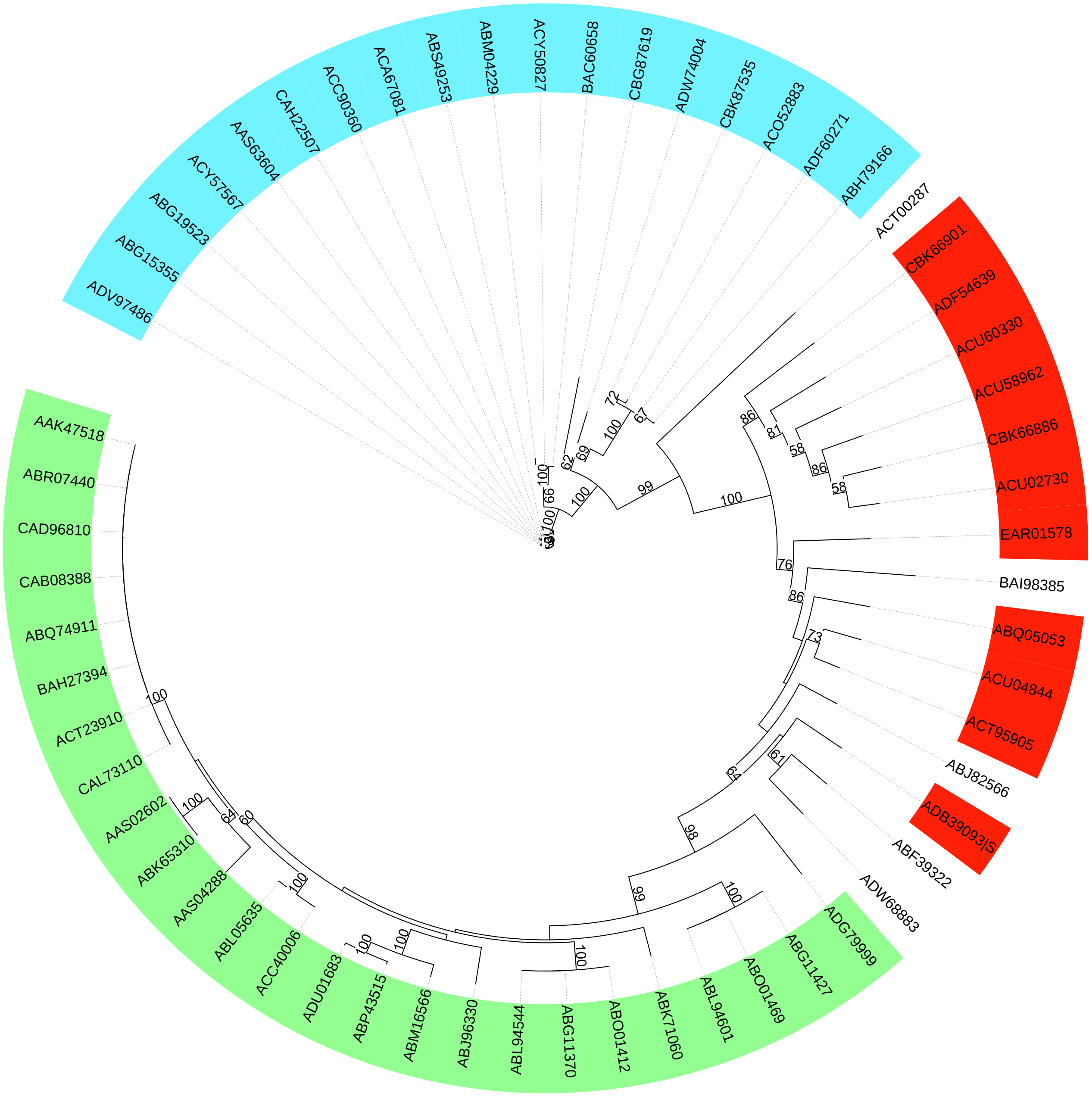

Figure GH5_13:

Phylogenetic tree on subfamily GH5_13 based on maximum likelihood with 100 bootstrap replications. Only the GH5 catalytic domain is used for the phylogenetic analysis. Bootstrap values > 50 are displayed. The green range marks sequences from Actinobacteria, whereas the red range marks sequences from Bacteroidetes, and the blue range marks sequences from Gammaproteobacteria. Bacterial sequences from other classes are displayed without color.

Figure GH5_14

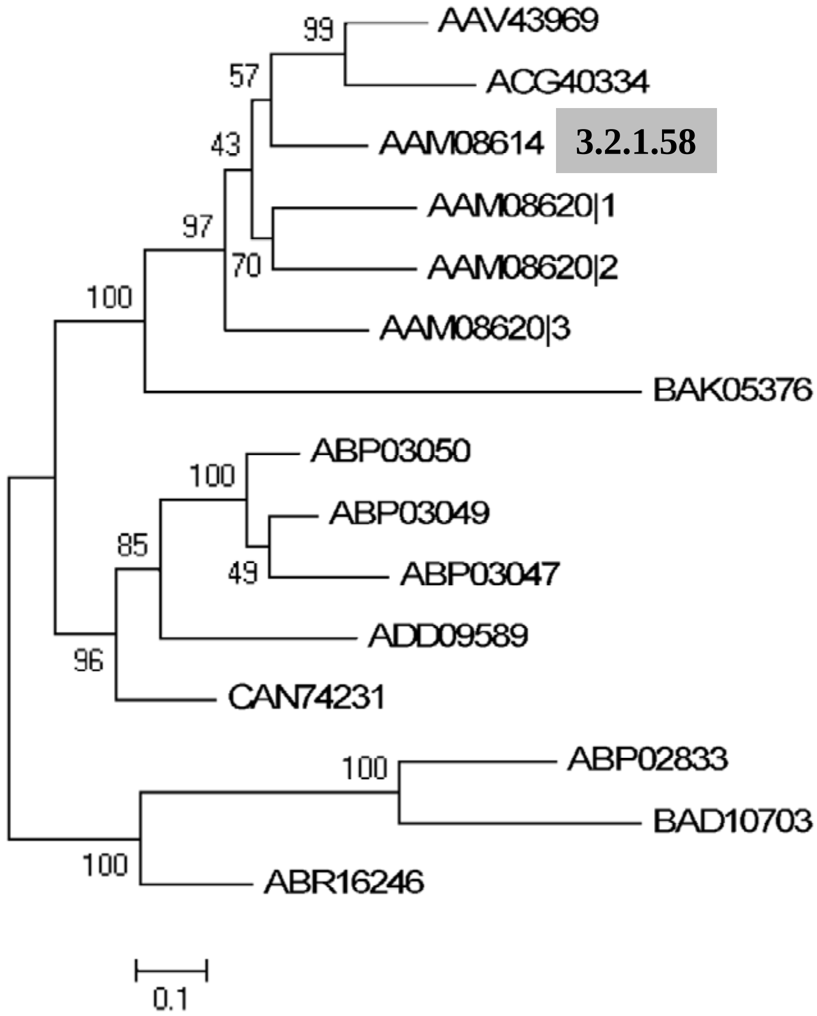

Figure GH5_14:

Phylogenetic tree on subfamily GH5_14 based on maximum likelihood with 100 bootstrap replications. Only the GH5 catalytic domain is used for the phylogenetic analysis. All bootstrap values are displayed.

Figure GH5_15

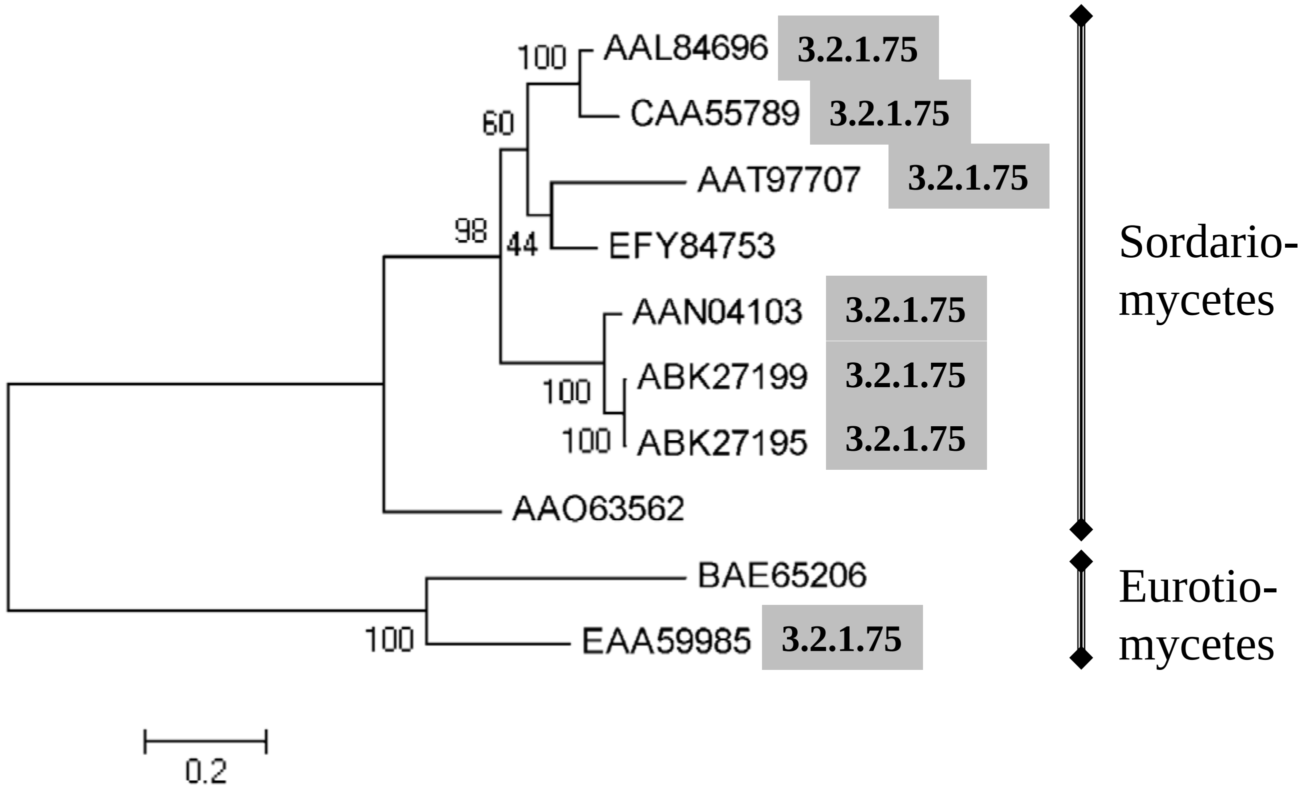

Figure GH5_15:

Phylogenetic tree on subfamily GH5_15 based on maximum likelihood with 100 bootstrap replications. Only the GH5 catalytic domain is used for the phylogenetic analysis. All bootstrap values are displayed.

Figure GH5_16

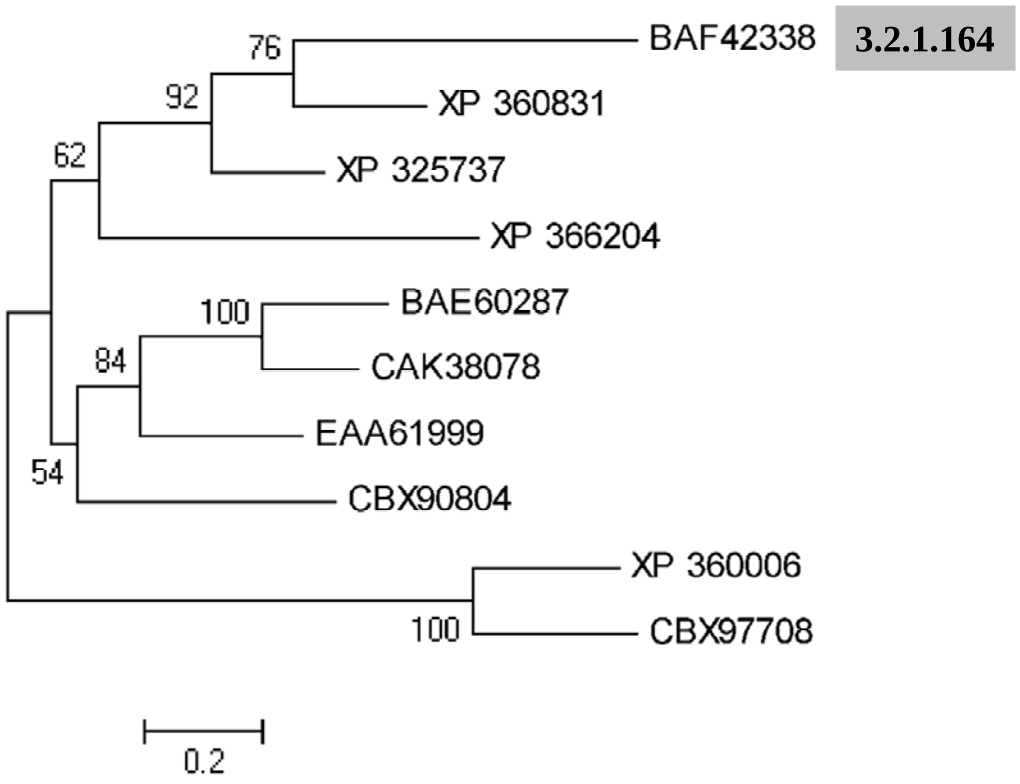

Figure GH5_16:

Phylogenetic tree on subfamily GH5_16 based on maximum likelihood with 100 bootstrap replications. Only the GH5 catalytic domain is used for the phylogenetic analysis. All bootstrap values are displayed.

Figure GH5_17

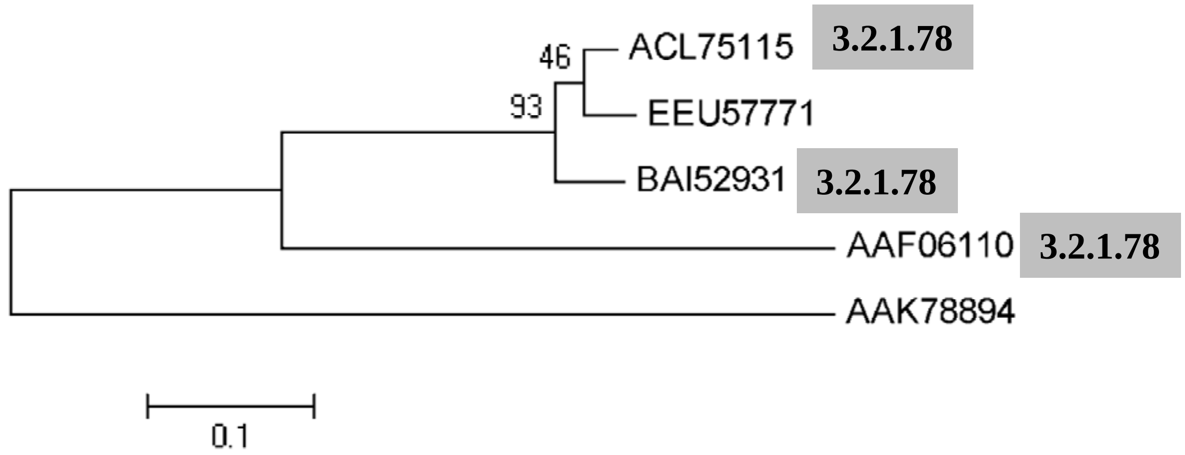

Figure GH5_17:

Phylogenetic tree on subfamily GH5_17 based on maximum likelihood with 100 bootstrap replications. Only the GH5 catalytic domain is used for the phylogenetic analysis. All bootstrap values are displayed.

Figure GH5_18

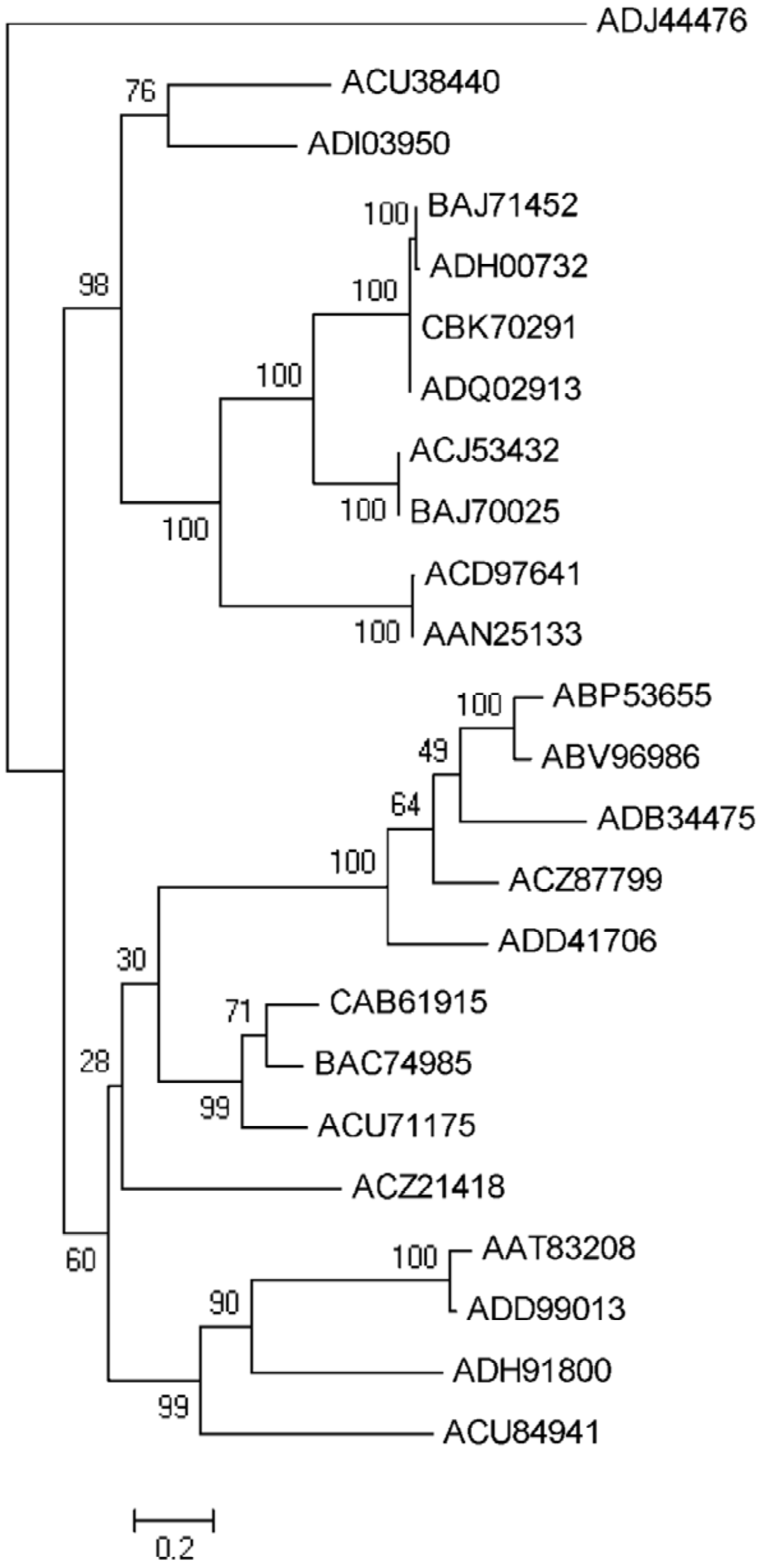

Figure GH5_18:

Phylogenetic tree on subfamily GH5_18 based on maximum likelihood with 100 bootstrap replications. Only the GH5 catalytic domain is used for the phylogenetic analysis. All bootstrap values are displayed.

Figure GH5_19

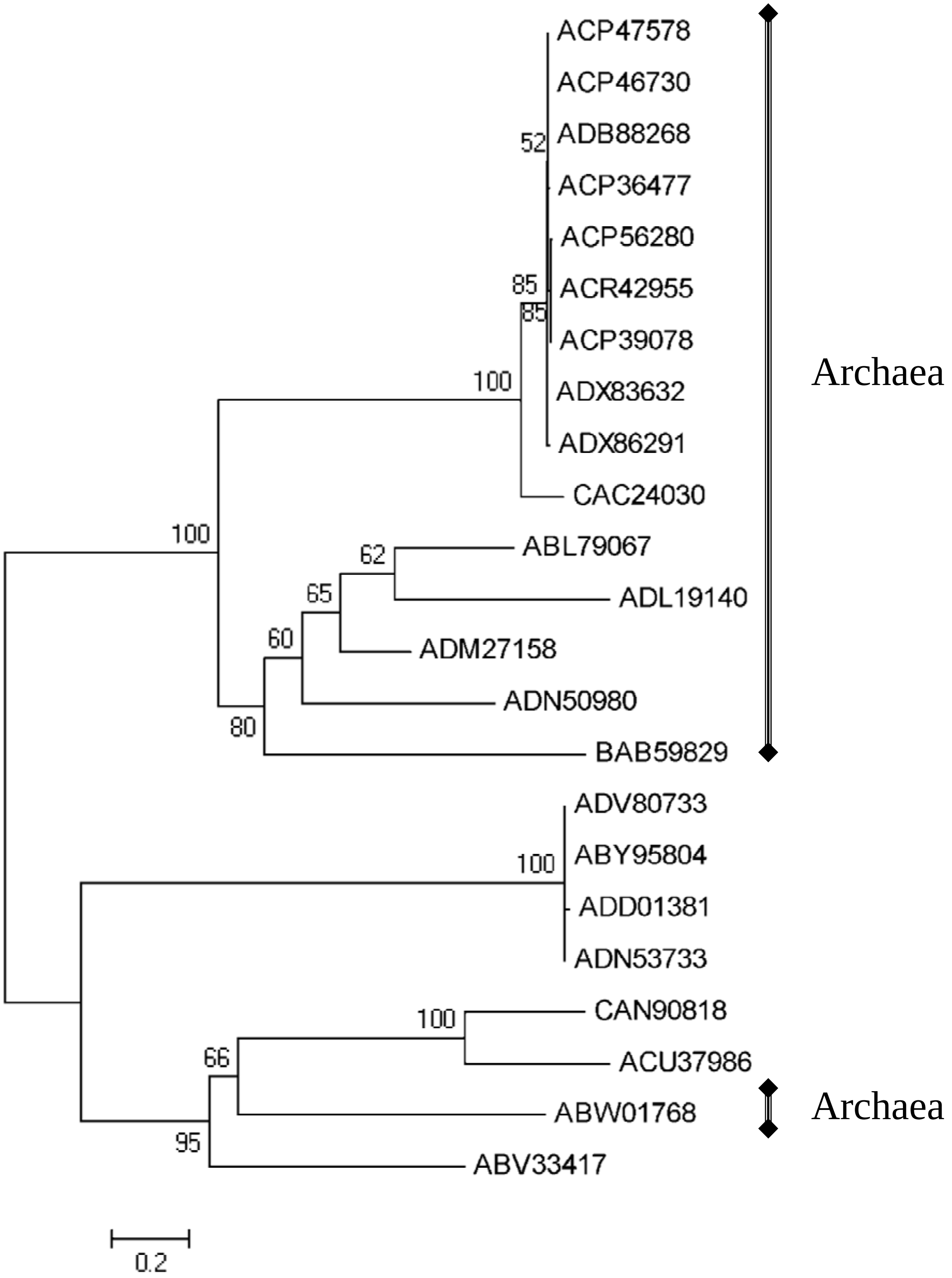

Figure GH5_19:

Phylogenetic tree on subfamily GH5_19 based on maximum likelihood with 100 bootstrap replications. Only the GH5 catalytic domain is used for the phylogenetic analysis. All bootstrap values are displayed.

Figure GH5_20

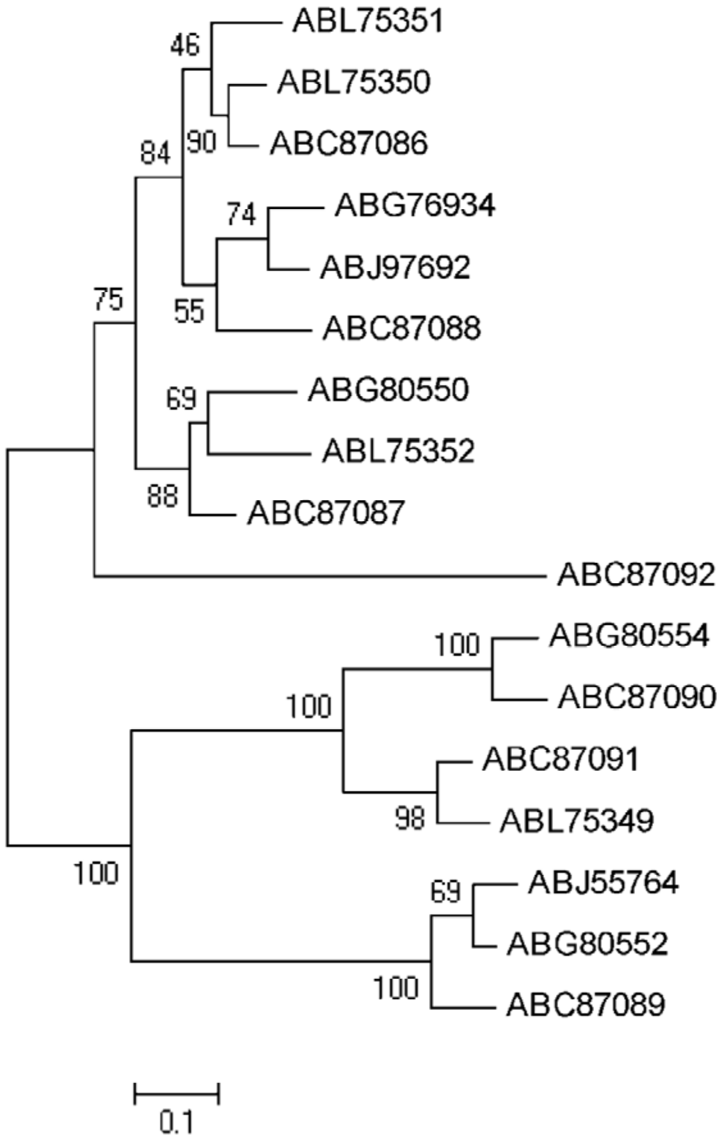

Figure GH5_20:

Phylogenetic tree on subfamily GH5_20 based on maximum likelihood with 100 bootstrap replications. Only the GH5 catalytic domain is used for the phylogenetic analysis. All bootstrap values are displayed.

Figure GH5_21

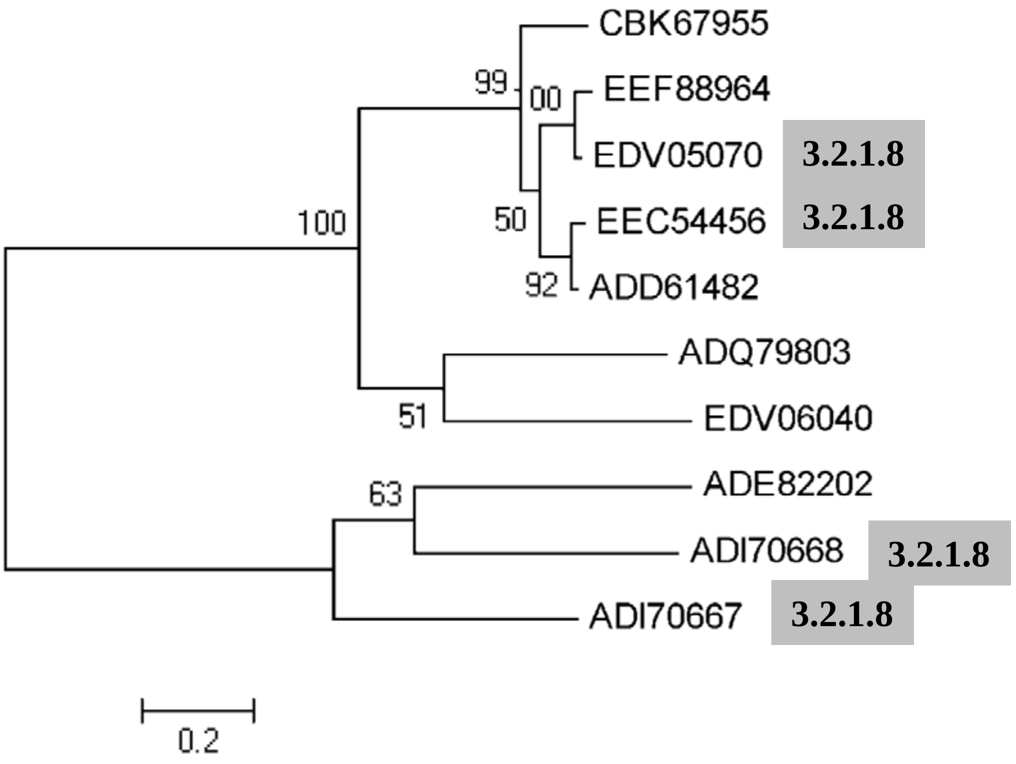

Figure GH5_21:

Phylogenetic tree on subfamily GH5_21 based on maximum likelihood with 100 bootstrap replications. Only the GH5 catalytic domain is used for the phylogenetic analysis. All bootstrap values are displayed.

Figure GH5_22

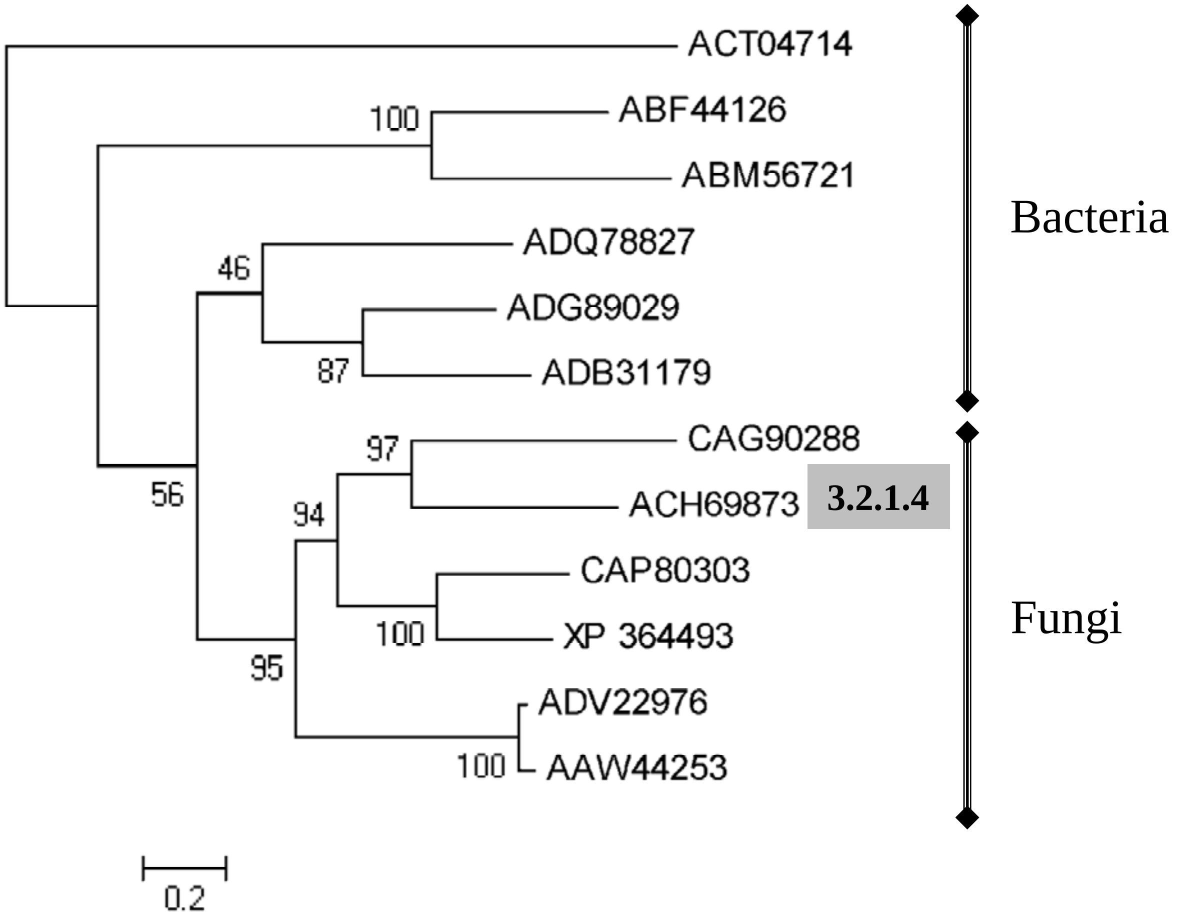

Figure GH5_22:

Phylogenetic tree on subfamily GH5_22 based on maximum likelihood with 100 bootstrap replications. Only the GH5 catalytic domain is used for the phylogenetic analysis. All bootstrap values are displayed.

Figure GH5_23

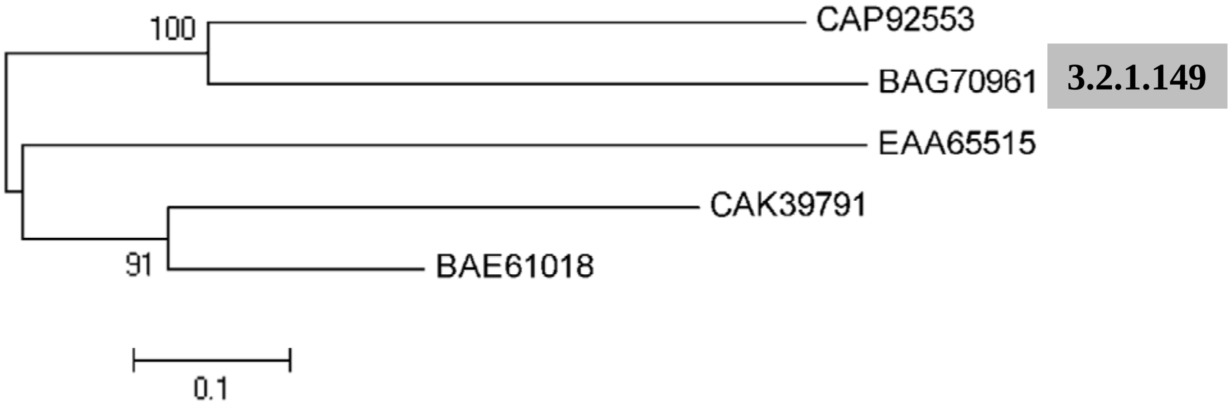

Figure GH5_23:

Phylogenetic tree on subfamily GH5_23 based on maximum likelihood with 100 bootstrap replications. Only the GH5 catalytic domain is used for the phylogenetic analysis. All bootstrap values are displayed.

Figure GH5_24

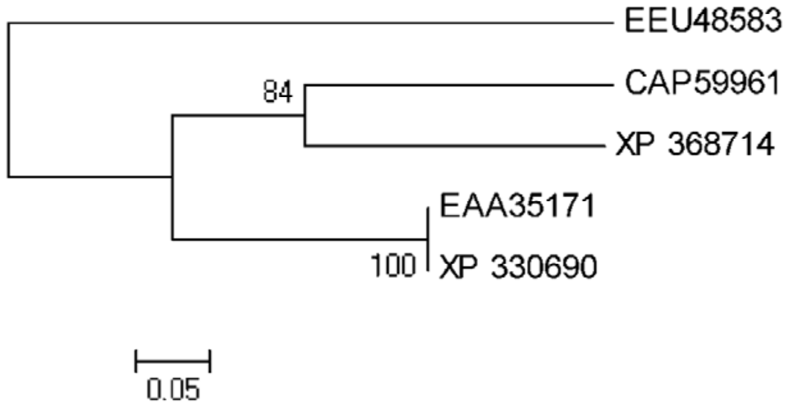

Figure GH5_24:

Phylogenetic tree on subfamily GH5_24 based on maximum likelihood with 100 bootstrap replications. Only the GH5 catalytic domain is used for the phylogenetic analysis. All bootstrap values are displayed.

Figure GH5_25

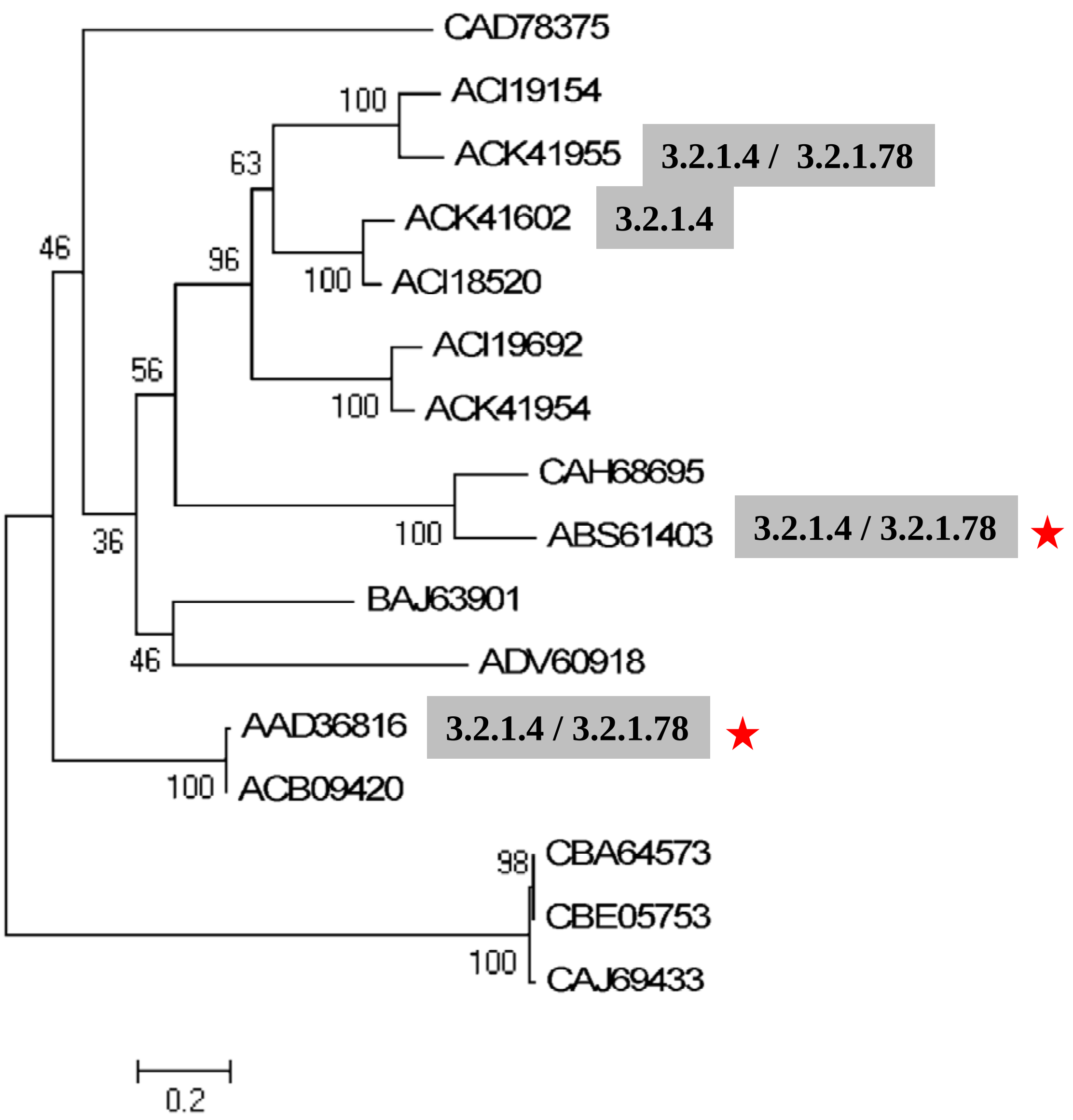

Figure GH5_25:

Phylogenetic tree on subfamily GH5_25 based on maximum likelihood with 100 bootstrap replications. Only the GH5 catalytic domain is used for the phylogenetic analysis. All bootstrap values are displayed. The enzymes with a solved 3-D structure are marked with a red star.

Figure GH5_26

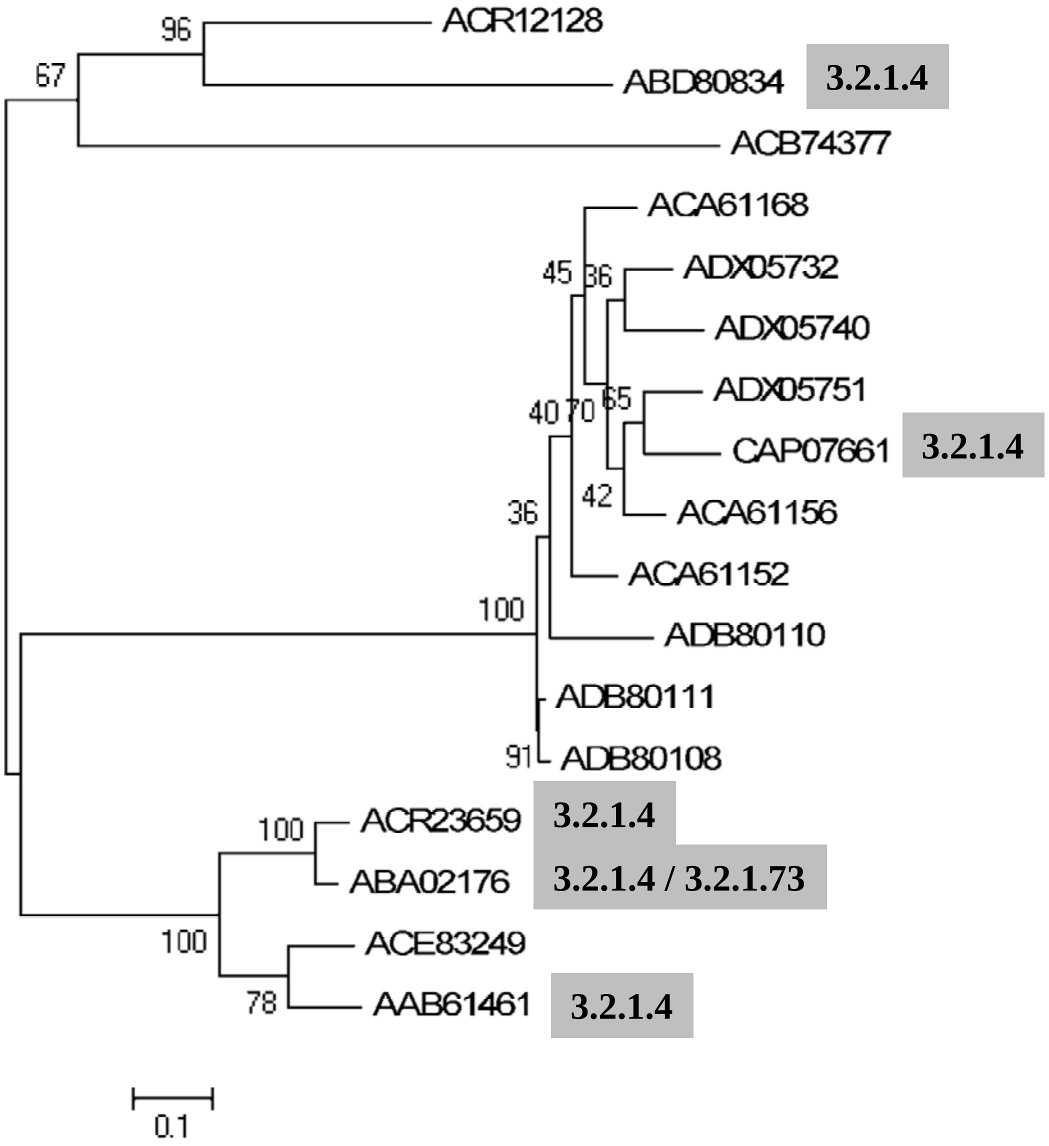

Figure GH5_26:

Phylogenetic tree on subfamily GH5_26 based on maximum likelihood with 100 bootstrap replications. Only the GH5 catalytic domain is used for the phylogenetic analysis. All bootstrap values are displayed.

Figure GH5_27

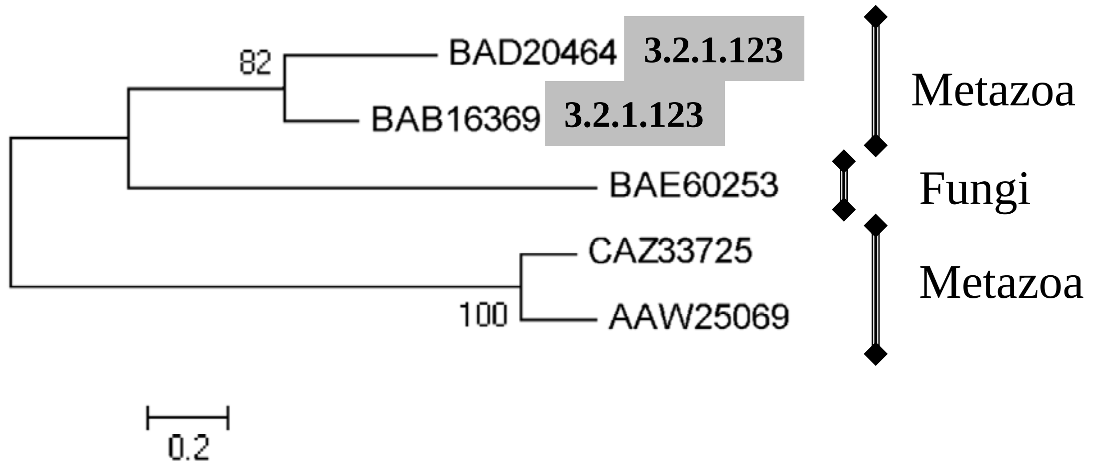

Figure GH5_27:

Phylogenetic tree on subfamily GH5_27 based on maximum likelihood with 100 bootstrap replications. Only the GH5 catalytic domain is used for the phylogenetic analysis. All bootstrap values are displayed.

Figure GH5_28

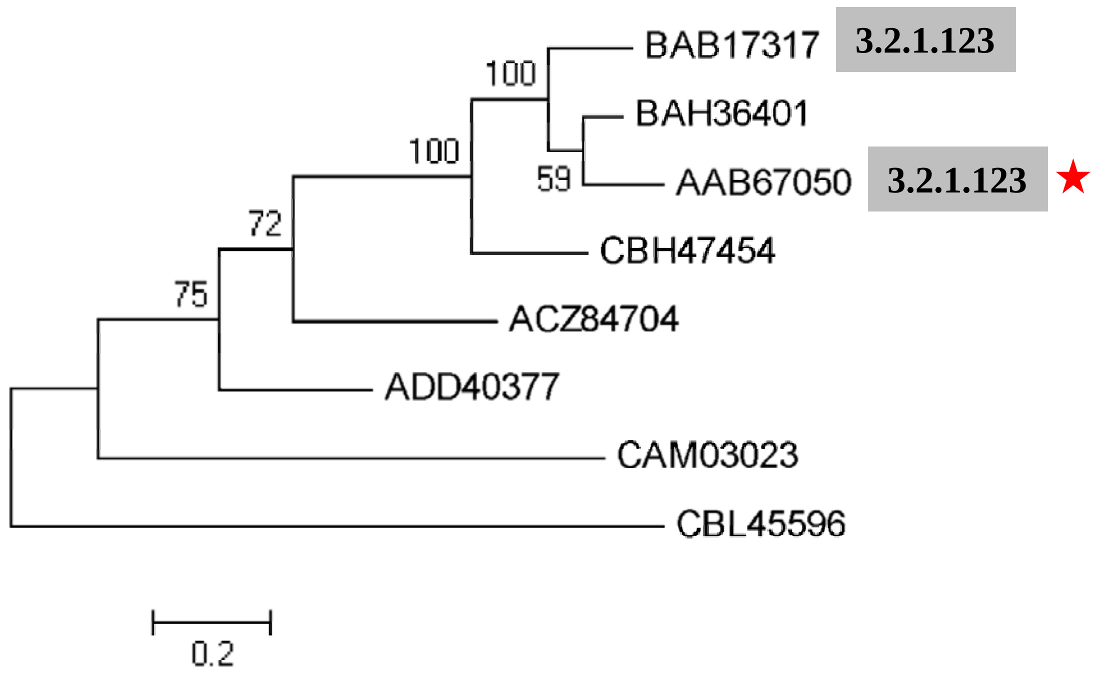

Figure GH5_28:

Phylogenetic tree on subfamily GH5_28 based on maximum likelihood with 100 bootstrap replications. Only the GH5 catalytic domain is used for the phylogenetic analysis. All bootstrap values are displayed. The only enzymes with a solved 3-D structure is marked with a red star.

Figure GH5_29

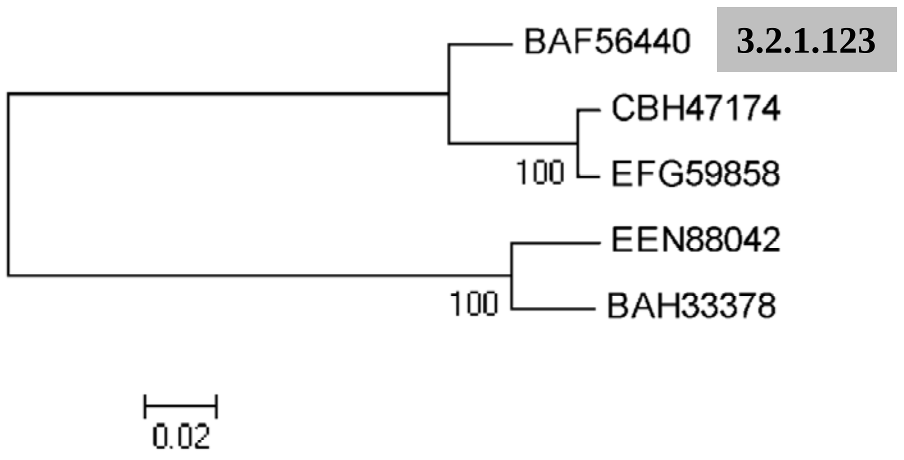

Figure GH5_29:

Phylogenetic tree on subfamily GH5_29 based on maximum likelihood with 100 bootstrap replications. Only the GH5 catalytic domain is used for the phylogenetic analysis. All bootstrap values are displayed.

Figure GH5_30

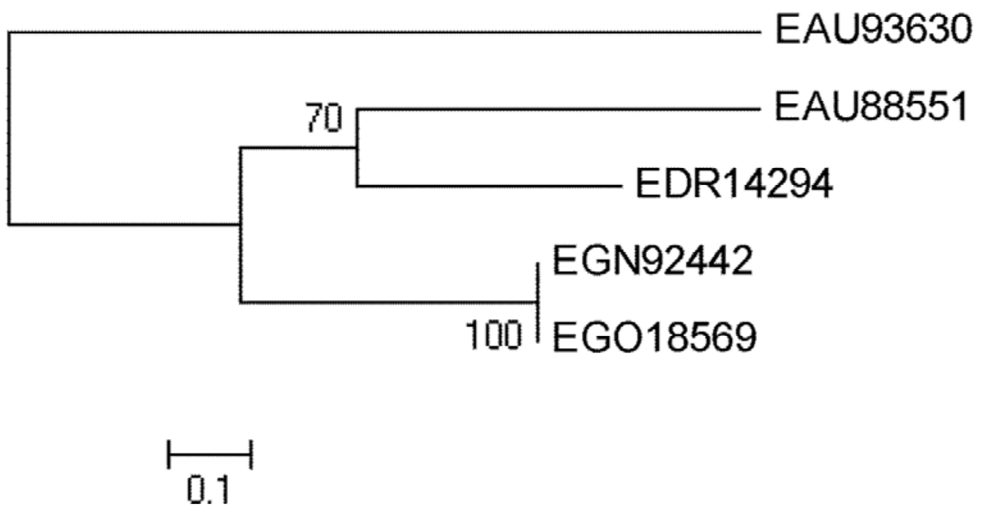

Figure GH5_30:

Phylogenetic tree on subfamily GH5_30 based on maximum likelihood with 100 bootstrap replications. Only the GH5 catalytic domain is used for the phylogenetic analysis. All bootstrap values are displayed.

Figure GH5_31

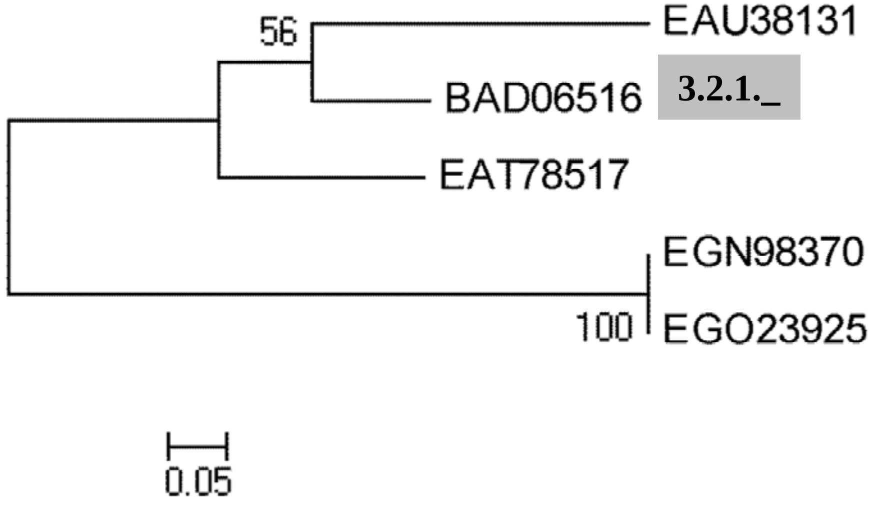

Figure GH5_31:

Phylogenetic tree on subfamily GH5_31 based on maximum likelihood with 100 bootstrap replications. Only the GH5 catalytic domain is used for the phylogenetic analysis. All bootstrap values are displayed.

Figure GH5_32

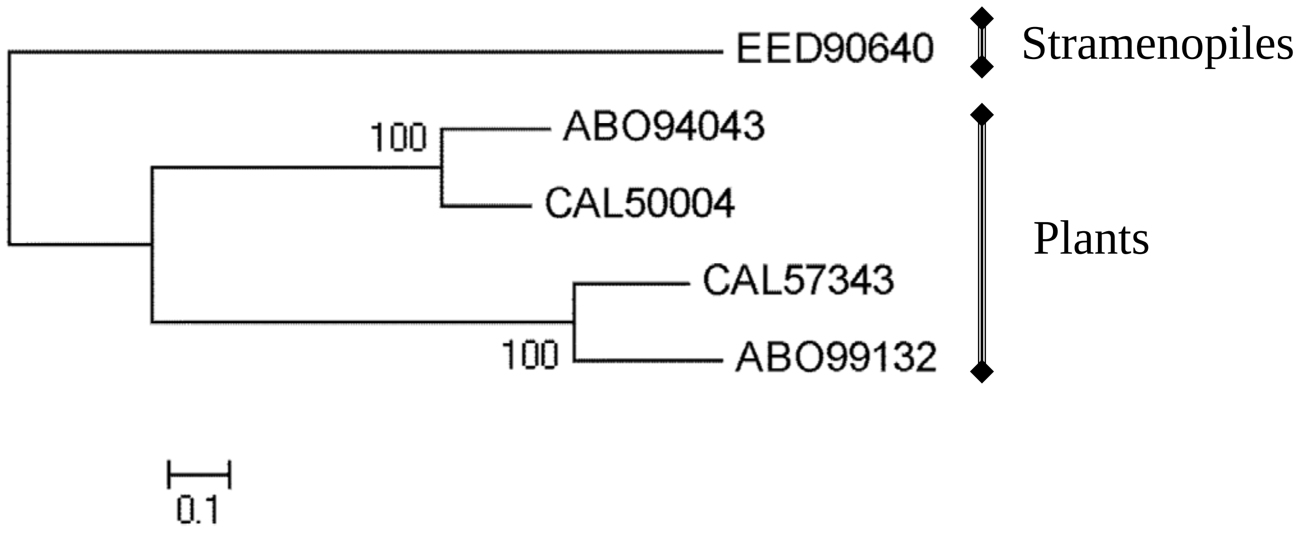

Figure GH5_32:

Phylogenetic tree on subfamily GH5_32 based on maximum likelihood with 100 bootstrap replications. Only the GH5 catalytic domain is used for the phylogenetic analysis. All bootstrap values are displayed.

Figure GH5_33

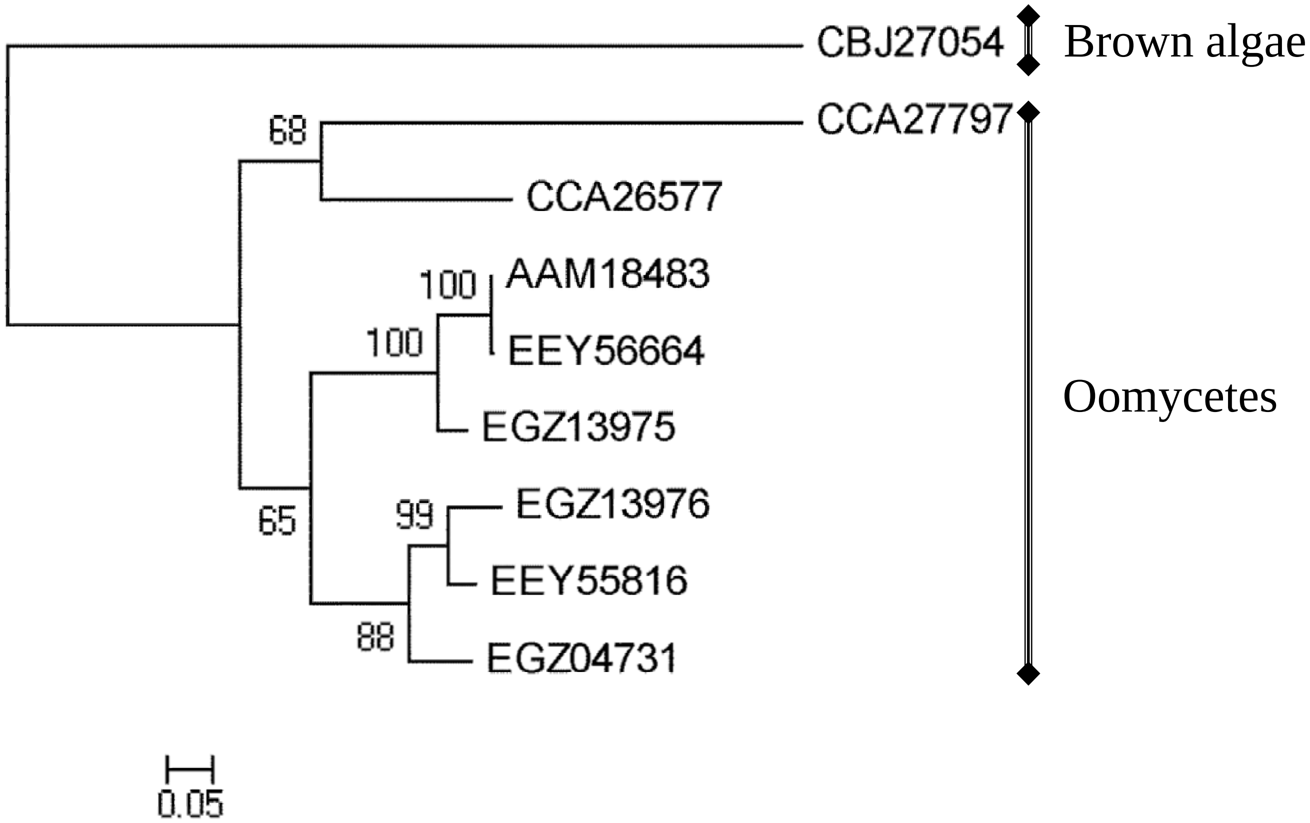

Figure GH5_33:

Phylogenetic tree on subfamily GH5_33 based on maximum likelihood with 100 bootstrap replications. Only the GH5 catalytic domain is used for the phylogenetic analysis. All bootstrap values are displayed.

Figure GH5_34

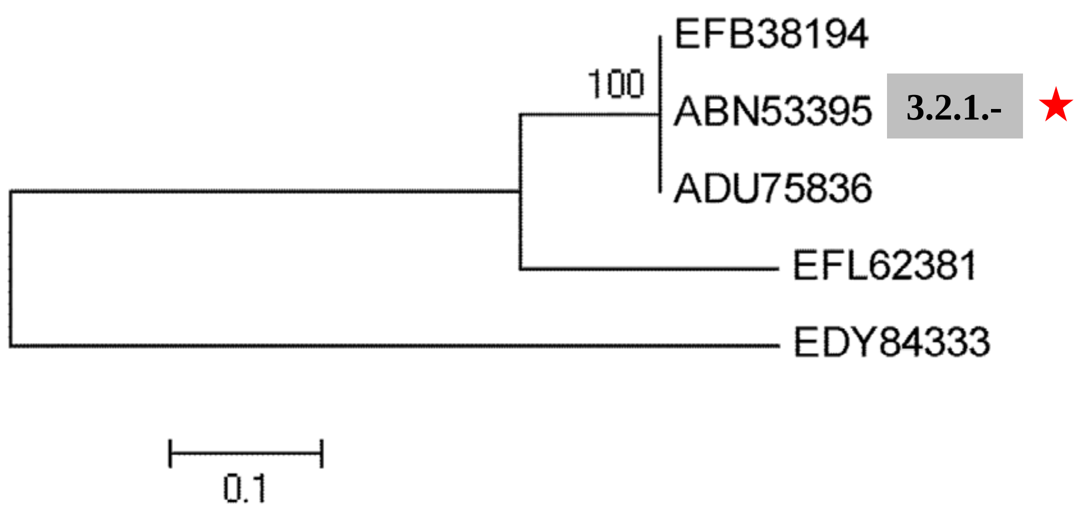

Figure GH5_34:

Phylogenetic tree on subfamily GH5_34 based on maximum likelihood with 100 bootstrap replications. Only the GH5 catalytic domain is used for the phylogenetic analysis. All bootstrap values are displayed. The only enzyme with a solved 3-D structure is marked with a red star.

Figure GH5_35

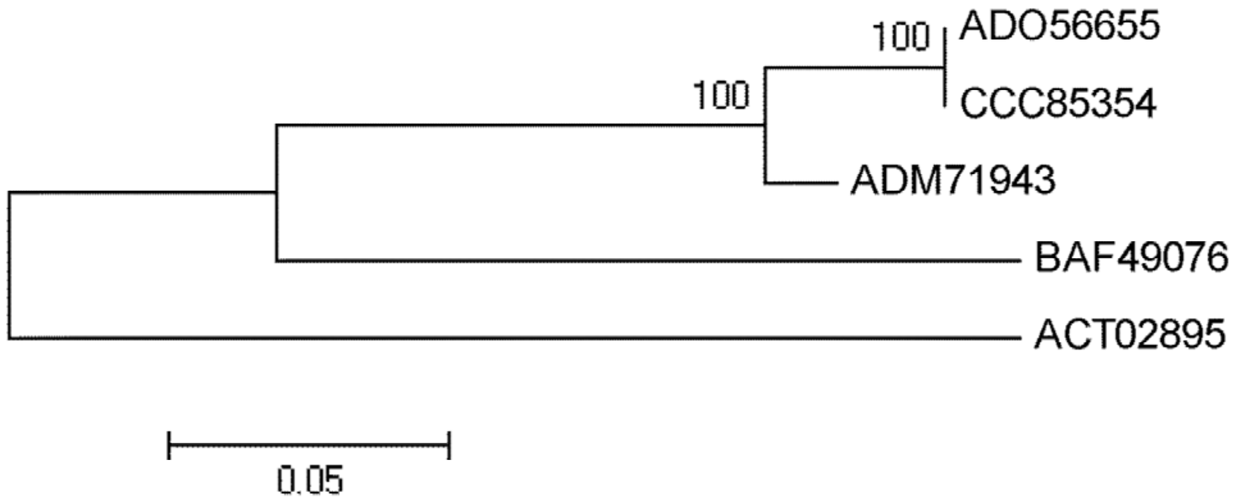

Figure GH5_35:

Phylogenetic tree on subfamily GH5_35 based on maximum likelihood with 100 bootstrap replications. Only the GH5 catalytic domain is used for the phylogenetic analysis. All bootstrap values are displayed.

Figure GH5_36

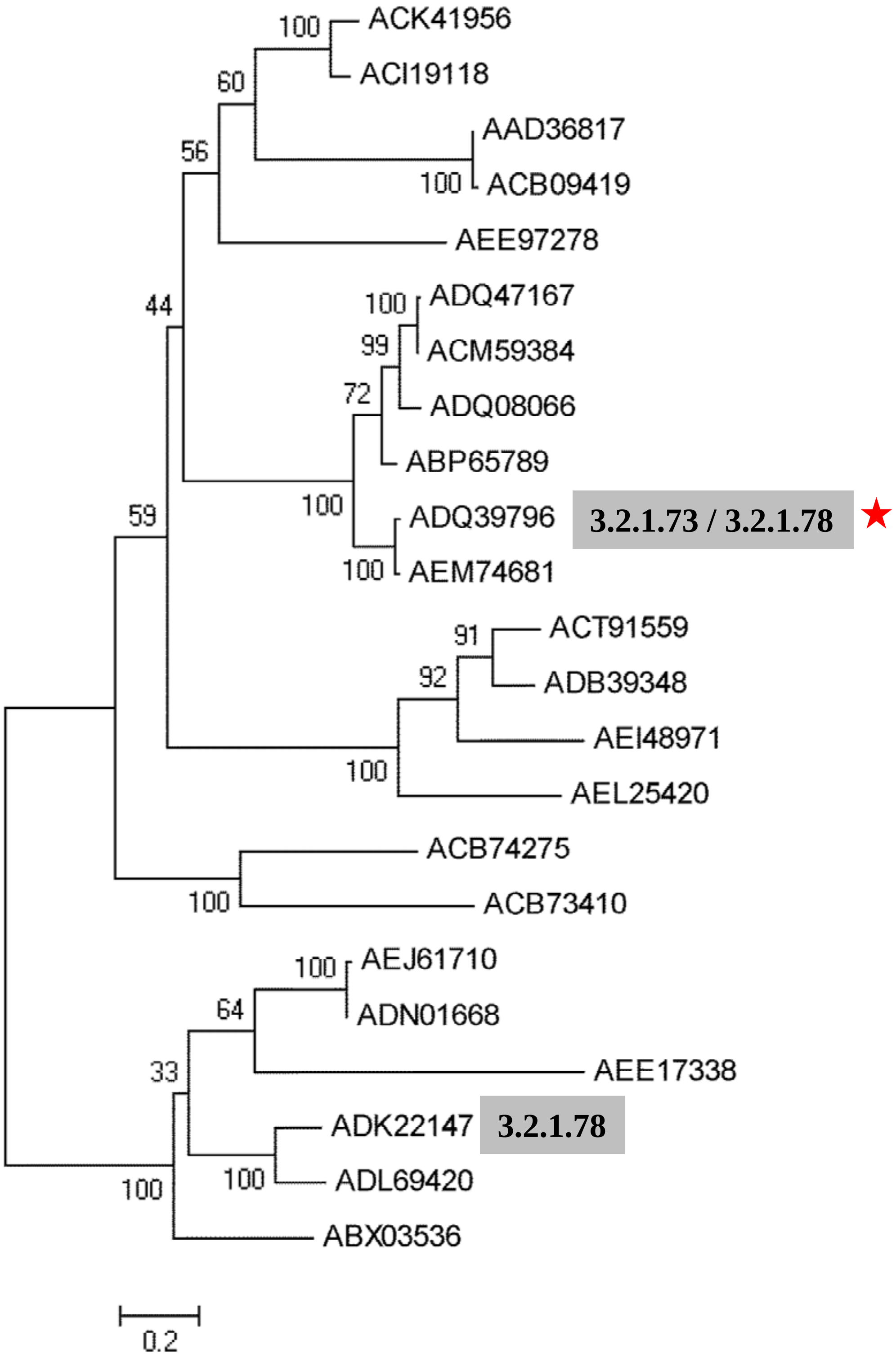

Figure GH5_36:

Phylogenetic tree on subfamily GH5_36 based on maximum likelihood with 100 bootstrap replications. Only the GH5 catalytic domain is used for the phylogenetic analysis. All bootstrap values are displayed. The only enzyme with a solved 3-D structure is marked with a red star.

Figure GH5_37

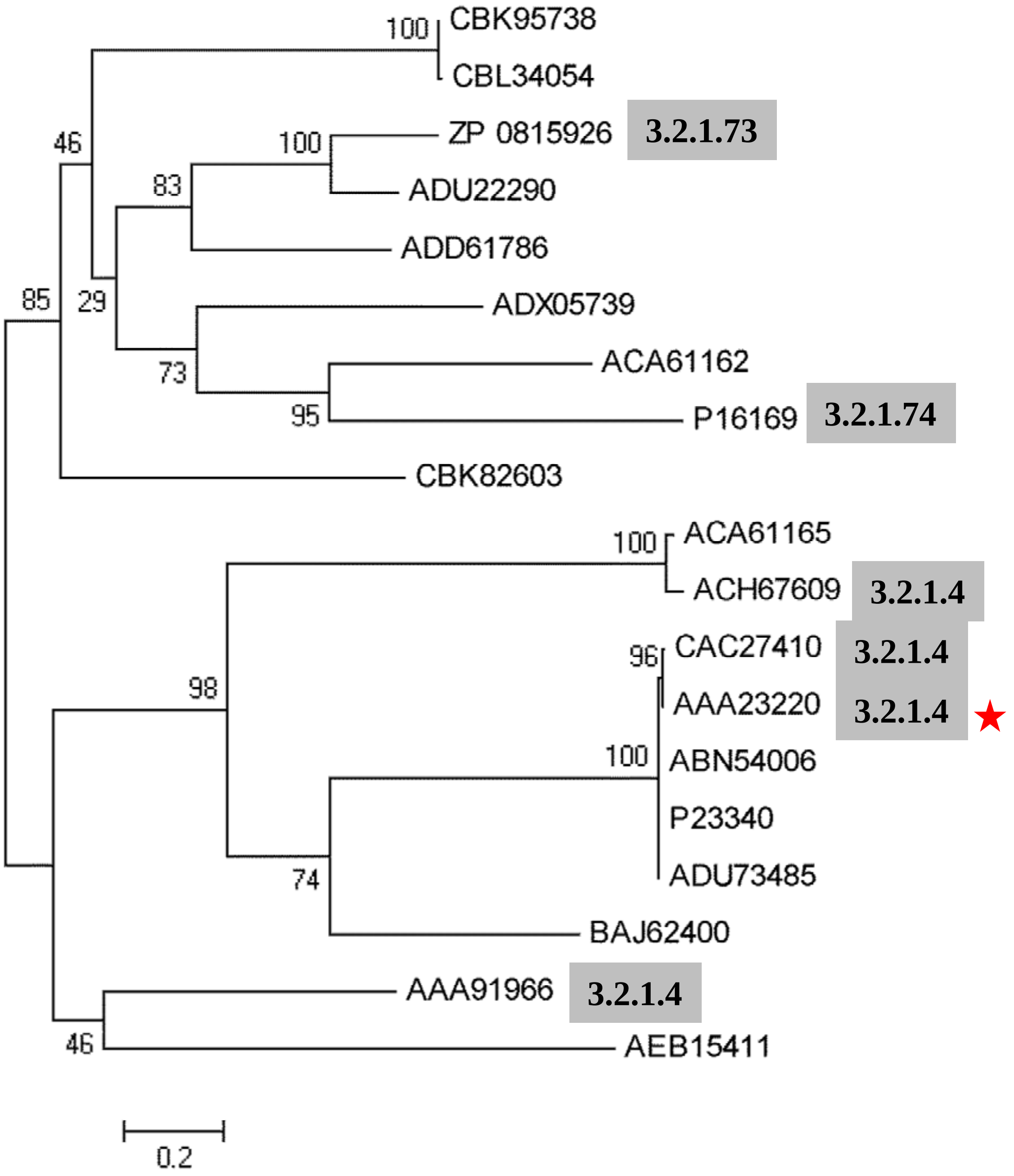

Figure GH5_37:

Phylogenetic tree on subfamily GH5_37 based on maximum likelihood with 100 bootstrap replications. Only the GH5 catalytic domain is used for the phylogenetic analysis. All bootstrap values are displayed. The only enzyme with a solved 3-D structure is marked with a red star.

Figure GH5_38

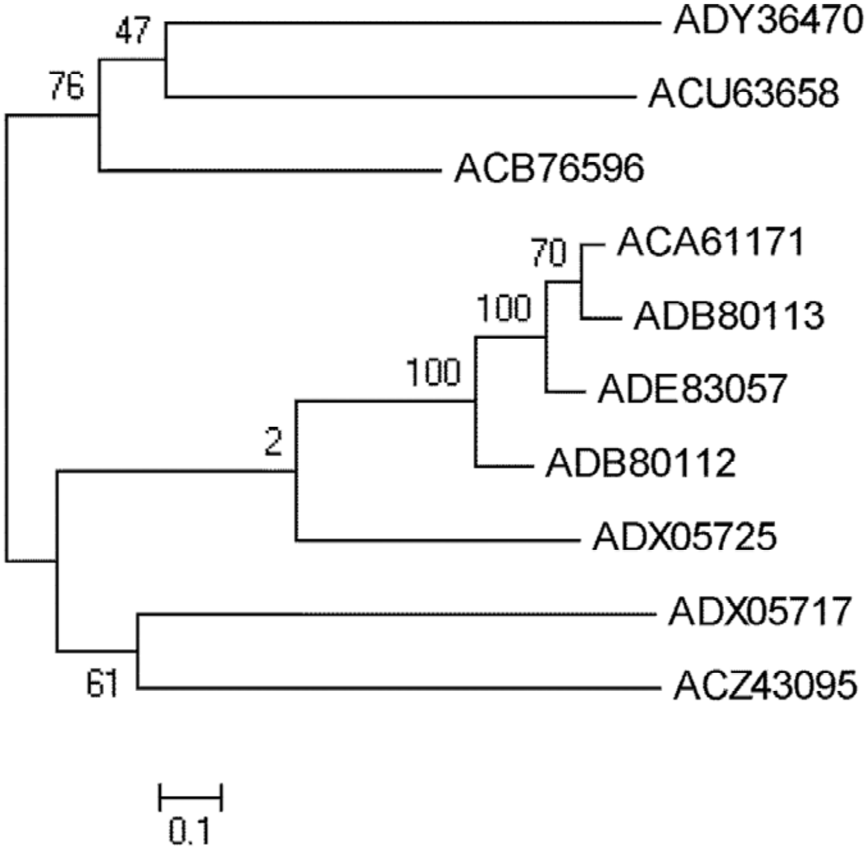

Figure GH5_38:

Phylogenetic tree on subfamily GH5_38 based on maximum likelihood with 100 bootstrap replications. Only the GH5 catalytic domain is used for the phylogenetic analysis. All bootstrap values are displayed.

Figure GH5_39

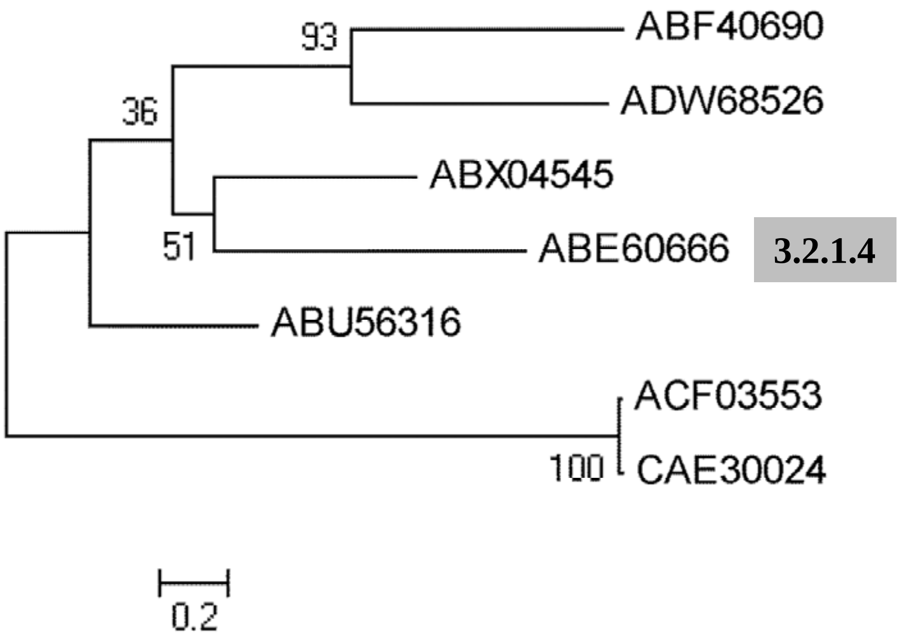

Figure GH5_39:

Phylogenetic tree on subfamily GH5_39 based on maximum likelihood with 100 bootstrap replications. Only the GH5 catalytic domain is used for the phylogenetic analysis. All bootstrap values are displayed.

Figure GH5_40

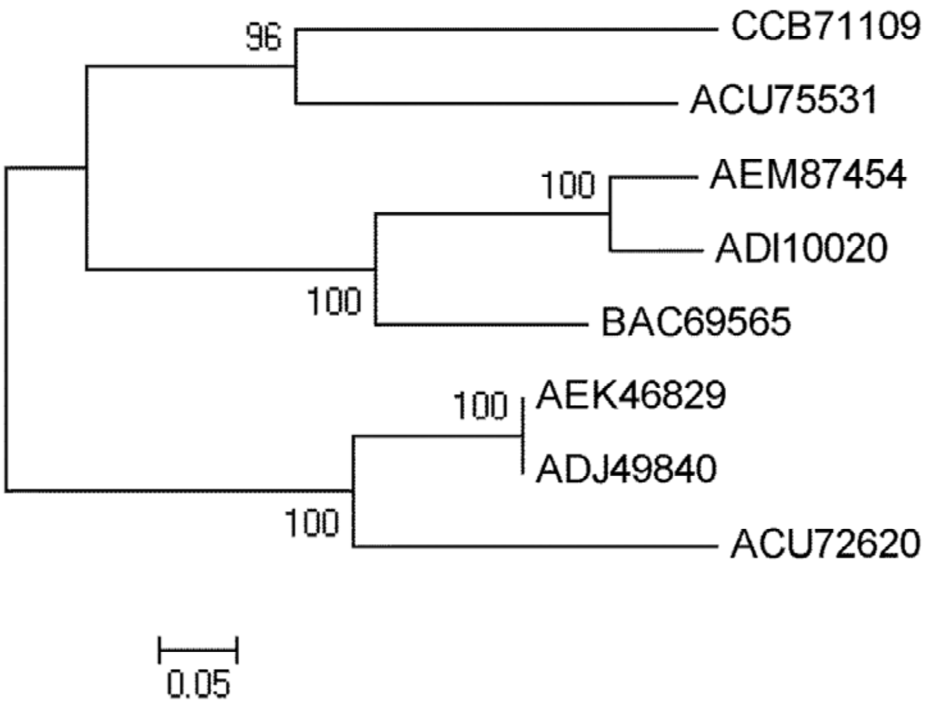

Figure GH5_40:

Phylogenetic tree on subfamily GH5_40 based on maximum likelihood with 100 bootstrap replications. Only the GH5 catalytic domain is used for the phylogenetic analysis. All bootstrap values are displayed.

Figure GH5_41

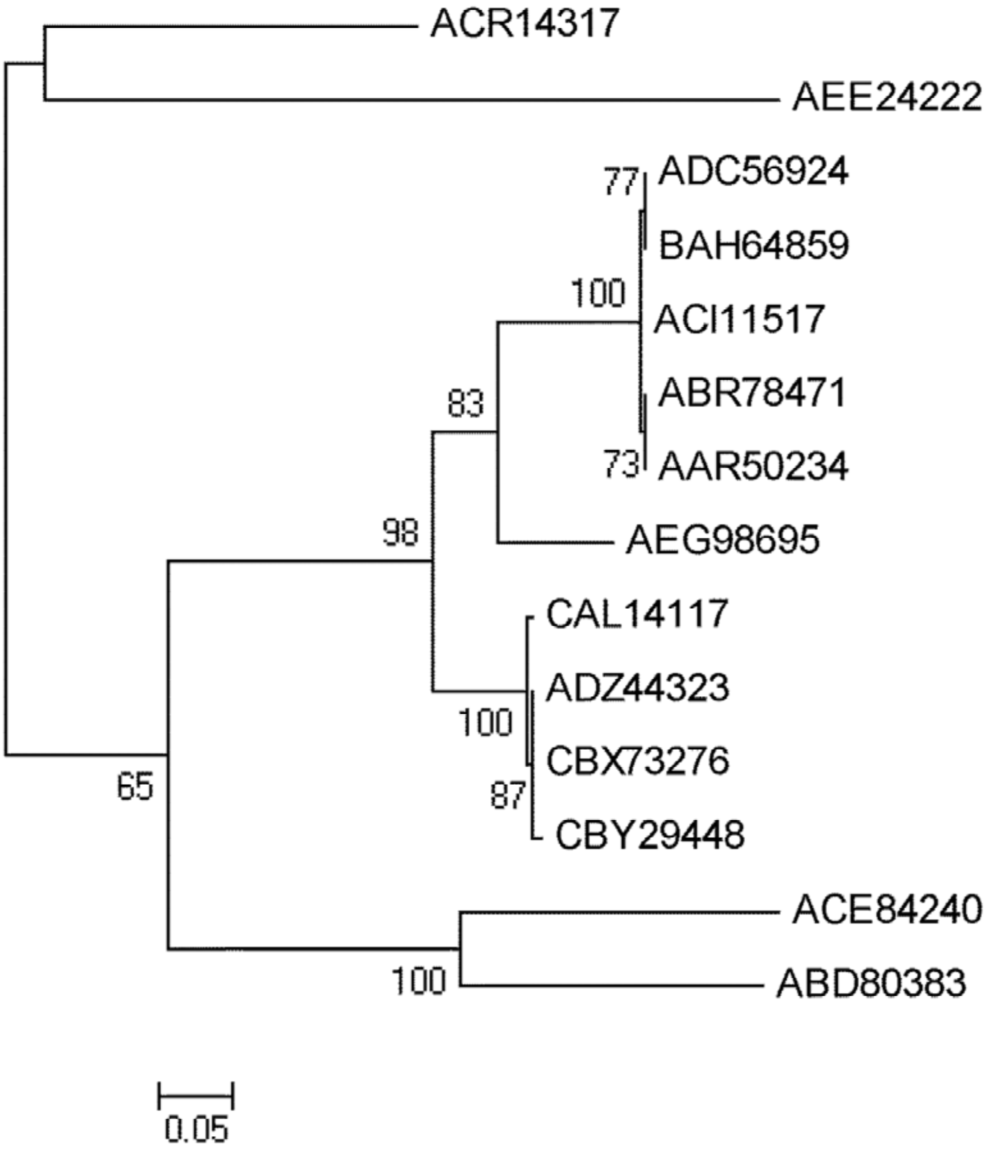

Figure GH5_41:

Phylogenetic tree on subfamily GH5_41 based on maximum likelihood with 100 bootstrap replications. Only the GH5 catalytic domain is used for the phylogenetic analysis. All bootstrap values are displayed.

Figure GH5_42

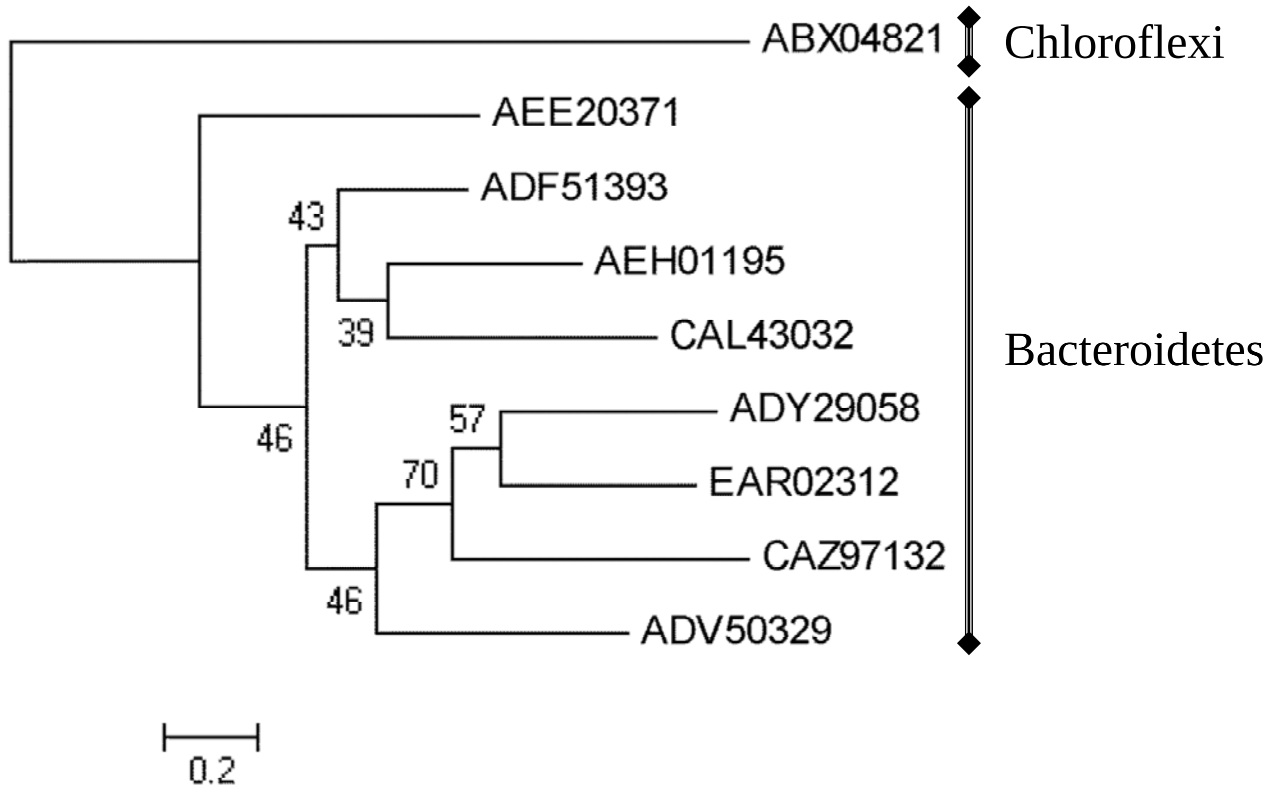

Figure GH5_42:

Phylogenetic tree on subfamily GH5_42 based on maximum likelihood with 100 bootstrap replications. Only the GH5 catalytic domain is used for the phylogenetic analysis. All bootstrap values are displayed.

Figure GH5_43

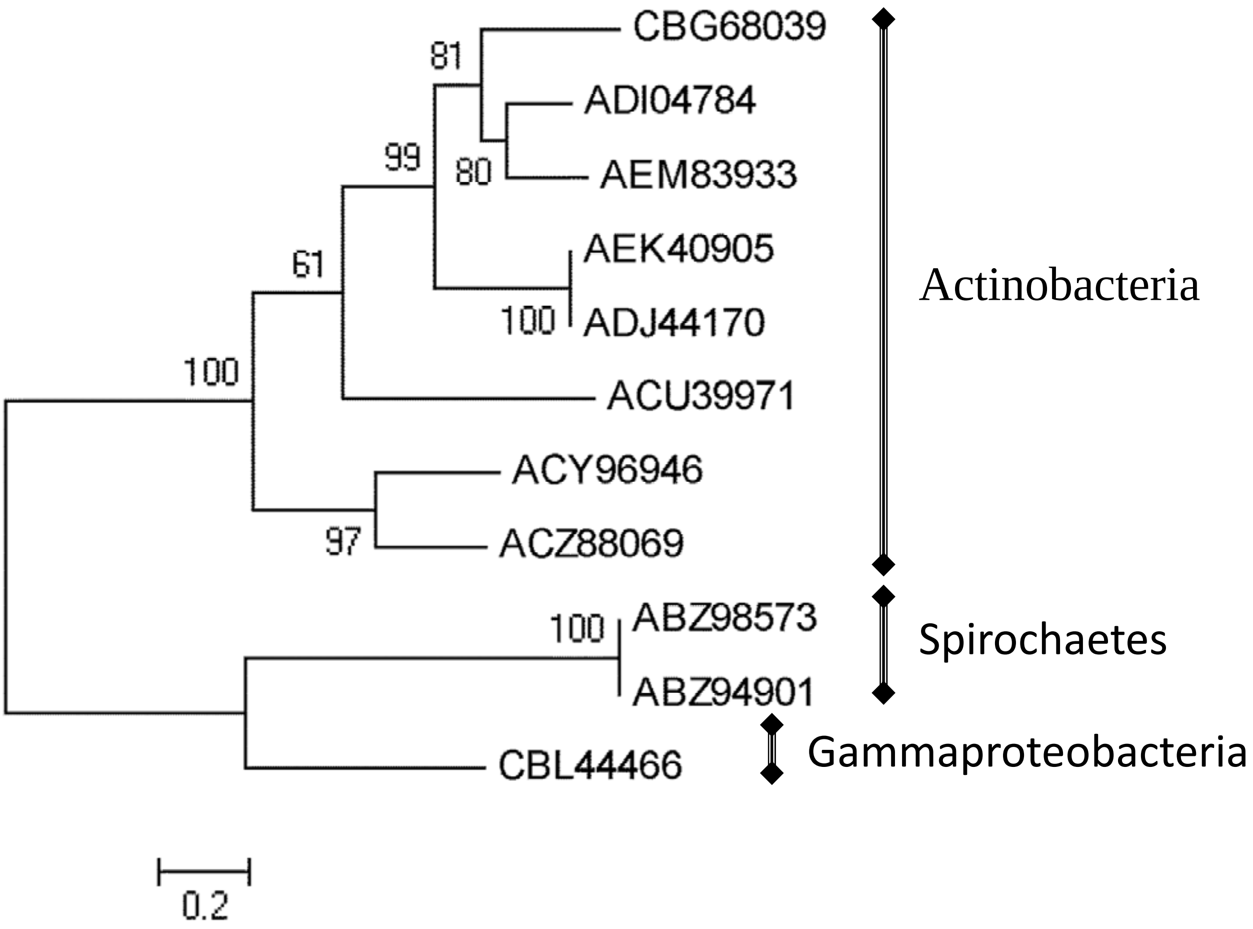

Figure GH5_43:

Phylogenetic tree on subfamily GH5_43 based on maximum likelihood with 100 bootstrap replications. Only the GH5 catalytic domain is used for the phylogenetic analysis. All bootstrap values are displayed.

Figure GH5_44

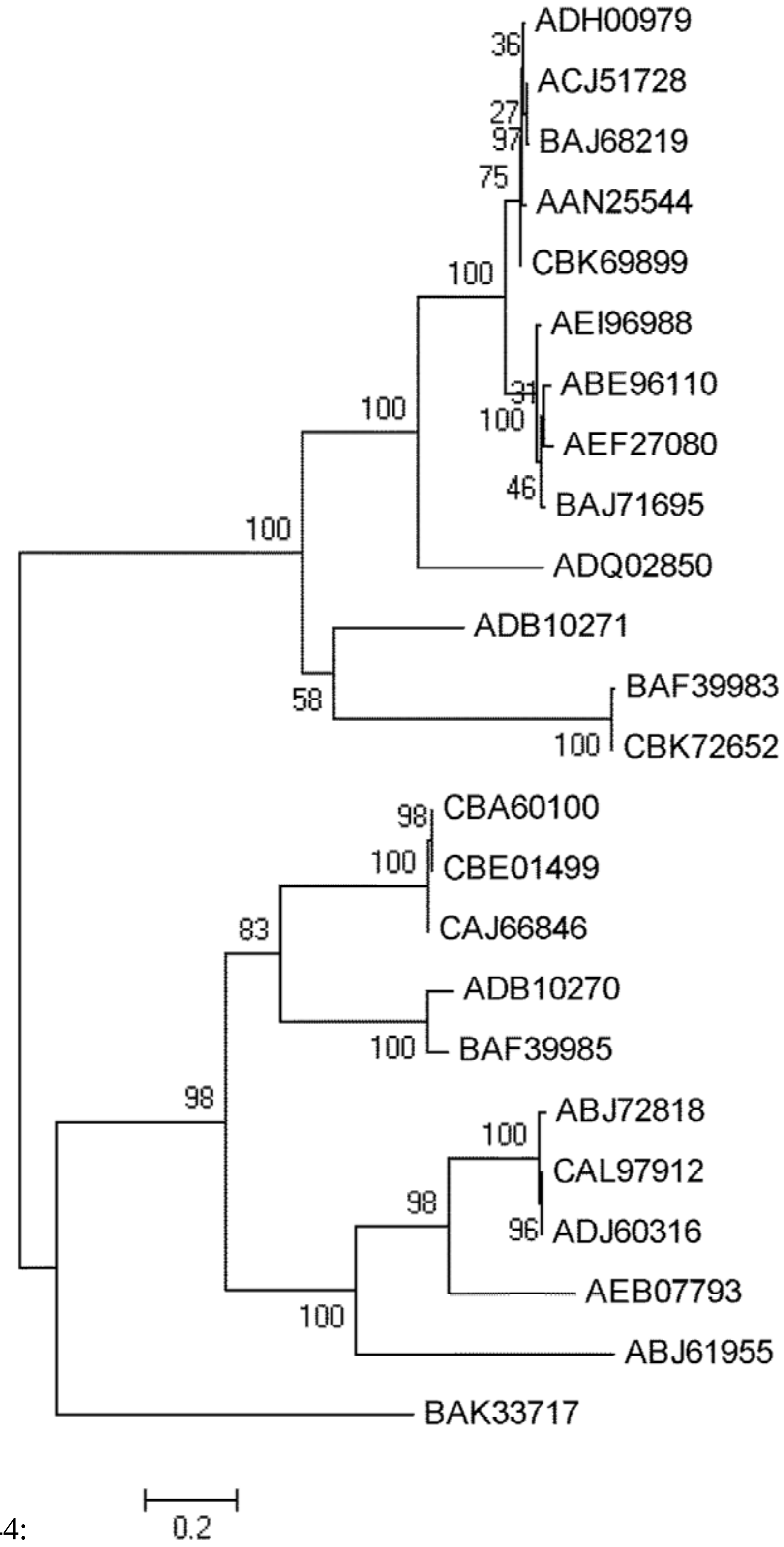

Figure GH5_44:

Phylogenetic tree on subfamily GH5_44 based on maximum likelihood with 100 bootstrap replications. Only the GH5 catalytic domain is used for the phylogenetic analysis. All bootstrap values are displayed.

Figure GH5_45

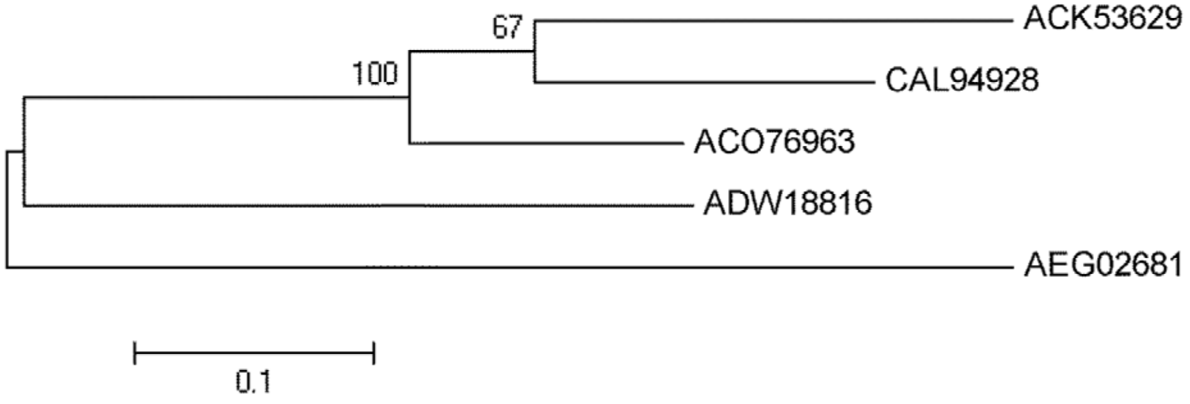

Figure GH5_45:

Phylogenetic tree on subfamily GH5_45 based on maximum likelihood with 100 bootstrap replications. Only the GH5 catalytic domain is used for the phylogenetic analysis. All bootstrap values are displayed.

Figure GH5_46

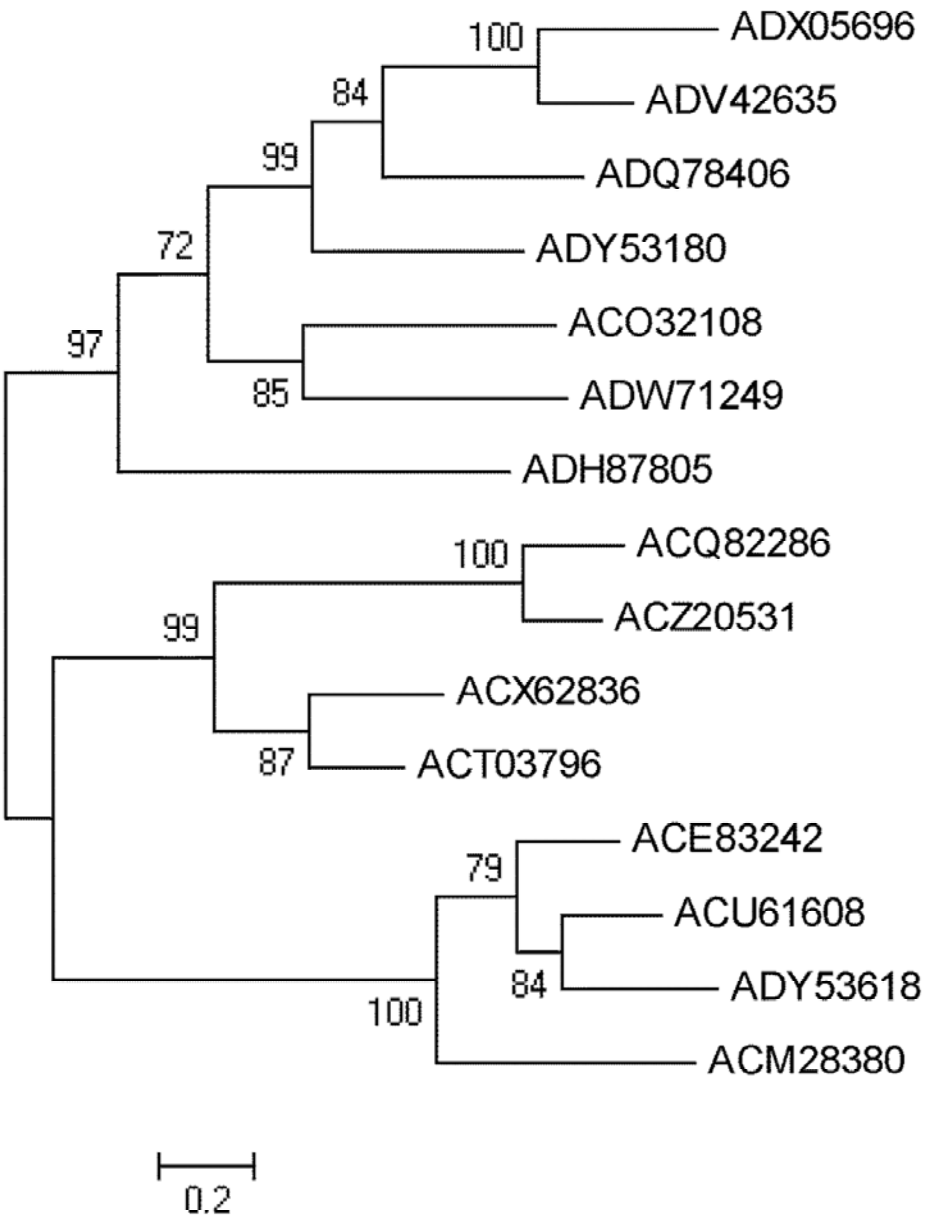

Figure GH5_46:

Phylogenetic tree on subfamily GH5_46 based on maximum likelihood with 100 bootstrap replications. Only the GH5 catalytic domain is used for the phylogenetic analysis. All bootstrap values are displayed.

Figure GH5_47

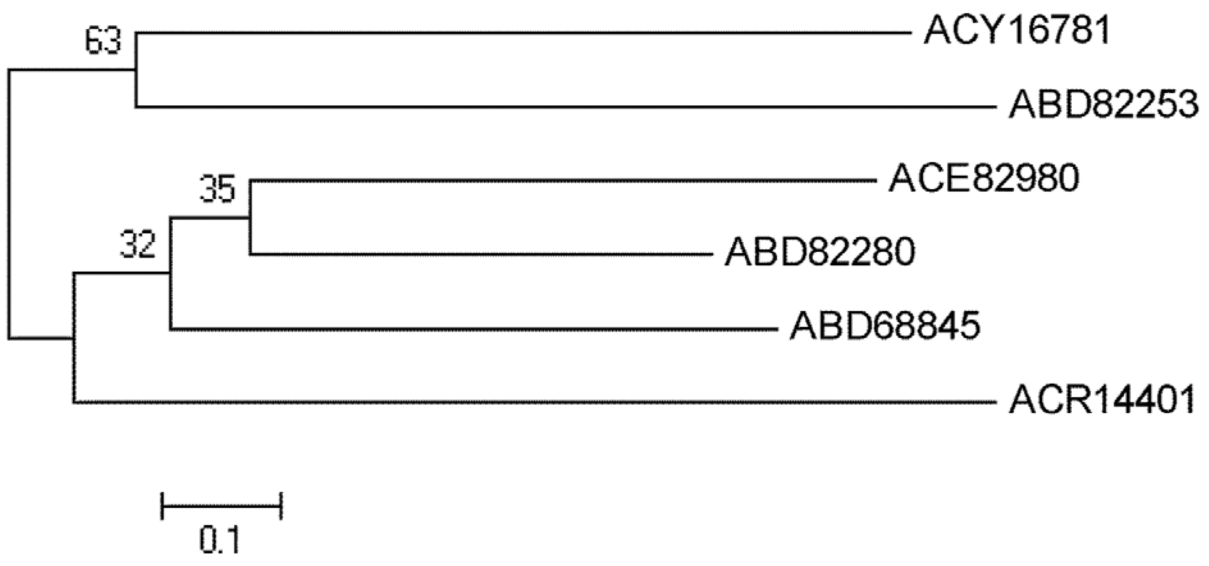

Figure GH5_47:

Phylogenetic tree on subfamily GH5_47 based on maximum likelihood with 100 bootstrap replications. Only the GH5 catalytic domain is used for the phylogenetic analysis. All bootstrap values are displayed.

Figure GH5_48

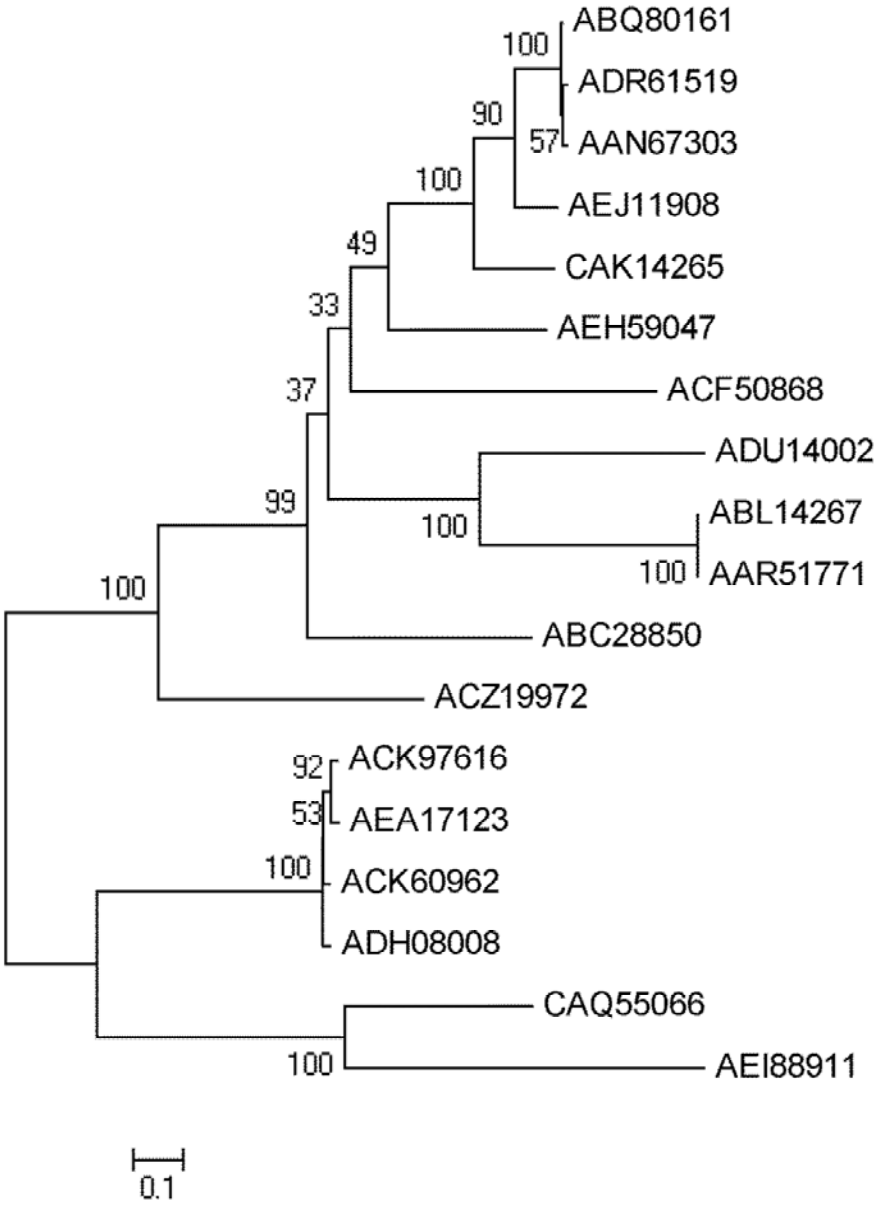

Figure GH5_48:

Phylogenetic tree on subfamily GH5_48 based on maximum likelihood with 100 bootstrap replications. Only the GH5 catalytic domain is used for the phylogenetic analysis. All bootstrap values are displayed.

Figure GH5_49

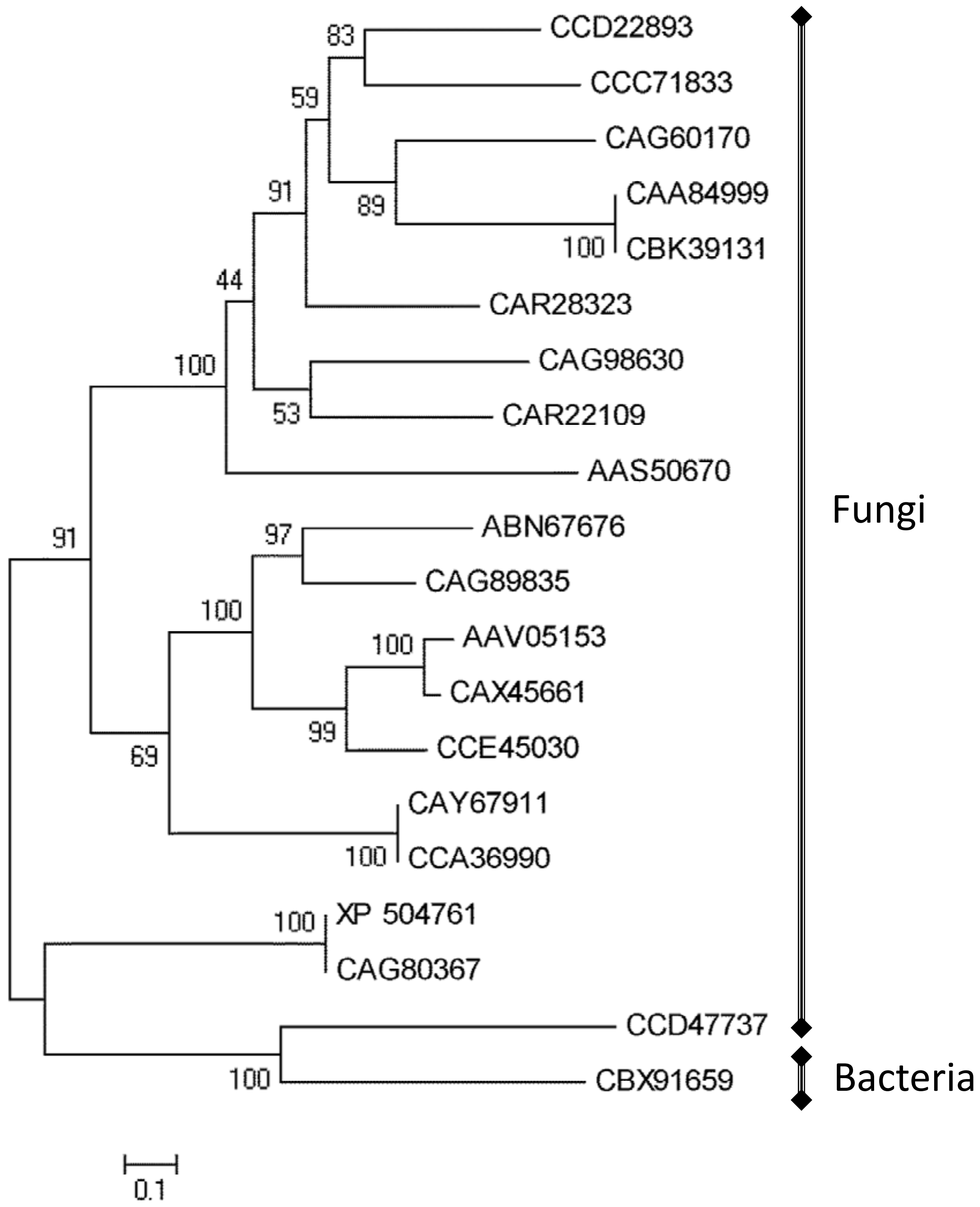

Figure GH5_49:

Phylogenetic tree on subfamily GH5_49 based on maximum likelihood with 100 bootstrap replications. Only the GH5 catalytic domain is used for the phylogenetic analysis. All bootstrap values are displayed.

Figure GH5_50

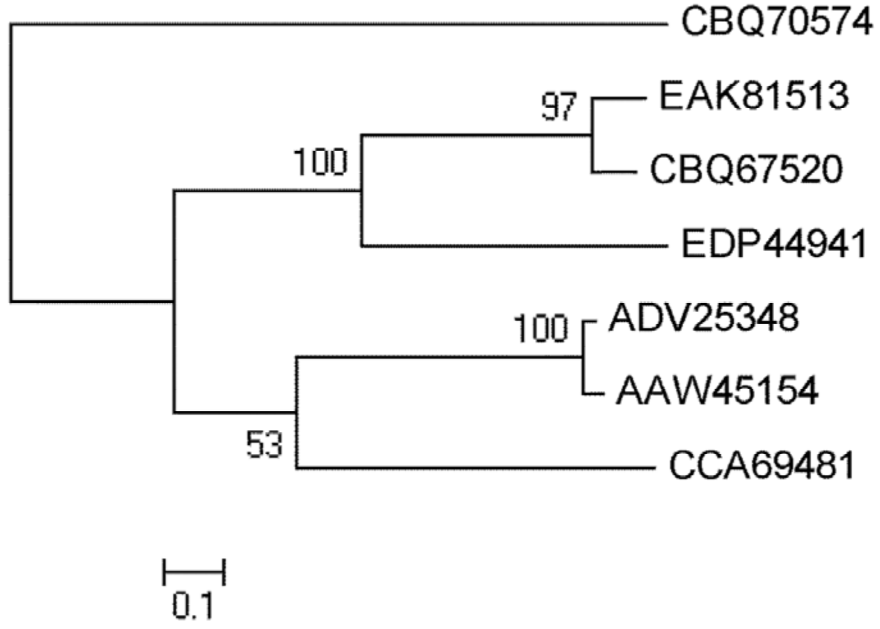

Figure GH5_50:

Phylogenetic tree on subfamily GH5_50 based on maximum likelihood with 100 bootstrap replications. Only the GH5 catalytic domain is used for the phylogenetic analysis. All bootstrap values are displayed.

Figure GH5_51

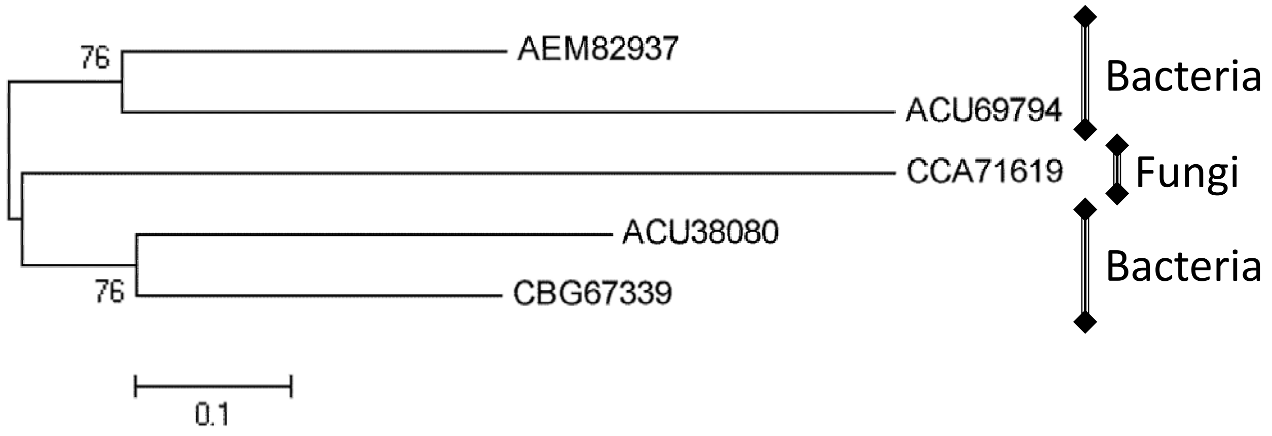

Figure GH5_51:

Phylogenetic tree on subfamily GH5_51 based on maximum likelihood with 100 bootstrap replications. Only the GH5 catalytic domain is used for the phylogenetic analysis. All bootstrap values are displayed.

Figure GH5_52

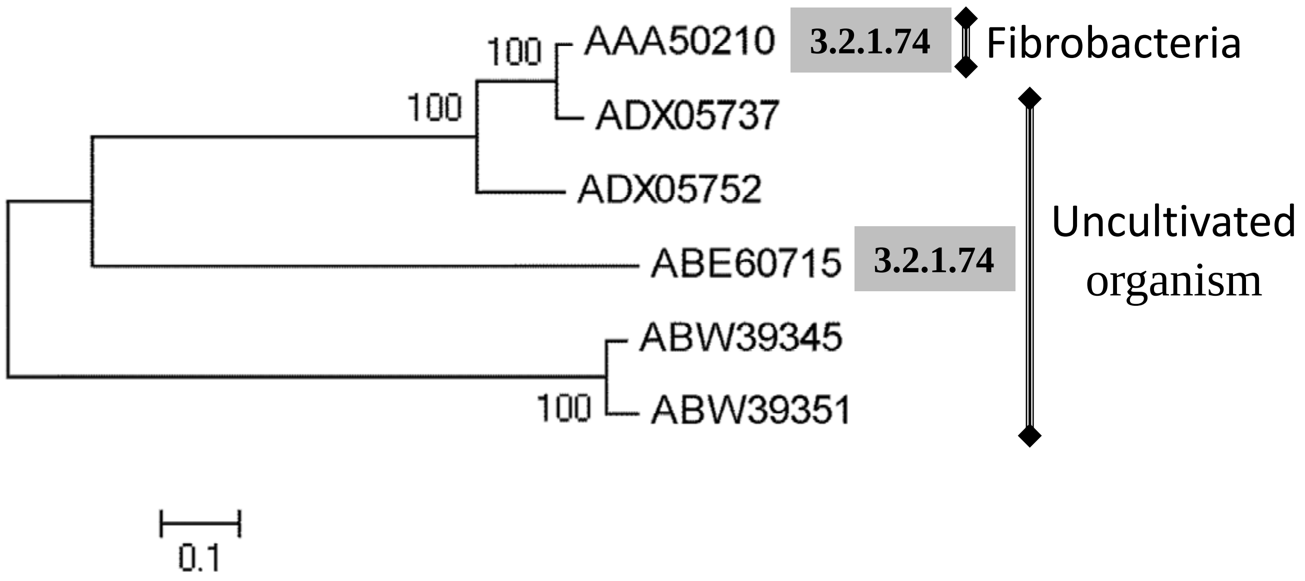

Figure GH5_52:

Phylogenetic tree on subfamily GH5_52 based on maximum likelihood with 100 bootstrap replications. Only the GH5 catalytic domain is used for the phylogenetic analysis. All bootstrap values are displayed.

Figure GH5_53

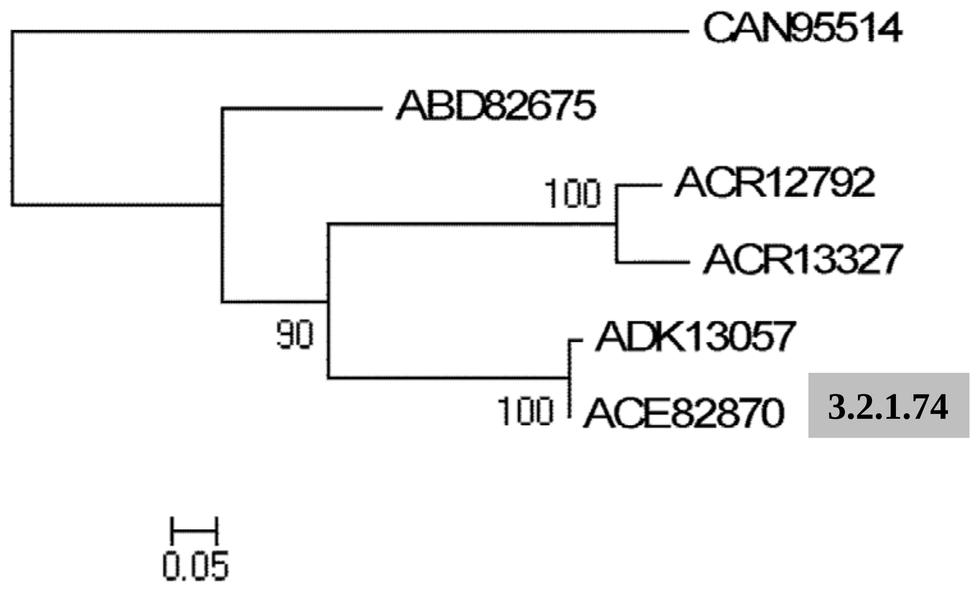

Figure GH5_53:

Phylogenetic tree on subfamily GH5_53 based on maximum likelihood with 100 bootstrap replications. Only the GH5 catalytic domain is used for the phylogenetic analysis. All bootstrap values are displayed.
